# The hepato- and neuroprotective effect of gold *Casuarina equisetifolia* bark nano-extract against Chlorpyrifos-induced toxicity in rats

**DOI:** 10.1186/s43141-023-00595-6

**Published:** 2023-12-01

**Authors:** Wael Mahmoud Aboulthana, Noha El-Sayed Ibrahim, Amgad Kamal Hassan, Wagdy Khalil Bassaly, Hassan Abdel-Gawad, Hamdy Ahmed Taha, Kawkab A. Ahmed

**Affiliations:** 1 Biochemistry Department, Biotechnology Research Institute, National Research Centre, 33 El Bohouth St. (Former El Tahrir St.), Dokki, P.O. 12622, Giza, Egypt; 2 Microbial Biotechnology Department, Biotechnology Research Institute, National Research Centre, 33 El Bohouth St. (Former El Tahrir St.), Dokki, P.O. 12622, Giza, Egypt; 3 Cell Biology Department, Biotechnology Research Institute, National Research Centre, 33 El Bohouth St. (Former El Tahrir St.), Dokki, P.O. 12622, Giza, Egypt; 4 Applied Organic Chemistry Department, Chemical Industries Researches Institute, National Research Centre, 33 El Bohouth St. (Former El Tahrir St.), Dokki, P.O. 12622, Giza, Egypt; 5 Department of Pathology, Faculty of Veterinary Medicine, Cairo University, Giza, 12211 Egypt

**Keywords:** Chlorpyrifos, *Casuarina equisetifolia* bark, Green nanotechnology, Gene expression, Electrophoretic isoenzymes

## Abstract

**Background:**

The bark of *Casuarina equisetifolia* contains several active phytoconstituents that are suitable for the biosynthesis of gold nanoparticles (Au-NPs). These nanoparticles were subsequently evaluated for their effectiveness in reducing the toxicity induced by Chlorpyrifos (CPF) in rats.

**Results:**

Various hematological and biochemical measurements were conducted in this study. In addition, markers of oxidative stress and inflammatory reactions quantified in liver and brain tissues were evaluated. Histopathological examinations were performed on both liver and brain tissues. Furthermore, the native electrophoretic protein and isoenzyme patterns were analyzed, and the relative expression levels of apoptotic genes in these tissues were determined. The hematological and biochemical parameters were found to be severely altered in the group injected with CPF. However, the administration of Au-*C. equisetifolia* nano-extract normalized these levels in all treated groups. The antioxidant system markers showed a significant decrease (*P* ≤ 0.05) in conjunction with elevated levels of inflammatory and fibrotic markers in both liver and brain tissues of the CPF-injected group. In comparison, the pre-treated group exhibited a reduction in these markers when treated with the nano-extract, as opposed to the CPF-injected group. Additionally, the nano-extract mitigated the severity of histopathological lesions induced by CPF in both liver and brain tissues, with a higher ameliorative effect observed in the pre-treated group. Electrophoretic assays conducted on liver and brain tissues revealed that the nano-extract prevented the qualitative changes induced by CPF in the pre-treated group. Furthermore, the molecular assay demonstrated a significant increase in the relative expression of apoptotic genes in the CPF-injected rats. Although the nano-extract ameliorated the relative expression of these genes compared to the CPF-injected group, it was unable to restore their values to normal levels.

**Conclusion:**

Our results demonstrated that the nano-extract effectively reduced the toxicity induced by CPF in rats at hematological, biochemical, histopathological, physiological, and molecular levels, in the group pre-treated with the nano-extract.

**Supplementary Information:**

The online version contains supplementary material available at 10.1186/s43141-023-00595-6.

## Background

Several hundred pesticides have been synthesized to be used widely for reducing unwanted (harmful) insects and disease carriers to increase food production [1]. It was reported that Europe is thought to be the region that uses pesticides the most, followed by China then the USA [2]. Misuse of the pesticides (uncensored) leads to serious contamination that is harmful to human health due to their affinity to accumulate in the environment [3]. Consumption of vegetables and fruits grown in pesticide-contaminated soil affected the body organs and causes chronic diseases such as neurotoxicity, cardiac disease, diabetes, cancer, and asthma in addition to reproductive disorder [4]. The quaternary nitrogen compounds are associated with neurodegenerative diseases. Additionally, the cancer problem is caused by these compounds, but breast cancer is the most common among all cancer types and is associated with organophosphorus (OP) compounds that affect cellular growth and proliferation [5]. The OP compounds are part of a vast family of pesticides that have become widespread pollutants in the environment [6]. These compounds exert their deleterious biological effects through stimulating the oxidative stress and electrophilic attack of cellular constituents by overproduction of reactive oxygen species (ROS), which results in loss of cellular homeostasis due to membrane damage. They cause lipid peroxidation products to accumulate in several targeting tissues [7, 8]. The subchronic and chronic exposure to OP pesticides causes different histological alterations in the targeting tissues including liver, kidney, brain, lung, and testes [9].

Chlorpyrifos (*O*,*O*-diethyl-*O*-(3,5,6-trichloro-2-pyridyl) phosphorothionate, CPF) is a commonly used OP compound utilized under different registered trademarks around the world [10]. It caused a number of cellular alterations, including decreased synaptogenesis, problems with cell differentiation and genetic transcription, changes in DNA synthesis, and breakdown of the aqueous layer, which exacerbated DNA degradation [11].

Acetylcholinesterase (AChE) exhibits its biological activity in the nervous system by regulating the neuronal communication through hydrolyzing acetylcholine (the omnipresent neurotransmitter) in the synaptic cleft [12]. It is well known that OP pesticides impede AChE action, causing acetylcholine to accumulate in the synaptic cleft, where it then remains charged and prevents further nerve impulse transmission via the cleft and hence prevents the smooth transmission of nerve functions [13]. As a result, AChE activity is regarded as a biomarker of toxicity caused by OP compounds [14].

The public and medical experts are increasingly accepting herbal treatment [15]. Many previous studies were designed to appraise the efficacy of the plant extracts against the OP pesticide–induced oxidative injuries. It was documented that root of green tea [16], black tea [17], curcumin [18], *Paronychia argentea* [19], *Ginkgo biloba* extracts, and different plant oils [20] are rich in various active biomolecules exhibiting effectiveness in reducing the peroxidation reaction and rebuilding the antioxidant system after being exposed to OP pesticides, which caused oxidative stress [21].

*Casuarina equisetifolia* bark contains a variety of active phytoconstituents that contribute to the pharmacological actions. These constituents include carbohydrates, alkaloids, proteins, glycosides, saponins, phenolics, flavonoids, steroids, reducing sugars, and triterpenoids [22, 23] in addition to the condensed tannins, which mostly consisted of procyanidin mixed with prodelphinidin and propelargonidin and had polymer chain lengths ranging from trimers to tridecamers of epicatechin (the main extension unit) [24]. It is well known by its antihyperlipidemic [25], hepatoprotective [26], nephroprotective [27], gastroprotective [28], antidiabetic and scavenging activities [24] in addition to the antimicrobial activities [29].

Bioavailability and efficiency of the most active phytoconstituents decreased due to their large molecular weights which affect their absorption through cellular membranes. Nanotechnology is used to solve such problems, where it increases their efficiency, bioavailability, stability, and solubility; decreases their toxicities; and achieves their release to the appropriate site of action [30]. The development of nano-extracts incorporated with these particles is considered as the most promising solution to the inherent stability issue of metal nanoparticles (M-NPs). Compared to native plant extracts alone, the plant extracts combined with the M-NPs had better antioxidant activity at lower doses because the total phenolic components were increased [31, 32]. In 2022, Aboulthana et al. [33] reported that total methanolic extract of *C. equisetifolia* bark was the most effective (suitable) extract for biosynthesis of gold nanoparticles (Au-NPs) and it was found that the extract demonstrated more in vitro biological activity after incorporating Au-NPs than the native extract alone. Therefore, the current study was designed to explore the efficiency of *C. equisetifolia* bark extract incorporated with Au-NPs against the adverse effect caused by OP compounds in rats.

## Methods

### Administration of Au-*C. equisetifolia* nano-extract

Based on our previous findings [33], the in vitro phytochemical and biological measurements of total methanolic extract and its successive (polar and non-polar) fractions showed that total methanolic extract possessed the highest biological activity. Therefore, it was chosen to be used for biosynthesis of Au-NPs during preparation of Au-*C. equisetifolia* nano-extract. Furthermore, it was observed that value of the median lethal dose (LD_50_) of the nano-extract was about 9333.33 mg/kg and hence the most suitable dose used for in vivo study was about 933.33 mg/kg b.w. (1/10 of LD_50_). The nano-extract was administered orally by a stomach tube.

### Induction of toxicity by pesticides

Prior to treatment, animals were fasted for 18 h. The CPF solution (dissolved in dimethyl sulfoxide (DMSO)) was administered orally to rats through a stomach tube. Volume of the dose depends on weight of the animals, and the dose recommended to induce toxicity was about 1/10 of LD_50_ of the pesticide used [6]. It was found that the LD_50_ of CPF was about 229 mg/kg b.w. for rats [34]. Therefore, the dose administered orally was about 22.9 mg/kg b.w.

### Experimental design

Fifty mature male Wistar rats (weighing 120–150 g) were obtained and maintained in the Animal House, National Research Centre, Giza, Egypt under controlled environmental conditions (at 25 ± 2 °C) then provided with water *ad libitum* and standard food. They were randomly divided into five cages (ten per cage) as the following:

- Control group: Rats were fed with normal diet and received distilled water in parallel with DMSO for 28 days.
- Au-*C. equisetifolia* bark nano-extract-treated group: Rats were treated orally with Au-*C. equisetifolia* bark nano-extract orally for 21 days.
- CPF-treated (toxic) group: Rats were treated orally with CPF for 28 days.
- Toxic group pre-treated with gold nano-extract: Rats were treated with nano-extract for 21 days followed by treating with CPF for another 28 days.
- Toxic group post-treated with gold nano-extract: Rats were treated with CPF for 28 days followed by treating with nano-extract for another 21 days.

### Collection of blood samples and tissues

The last treatment dose was administered after fasting for 18 h. The animals were then anesthetized using ketamine (100 mg/ml) and subsequently sacrificed by cervical dislocation. Blood samples were withdrawn from the retro-orbital plexus into heparinized tubes for hematological measurements and to determine the activity of the AChE enzyme. Additional blood samples were allowed to clot and then centrifuged at 3000 rpm for 15 min. The separated sera were stored at − 20 °C until biochemical assays could be conducted. Liver and brain tissues were removed from the animals and washed in ice-cold saline. A few autopsied pieces of these tissues were preserved in a neutral-buffered formalin solution (10%) for histopathological investigation. The remaining portion of the tissues was homogenized in potassium phosphate buffer (pH 7.4) and then centrifuged at 3000 rpm for 10 min. The clear supernatants were stored at − 80 °C for use in biochemical assays. For molecular analysis, the last part of the tissues was rapidly frozen using liquid nitrogen.

### Body weight gain and relative organ weight assays

#### Body weight and body weight gain assay

Body weights of all treated groups were measured using a sensitive balance throughout the treatment period for 30 consecutive days, once at the beginning of the experiment, once after 15 days of injection, and once on the day of sacrifice (i.e., after 30 days). The biological value of Au-*C. equisetifolia* nano-extract was assessed by determining the effect of the nano-extract on body weight gain using the formula proposed by Al-Attar [35] at the end of each experimental period.

#### Relative organ weight assay

After exsanguinations, all animals were euthanized. After removing liver and brain tissues, the tissues were rinsed with saline, dried, and then weighed (absolute organ weight). As suggested by Al-Attar and Al-Rethea [36], specific formula was used for calculating the relative organ weight to body weight of each animal.

### Hematological measurements

The hematological parameters, including indices of red blood cells (hemoglobin (HB), red blood cells (RBCs), hematocrit (HCT), mean corpuscular volume (MCV), mean corpuscular hemoglobin (MCH), mean corpuscular hemoglobin concentration (MCHC), red blood cell distribution width (RDW), mean platelet volume (MPV), and platelet (PLT) count), white blood cells (WBCs), and differential blood cells (lymphocytes, monocytes, and granulocytes), were quantified in heparinized blood samples using an automatic blood analyzer (ABX Micros 60 manufactured by HORIBA ABX SAS). Moreover, activity of AChE enzyme was determined in plasma and RBC specimens using the Ellman method which was documented by Ellman et al. [37] and modified by Gorun et al. [38].

### Biochemical measurements

All the conventional biochemical parameters (liver [alanine aminotransferase (ALT), aspartate aminotransferase (AST), alkaline phosphatase (ALP), and gamma-glutamyl aminotransferases (GGT)], heart enzymes [creatine kinase (CK) and lactate dehydrogenase (LDH)], kidney functions [urea, creatinine, and uric acid], protein [total protein (TP) and albumin (Alb)], and lipid profiles [total cholesterol (TC), triglycerides (TGs), and high-density lipoprotein cholesterol (HDL-c)]) were spectrophotometrically evaluated in sera specimens using the colorimetric kits (Spectrum Diagnostics Egyptian Company for Biotechnology, Cairo, Egypt). Furthermore, the formula that was suggested by Schumann and Klauke [39] used for calculating low-density lipoprotein cholesterol (LDL-c).

### Biochemical assays in supernatants of tissue homogenates

#### Markers of the oxidative stress

The total antioxidant capacity (TAC) [40] and reduced glutathione (GSH) [41] were assayed in liver and brain tissue homogenates. Activities of superoxide dismutase (SOD) [42], catalase (CAT) [43], and glutathione peroxidase (GPx) [44] were determined as unit per gram of tissue. Concentrations of lipid peroxidation product (LPO) [45] and total protein carbonyl (TPC) content [46] were also measured.

#### Inflammatory and fibrotic markers

The quantitative sandwich enzyme immunoassay technique was used for quantifying levels of tumor necrosis factor-α (TNF-α) [47] and interleukin-6 (IL-6) [48] that were expressed as pg/g in supernatants of tissue homogenates. AChE activity was determined in tissue homogenates by the Ellman method. Level of the hydroxyproline that was used as a fibrotic biomarker was assessed in liver tissue homogenates using the method suggested by Reddy and Enwemeka [49] as kg/mg tissue. In brain tissue homogenates, the β-amyloid (Aβ) content was determined using ELISA kits (USCN Life Science, Inc.).

### Histopathological examination

The preserved liver and brain specimens were used for preparing paraffin sections at the thickness of 5 µm then stained with hematoxylin and eosin (H&E) as proposed by Suvarna et al. [50]. A light microscope (BX43, Olympus) was used for inspecting the stained sections that were photographed by the Olympus DP27 camera provided with cellSens dimension software (Olympus). Histopathological abnormalities in the liver were assessed and scored from 0 to 3 through determining the percentage of the lesions in five microscopically examined fields per animal (*n* = 5). No changes were indicated by the score of 0. The mild, moderate, and severe changes were indicated by the scores of 1, 2, and 3, respectively. The percentages were calculated according to the method suggested by Fouad and Ahmed [51] for determining the grade (mild changes (< 30%), moderate changes (< 30–50%), and severe changes (> 50%)). Neuropathologic damage was graded from 0 to 4. No changes were indicated by the grade of 0 while the grades of 1, 2, 3, and 4 indicated the percentage of affected area as follows: < 10%, 20–30%, 40–60%, and > 60%, respectively [52].

### Electrophoretic assays

#### Native electrophoretic patterns

Known weights (0.2 g) of the tissue (liver and brain) were homogenized in extraction buffer (1 ml) then centrifuged. From each group, equal amounts of the individual (clear) supernatants were pooled together and used as a single sample and the total protein concentration was quantified in all mixed samples using the method documented by Bradford [53]. During the electrophoretic assays, all the samples were diluted with loading dye for making the protein concentrations equal in all wells.

The polyacrylamide gel electrophoresis (PAGE) was used for separating the native proteins that were stained with Coomassie brilliant blue (CBB) [54] for assaying the protein bands that appeared as blue bands, stained with Sudan Black B (SBB) [55] for assaying the lipid moiety of native protein that appeared as black bands, and stained with Alizarin Red S [56] for assaying the calcium moiety of native protein that appeared as yellow bands.

The native gel was incubated with hydrogen peroxide (H_2_O_2_) that was used as a substrate then stained with potassium iodide (KI) [57] for detecting the CAT types electrophoretically that appeared as yellow bands and stained with benzidine [58] for detecting the peroxidase (POX) types that appeared as brown bands. The electrophoretic α-amylase (α-Amy) pattern was processed by incubating the native gel with a substrate consisting of soluble starch solution then stained with iodine solution [59] for detecting the α-Amy types that appeared as yellow bands. The electrophoretic esterase (EST) patterns were assayed by incubating the native gel in conditioning buffer for optimizing the enzyme activity then stained with a reaction mixture containing Fast Blue RR (as a dye coupler) along with substrates consisting of an α- and β-naphthyl acetate solution for detecting the α- and β-EST types that appeared as brown and pink bands, respectively [60].

#### Data analysis

After photographing the PAGE plates, the Quantity One software (version 4.6.2) was utilized for analyzing the relative mobility (Rf), band intensity (Int.), and band percent (B%) of the electrophoretically separated bands. The equation proposed by Nei and Li [61] was used for calculating percentages of the similarity index (SI%).

### Molecular assay

The relative expression of the apoptotic genes (Bcl2-associated X protein (BAX), B-cell leukemia/lymphoma (Bcl2), tumor suppressor protein 53 (p^53^), and caspase-3) was assayed in both liver and brain tissues. The total RNA was isolated by homogenizing the tissue samples in TRIzol® Reagent (Invitrogen, USA). Integrity of the isolated RNA was assured using ethidium bromide stain analysis of 28S and 18S bands. The DNA residues were digested by treating total RNA with RQ1 RNase-free DNase (1 U) then re-suspended in DEPC-treated water. The RevertAid™ First Strand cDNA Synthesis Kit (MBI Fermentas, Germany) used during the reverse transcription process for converting complete poly(A)^+^ RNA (isolated from tissue samples) into cDNA. StepOne™ Real-Time PCR System (Thermo Fisher Scientific, Waltham, MA, USA) was used for determining the number of the copies in the tissue samples. The reaction program was allocated to three steps. The first step was at 95.0 °C for 3 min. The second step consisted of 40 cycles in which each cycle was divided into three steps: (a) at 95.0 °C for 15 s, (b) at 55.0 °C for 30 s, and (c) at 72.0 °C for 30 s. The third step consisted of 71 cycles which started at 60.0 °C and then increased at about 0.5 °C every 10 s up to 95.0 °C. The primers with the sequences suitable to the targeted genes are compiled in Supplementary Table 1. At the end of each qPCR, quality of the specific primers was checked at 95.0 °C using a melting curve analysis. Levels of the targeted genes were quantified in triplicate using the 2^−ΔΔCT^ method as described by Wang et al. [62].

### Statistical analysis

**Fig. 1:**
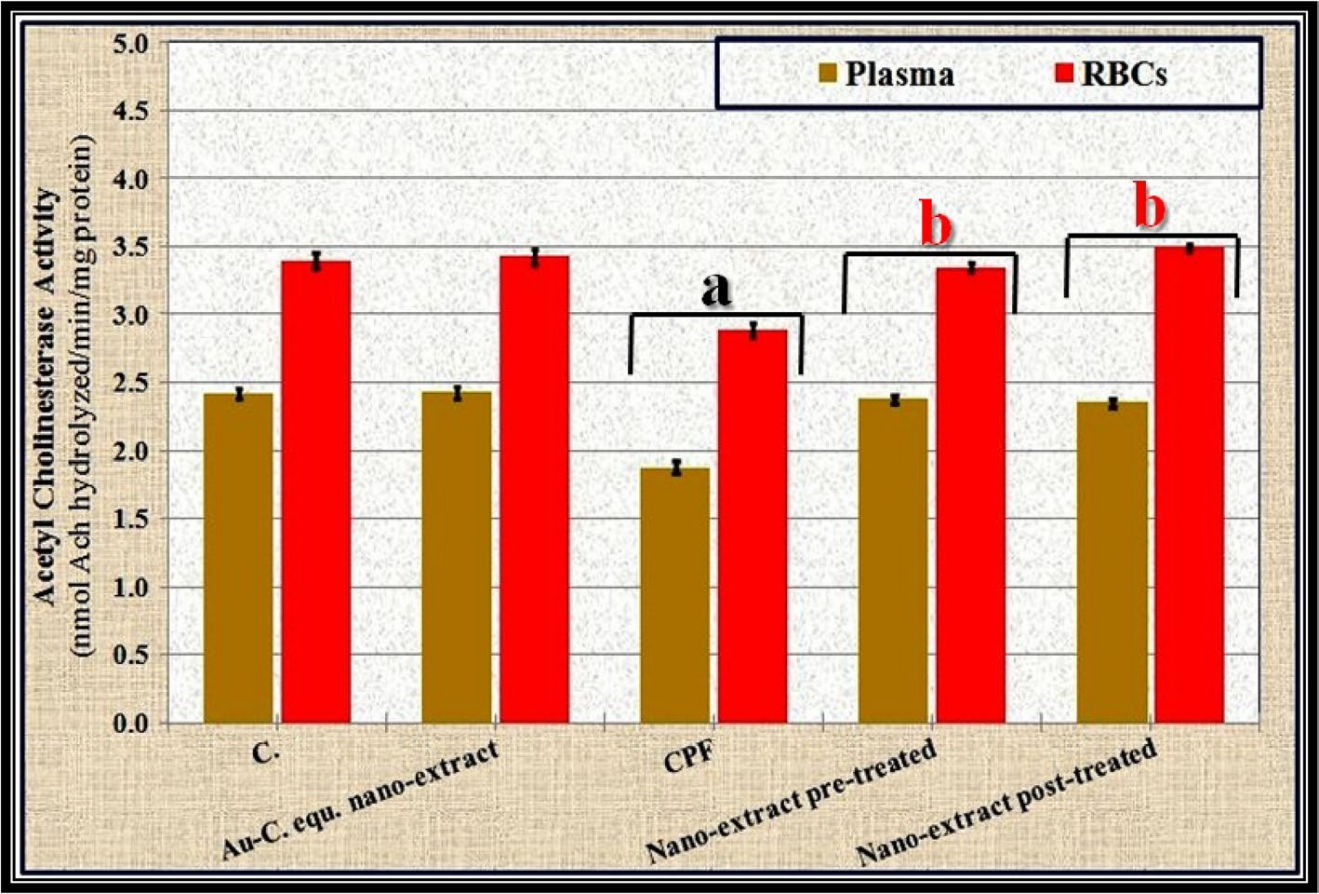
Effect of *C. equisetifolia* extract incorporated with gold nanoparticles (Au-NPs) against toxicity induced by chlorpyrifos (CPF) on the activity of acetylcholinesterase (AChE) in the blood of rats. Data were calculated from five replicates and expressed as mean ± SE, **a** significant versus control group and **b** significant versus toxic (CPF) group at *P* ≤ 0.05

**Fig. 2:**
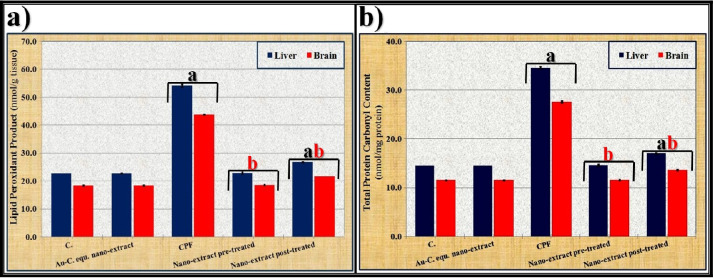
Effect of *C. equisetifolia* extract incorporated with gold nanoparticles (Au-NPs) against toxicity induced by chlorpyrifos (CPF) on **a)** lipid peroxidation product and **b)** total protein carbonyl content in liver and brain tissues of rats. Data were calculated from five replicates and expressed as mean ± SE, **a** significant versus control group and **b** significant versus toxic (CPF) group at *P* ≤ 0.05

Data were statistically evaluated using one-way analysis of variance (ANOVA) and are presented in Tables 1 and 2 and Figs. 1 and 2 as mean ± standard error (SE). The difference was considered significant when the *P* value was less than 0.05.

**Table 1:** Effect of *C. equisetifolia* extract incorporated with gold nanoparticles (Au-NPs) against toxicity induced by chlorpyrifos (CPF) on markers of oxidative stress in liver and brain tissues of rats

	**C**	**Au-*C. equisetifolia* nano-extract**	**CPF**	**CPF + Au-*C. equisetifolia* nano-extract**	
				**Pre-treated**	**Post-treated**
**Liver**					
**TAC (µmol/g)**	30.79 ± 0.05	30.71 ± 0.09	12.90 ± 0.10^a^	30.55 ± 0.41^b^	26.03 ± 0.08^ab^
**GSH (mg/g tissue)**	14.63 ± 0.04	14.59 ± 0.02	6.13 ± 0.05^a^	14.51 ± 0.17^b^	12.40 ± 0.07^ab^
**SOD (IU/g tissue)**	18.09 ± 0.01	18.11 ± 0.01	7.58 ± 0.05^a^	17.95 ± 0.23^b^	15.33 ± 0.09^ab^
**CAT (IU/g tissue)**	9.20 ± 0.01	9.20 ± 0.01	3.85 ± 0.02^a^	9.12 ± 0.12^b^	7.80 ± 0.04^ab^
**GPx (IU/g tissue)**	8.40 ± 0.02	8.32 ± 0.01	3.49 ± 0.02^a^	8.27 ± 0.10^b^	7.07 ± 0.03^ab^
**Brain**					
**TAC (µmol/g)**	27.18 ± 0.11	27.20 ± 0.12	11.38 ± 0.08^a^	26.97 ± 0.34^b^	23.04 ± 0.15^ab^
**GSH (mg/g tissue)**	8.75 ± 0.02	8.73 ± 0.02	3.67 ± 0.02^a^	8.68 ± 0.11^b^	7.40 ± 0.04^ab^
**SOD (IU/g tissue)**	11.39 ± 0.01	11.38 ± 0.01	4.77 ± 0.03^a^	11.30 ± 0.14^b^	9.64 ± 0.05^ab^
**CAT (IU/g tissue)**	6.69 ± 0.01	6.69 ± 0.01	2.80 ± 0.02^a^	6.63 ± 0.09^b^	5.66 ± 0.03^ab^
**GPx (IU/g tissue)**	5.28 ± 0.01	5.29 ± 0.01	2.21 ± 0.01^a^	5.24 ± 0.06^b^	4.47 ± 0.02^ab^

Data were calculated from five replicates and expressed as mean ± SE

^a^Significant versus control group at *P* ≤ 0.05

^b^Significant versus toxic (CPF) group at *P* ≤ 0.05

**Table 2:** Effect of *C. equisetifolia* extract incorporated with gold nanoparticles (Au-NPs) against toxicity induced by chlorpyrifos (CPF) on the biochemical measurements related to the integrity of liver and brain tissues of rats

	**C**	**Au-*C. equisetifolia* nano-extract**	**CPF**	**CPF + Au-*C. equisetifolia* nano-extract**	
				**Pre-treated**	**Post-treated**
**Liver**					
**TNF-α (pg/g tissue)**	344.00 ± 0.95	343.20 ± 0.58	726.40 ± 1.50^a^	427.40 ± 1.03^ab^	591.60 ± 1.17^ab^
**IL-6 (pg/g tissue)**	415.40 ± 1.29	415.60 ± 1.50	877.17 ± 1.67^a^	516.11 ± 1.66^ab^	714.40 ± 2.12^ab^
**Hydroxyproline (kg/mg tissue)**	0.32 ± 0.00	0.33 ± 0.01	0.68 ± 0.01^a^	0.40 ± 0.01^ab^	0.55 ± 0.01^ab^
**AChE (ng/g tissue)**	0.50 ± 0.02	0.48 ± 0.02	0.31 ± 0.02^a^	0.30 ± 0.02^a^	0.29 ± 0.02^a^
**Brain**					
**TNF-α (pg/g tissue)**	133.00 ± 0.55	134.00 ± 0.63	280.86 ± 1.65^a^	165.26 ± 1.27^ab^	228.73 ± 1.06^ab^
**IL-6 (pg/g tissue)**	224.80 ± 1.32	225.60 ± 0.60	474.68 ± 2.21^a^	279.30 ± 1.73^ab^	386.61 ± 2.37^ab^
**β-Amyloid (pg/g tissue)**	6.19 ± 0.01	6.19 ± 0.01	13.08 ± 0.04^a^	7.69 ± 0.03^ab^	10.65 ± 0.01^ab^
**AChE (ng/g tissue)**	9.37 ± 0.45	10.93 ± 0.79	7.63 ± 0.46^a^	10.76 ± 0.44^b^	9.60 ± 0.35^b^

Data were calculated from five replicates and expressed as mean ± SE

^a^Significant versus control group at *P* ≤ 0.05

^b^Significant versus toxic (CPF) group at *P* ≤ 0.05

## Results

There were various adverse effects occurred at the hematological, biochemical, and molecular levels following CPF injection.

### Body weight gain and relative organ weight assays

During the experimentation period, toxic symptoms were noticed in any of the animals. There were no changes in the nature of the treated rats’ stool, urine, and eye color. Prior to the beginning of the experiment (on day 0), the animals were weighed, and then body weight gains were calculated after 15 days and at the end of the experiment (after 30 days) of recovery period. As illustrated in Supplementary Fig. 1a, CPF injection decreased body weight gain significantly (*P* ≤ 0.05) throughout the duration of the study. Compared to the CPF-injected group, the body weight gain increased in the nano-extract pre- and post-treated groups significantly (*P* ≤ 0.05) but it could not be returned to the normal weights as control rats. Although the Au-*C. equisetifolia* nano-extract alone decreased the body weight, it increased gain of the body weight when administered in combination with CPF especially in the pre-treated group. As shown in Supplementary Fig. 1b, the absolute and relative weights of liver and brain tissues increased in the CPF-injected group significantly (*P* ≤ 0.05) compared to the control group. The nano-extract reduced the relative weights of these tissues significantly (*P* ≤ 0.05) in the nano-extract pre- and post-treated groups compared to the CPF-injected group. Compared to the CPF-injected group, administration of nano-extract decreased the relative weights of liver and brain tissues significantly (*P* ≤ 0.05) and it normalized their values to the nearest relative weights in control rats at the end of the experiment.

### Hematological measurements

During the present study, it was found that the hematological and biochemical measurements are considered as the preliminary mirror to the toxicity induced by these OP compounds. As shown in Supplementary Table 2, the most hematological parameters including indices of red blood cells (RBCs, HB, HCT, MCV, RDW, MPV, and PLT) and WBCs and their differential parameters decreased in the CPF-intoxicated group significantly (*P* ≤ 0.05) compared to the control group. No significant changes were noticed in the MCH and MCHC levels. Compared to the CPF-injected group, the gold nano-extract normalized the levels of these measurements in all treated groups.

### Biochemical measurements

As depicted in Supplementary Table 3, it was shown that activities of liver enzymes (ALT, AST, ALP, and GGT) elevated significantly (*P* ≤ 0.05) in sera of CPF-injected group compared to those of control group. In regard to renal functions, CPF injection caused significant (*P* ≤ 0.05) elevation in levels of urea, creatinine, and uric acid with lowering levels of T. protein and albumin. Moreover, CPF affected heart functions by increasing activities of heart enzymes (CK and LDH) significantly (*P* ≤ 0.05) with elevating levels of lipid measurements (TC, TGs, and LDL-c) compared to the control group. The Au-*C. equisetifolia* nano-extract restored levels of the biochemical parameters to normalcy in all nano-extract-treated groups. As illustrated in Fig. 1, activity of AChE enzyme decreased in plasma and RBCs significantly (*P* ≤ 0.05) in the CPF-intoxicated group compared to the control group. Compared to the CPF-injected group, the nano-extract restored the AChE activity to normal value in both the pre- and post-treated groups.

### Markers of oxidative stress

It was noticed that levels of non-enzymatic (TAC and GSH) and enzymatic (SOD, CAT, and GPx) antioxidants decreased as depicted in Table 1 with increasing concentrations of LPO and TPC content significantly (*P* ≤ 0.05) in both liver and brain tissues of CPF-injected group compared to those of the control group (Fig. 2). Administration of Au-*C. equisetifolia* nano-extract enhanced the measurements of the antioxidant system significantly (*P* ≤ 0.05) with declining concentrations of LPO and TPC content in both the pre- and post-treated groups compared to the CPF-injected group. It normalized levels of these measurements in the nano-extract pre-treated group.

### Inflammatory and fibrotic markers

Markers of the inflammatory (TNF-α and IL-6) and fibrotic (hydroxyproline) reactions in addition to AChE activity and level of Aβ are considered as indicators for integrity of liver and brain tissues. Data presented in Table 2 showed that levels of these markers increased in liver tissues of CPF-injected group significantly (*P* ≤ 0.05) with reducing AChE activity. The Au-*C. equisetifolia* nano-extract lowered the levels of these biomarkers in both the pre- and post-treated groups significantly (*P* ≤ 0.05) compared to the CPF-injected group, but it could not restore their levels to normalcy. In regard to AChE activity, Au-*C. equisetifolia* nano-extract caused no ameliorative effect in both the pre- and post-treated groups compared to the CPF-injected group. In brain tissue, the intoxication with CPF increased levels of inflammatory markers (TNF-α and IL-6) and Aβ with reducing AChE activity significantly (*P* ≤ 0.05) compared to the control group. The gold nano-extract reduced the levels of inflammatory markers and Aβ content with elevating AChE activity significantly (*P* ≤ 0.05) compared to the CPF-injected group. It showed a higher ameliorative effect in the pre-treated group.

### Histopathological examination

**Fig. 3:**
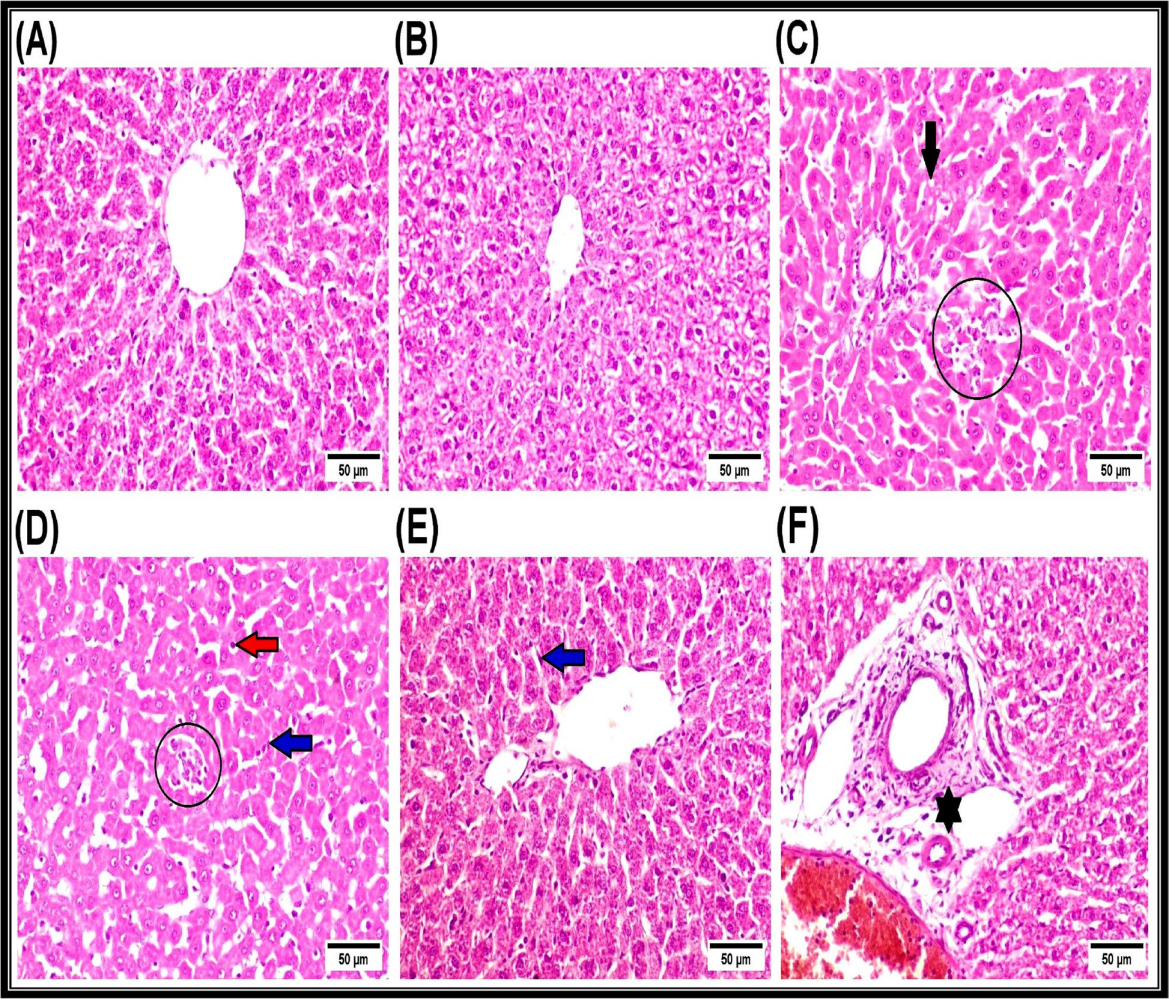
Representative photomicrographs of H&E-stained liver sections of rats (scale bar = 50 µm). **A** Normal control group showing the normal histoarchitecture of hepatic tissue. **B** Au-*C. equisetifolia* nano-extract-treated group showing no histopathological alterations. **C** and **D **Toxic (CPF-intoxicated) group showing hepatocellular vacuolar degeneration (black arrow), focal hepatocellular necrosis associated with infiltration of inflammatory cells (black circle), proliferation of Kupffer cells (blue arrow), and apoptosis of sporadic hepatocytes (red arrow). **E** Au-*C. equisetifolia* nano-extract pre-treated group showing proliferation of Kupffer cells (blue arrow). **F** Au-*C. equisetifolia* nano-extract post-treated group showing edema in the portal triad (asterisk)

In liver sections of control rats, light microscopic examination exhibited normal histological architecture of hepatic parenchyma (Fig. 3A). Likewise, no histopathological alterations were seen in the liver of rats treated with Au-*C. equisetifolia* nano-extract (Fig. 3B). On contrary, liver of rats intoxicated with CPF showed prominent histopathological damage characterized by infiltration of inflammatory cells and focal hepatocellular necrosis associated with hepatocellular vacuolar degeneration (Fig. 3C), proliferation of Kupffer cells, apoptosis of sporadic hepatocytes (Fig. 3D), and vesicular hepatocytic nuclei. Meanwhile, the hepatic histopathological alterations were ameliorated markedly in the liver of nano-extract pre-treated rats. In addition, the examined sections revealed proliferation of Kupffer cells (Fig. 3E). On the other hand, proliferation of Kupffer cells, necrosis of sparsely hepatocytes, and edema in the portal triad were observed in the liver of nano-extract post-treated rats (Fig. 3F). Data presented in Supplementary Table 4 summarizes scores of the histopathological lesions in livers of different experimental groups, and it was noticed that the highest deleterious effect exists in the liver of CPF-intoxicated rats. The highest ameliorative efficacy exists in the nano-extract pre-treated group.

**Fig. 4:**
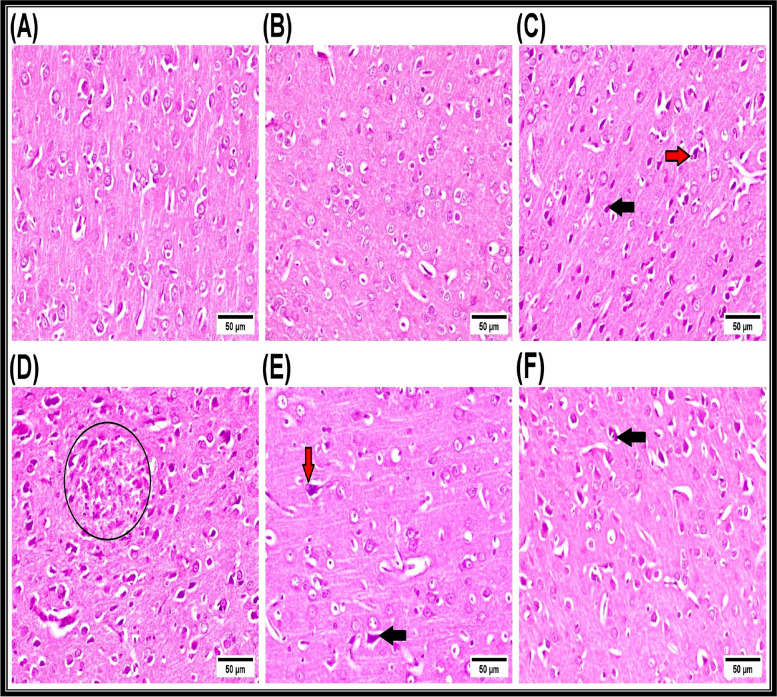
Representative photomicrographs of H&E-stained cerebral cortex of rats (scale bar = 50 µm). **A** Control and **B** Au-*C. equisetifolia* nano-extract-treated groups showing the normal histological architecture with intact neurons. **C** and **D** Toxic (CPF-intoxicated) group showing dark necrotic neurons (black arrow), neuronophagia (red arrow), and focal cerebral necrosis associated with proliferation of glial cells (black circle). **E** Au-*C. equisetifolia* nano-extract pre-treated group showing necrosis of sporadic neurons (black arrow) and neuronophagia (red arrow). **F** Au-*C. equisetifolia* nano-extract post-treated group showing necrosis of some neurons (black arrow)

Concerning brain tissue, the cerebral cortex of control rats as well as nano-extract-treated rats exists with normal histological architecture with intact neurons (Fig. 4A, B). Contrariwise, the remarkable neuropathic lesions investigated in CPF-intoxicated rats were characterized by marked necrosis of neurons, neuronophagia (Fig. 4C), perivascular cuffing with glial cells, and focal cerebral necrosis associated with proliferation of glial cells (Fig. 4D). Otherwise, the cerebral cortex of rats in the nano-extract pre-treated group revealed marked amelioration with regressed histopathological lesions. The examined sections showed necrosis of neuronophagia and sporadic neurons (Fig. 4E). Furthermore, the cerebral cortex of rats in the nano-extract post-treated group showed necrosis of some neurons (Fig. 4F), neuronophagia, and mild proliferation of glial cells. Data depicted in Supplementary Table 5 summarizes scores of the histopathological lesions in brain tissue in rats of different experimental groups, and it was observed that intoxication of CPF caused a statistically significant (*P* ≤ 0.05) adverse effect. The highest ameliorative effect of nano-extract exists in the pre-treated group.

### Electrophoretic assays

#### Electrophoretic protein pattern

**Fig. 5:**
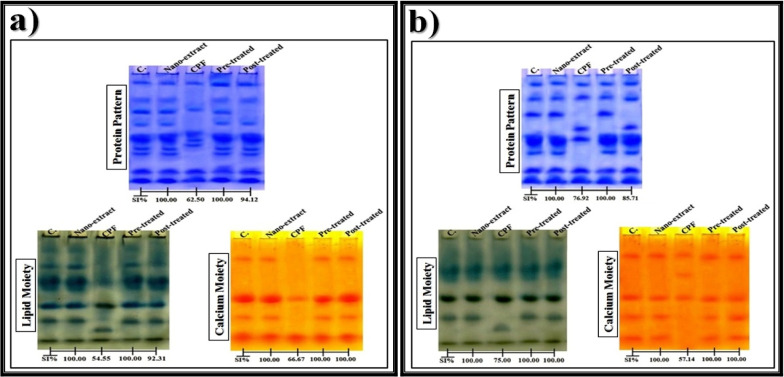
Native electrophoretic protein patterns showing an effect of *C. equisetifolia* extract incorporated with gold nanoparticles (Au-NPs) against the physiological alterations induced by chlorpyrifos (CPF) in **a**) liver and **b**) brain tissues of rats

In the liver of control rats (Fig. 5a), the native protein pattern was electrophoretically represented by nine bands (Rf values 0.08, 0.22, 0.32, 0.42, 0.54, 0.62, 0.66, 0.80, and 0.88; Int. 150.18, 151.31, 170.80, 154.86, 187.91, 189.35, 154.95, 171.49, and 173.10; B% 9.99, 10.06, 11.36, 10.30, 12.49, 12.59, 10.30, 11.40, and 11.51, respectively). Four common bands (at Rf values 0.08, 0.54, 0.80, and 0.88) were identified. In the CPF-injected group, the physiological alterations were represented by hiding four normal protein bands with the appearance of two abnormal (characteristic) ones identified at Rf values 0.50 and 0.59 (Int. 174.82 and 158.12; B% 14.55 and 13.16, respectively). Compared to the control group, SI values (SI = 62.50%) decreased in the liver of CPF-injected group. The protein pattern was improved by the pre-treatment with nano-extract through restoring four normal ones (Rf values 0.22, 0.41, 0.62, and 0.66; Int. 149.40, 157.49, 189.30, and 160.04; B% 9.75, 10.28, 12.36, and 10.45, respectively) with hiding the abnormal (characteristic) bands. The post-treatment with nano-extract showed a slight ameliorative effect by restoring four normal bands identified at Rf values 0.23, 0.54, 0.63, and 0.67 (Int. 149.02, 198.87, 189.52, and 171.96; B% 11.11, 14.93, 14.23, and 12.91, respectively) associated with hiding the characteristic bands in addition to hiding another normal one. Therefore, the SI values increased in the nano-extract pre- (SI = 100.00%) and post-treated (SI = 94.12%) groups.

In the brain of control rats (Fig. 5b), it was noticed that native protein pattern was represented by seven bands (Rf values 0.08, 0.20, 0.33, 0.56, 0.66, 0.80, and 0.88; Int. 142.86, 149.92, 140.17, 174.08, 124.96, 151.88, and 116.54; B% 14.28, 14.99, 14.01, 17.40, 12.49, 15.18, and 11.65, respectively). Five common bands (at Rf values 0.08, 0.20, 0.56, 0.80, and 0.88) were identified. No characteristic bands were detected. Injection of CPF caused physiological alterations represented by hiding two normal protein bands with existence of one abnormal band (Rf 0.45; Int. 158.24; B% 18.25). Compared to the control group, SI values (SI = 76.92%) decreased in the brain of CPF-injected group. The pre-treatment with nano-extract ameliorated the protein pattern by restoring two normal bands (Rf values 0.33 and 0.65; Int. 138.75 and 142.91; B% 13.57 and 13.98, respectively) with hiding the abnormal one. The post-treatment with gold nano-extract showed a lower ameliorative effect than the pre-treated group by restoring only one normal band (Rf 0.65; Int. 137.73; B% 12.82) without hiding the abnormal one that exists at Rf 0.44 (Int. 149.51; B% 13.92). Compared to the control group, the SI values increased in the nano-extract pre- (SI = 100.00%) and post-treated (SI = 85.71%) groups.

#### Electrophoretic lipid moiety of native protein pattern

In the liver of control rats (Fig. 5a), the lipid moiety of native protein pattern was represented by seven bands (Rf values 0.12, 0.27, 0.40, 0.47, 0.62, 0.76, and 0.93; Int. 195.42, 204.73, 221.62, 186.95, 188.37, 167.73, and 163.19; B% 14.72, 15.42, 16.69, 14.08, 14.18, 12.63, and 12.29, respectively). Three common bands (at Rf values 0.40, 0.62, and 0.93) were identified. In the CPF-injected group, the physiological alterations were represented by hiding four normal protein bands with existence of one abnormal (characteristic) band (Rf 0.84; Int. 173.35; B% 23.66). Compared to control, the SI values (SI = 54.55%) decreased in the liver of CPF-injected group. The pre-treatment with nano-extract improved this pattern by restoring four normal bands (Rf values 0.11, 0.26, 0.47, and 0.76; Int. 191.07, 203.21, 185.15, and 173.59; B% 14.40, 15.314, 13.95, and 13.08, respectively) with hiding the abnormal (characteristic) one. The post-treatment with gold nano-extract showed a slight ameliorative effect by restoring only three normal bands (Rf values 0.40, 0.61, and 0.92; Int. 208.56, 194.40, and 174.16; B% 18.36, 17.11, and 15.33, respectively) with hiding the characteristic one. The SI values increased in the nano-extract pre- (SI = 100.00%) and post-treated (SI = 92.31%) groups compared to the control group.

In the brain of control rats (Fig. 5b), it was observed that the lipid moiety of native protein pattern was represented by four bands (Rf values 0.26, 0.50, 0.66, and 0.86; Int. 207.38, 185.82, 162.99, and 156.48; B% 29.10, 26.07, 22.87, and 21.96, respectively). Three common bands (at Rf values 0.26, 0.50, and 0.86) were identified. The CPF caused alterations in this native pattern represented by hiding one normal band with existence of one abnormal (characteristic) band (Rf 0.75; Int. 157.04; B% 22.06). Therefore, SI values (SI = 75.00%) decreased in the liver of CPF-injected group compared to the control group. The nano-extract showed the same ameliorative effect at all therapeutic modes by hiding the abnormal band with restoring the normal band in the nano-extract pre- (Rf 0.65; Int. 167.63; B% 22.86) and post-treated (Rf 0.65; Int. 161.12; B% 22.05) groups. The nano-extract pre- and post-treated groups became physiologically similar to the control group (SI = 100.00%).

#### Electrophoretic calcium moiety of native protein pattern

In the liver of control rats (Fig. 5a), the calcium moiety of native protein pattern was represented by four bands (Rf values 0.18, 0.53, 0.68, and 0.85; Int. 138.68, 168.60, 138.76, and 177.67; B% 22.23, 27.03, 22.25, and 28.49, respectively). Two common bands (at Rf values 0.53 and 0.85) were identified. No characteristic bands were noticed. In the CPF-injected group, the physiological abnormalities in this native pattern were represented only by hiding two normal bands. Compared to the control, the SI values (SI = 66.67%) decreased in the liver of CPF-injected group. The gold nano-extract showed the same ameliorative effect at all therapeutic modes by restoring the normal bands in the pre- (Rf values 0.18 and 0.69; Int. 145.21 and 138.89; B% 24.11 and 23.06, respectively) and post-treated (Rf values 0.18 and 0.68; Int. 139.79 and 139.37; B% 22.57 and 22.50, respectively) groups. Therefore, the nano-extract pre- and post-treated groups became physiologically identical to the control group (SI = 100.00%).

In the brain of control rats (Fig. 5b), it was noticed that the calcium moiety of native protein pattern was represented by four bands (Rf values 0.20, 0.54, 0.73, and 0.91; Int. 157.22, 154.39, 134.61, and 133.50; B% 27.12, 26.63, 23.22, and 23.03, respectively). There at two common bands (Rf values 0.20 and 0.73). The CPF caused abnormalities in this native pattern represented by hiding two normal bands with existence of one abnormal (characteristic) band (Rf 0.36; Int. 127.28; B% 31.54). Therefore, SI values (SI = 57.14%) decreased in the CPF-injected group compared to the control group. Administration of gold nano-extract showed the same ameliorative effect by hiding the characteristic band with restoring the normal bands in the pre- (Rf values 0.73 and 0.91; Int. 149.96 and 141.15; B% 26.55 and 24.99, respectively) and post-treated (Rf values 0.73 and 0.90; Int. 146.91 and 129.15; B% 25.72 and 22.61, respectively) groups. Therefore, the nano-extract pre- and post-treated groups became physiologically qualitatively identical to the control group (SI = 100.00%).

#### Electrophoretic CAT pattern

**Fig. 6:**
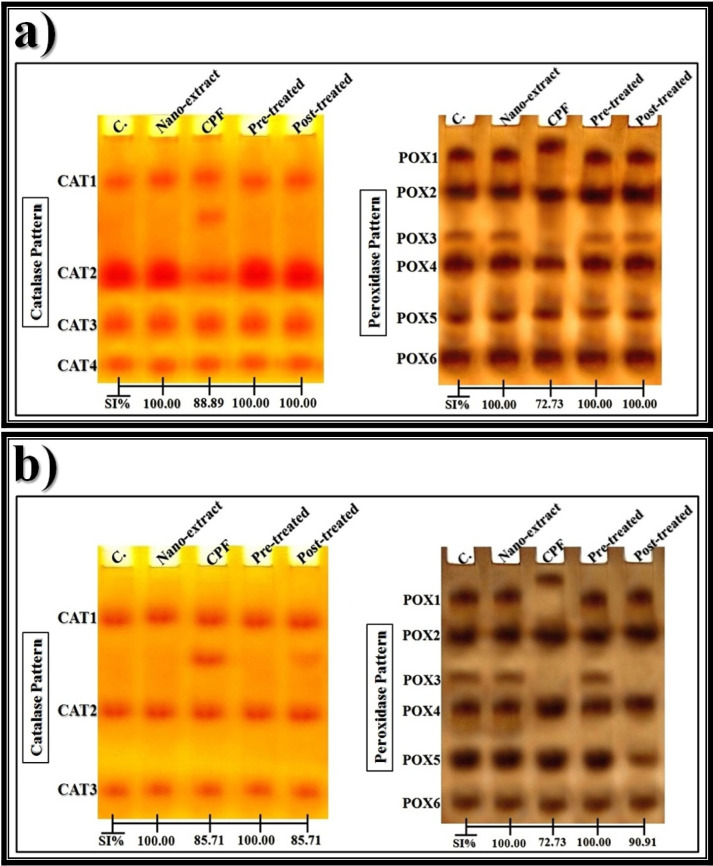
Native electrophoretic isoenzyme patterns showing an effect of *C. equisetifolia* extract incorporated with gold nanoparticles (Au-NPs) against the physiological alterations induced in catalase (CAT) and peroxidase (POX) by chlorpyrifos (CPF) in **a**) liver and **b**) brain tissues of rats

As illustrated in Fig. 6a, the CAT isoenzyme pattern in the liver of control rats was represented by four types (Rf values 0.18, 0.54, 0.74, and 0.90; Int. 146.68, 199.27, 122.55, and 96.58; B% 25.96, 35.26, 21.69, and 17.09, respectively). All CAT types are considered as common bands. In the CPF-injected group, the qualitative abnormalities in the CAT isoenzyme pattern were represented by appearance of one abnormal (characteristic) band (Rf 0.32; Int. 135.44; B% 20.09) without hiding normal types. Compared to the control group, the lowest SI value (SI = 88.89%) was recorded in the CPF-injected group. The nano-extract showed the same ameliorative effect at all therapeutic modes by hiding the characteristic band. The CAT isoenzyme pattern in both nano-extract pre- and post-treated groups was completely similar to that in the control group (SI = 100.00%).

In the brain of control rats (Fig. 6b), it was found that the CAT isoenzyme pattern was represented by three types (Rf values 0.20, 0.56, and 0.88; Int. 134.79, 133.57, and 121.64; B% 34.56, 34.25, and 31.19, respectively). The three CAT types are considered as common bands. The CPF caused physiological abnormalities in the CAT isoenzyme pattern represented by appearance of one abnormal band (Rf 0.36; Int. 131.63; B% 26.34) without hiding one or more of normal types. Therefore, SI values (SI = 85.71%) decreased in the CPF-injected group compared to the control group. The pre-treatment with nano-extract showed a higher ameliorative effect detected qualitatively in the CAT isoenzyme pattern by hiding the abnormal band completely. Therefore, this isoenzyme pattern in the nano-extract pre-treated group became physiologically identical to that in the control group (SI = 100.00%). In the nano-extract post-treated group, the nano-extract could not prevent the qualitative alterations completely but it decreased the intensity of the abnormal band (Rf 0.37 and Int. 112.06) compared to that in the CPF-injected group (Rf 0.36 and Int. 131.63). The SI value in the nano-extract post-treated group was equal to that in the CPF-injected group (SI = 85.71%) compared to the control group.

#### Electrophoretic POX pattern

As shown in Fig. 6a, the POX isoenzyme pattern in the liver of control rats was represented by six types (Rf values 0.11, 0.25, 0.42, 0.55, 0.75, and 0.91; Int. 179.03, 194.01, 162.55, 181.09, 210.09, and 200.59; B% 15.88, 17.21, 14.42, 16.06, 18.64, and 17.79, respectively). Four POX types (POX2, POX4, POX5, and POX6) were regarded as common bands. The CPF caused qualitative abnormalities represented by hiding the POX1 type with existence of one abnormal (characteristic) band (Rf 0.07; Int. 183.95; B% 18.90). Compared to the control group, the CPF-injected group exists with the lowest SI value (SI = 72.73%). The nano-extract showed the same ameliorative effect at all therapeutic modes by hiding the characteristic band. In the nano-extract pre- and post-treated groups, the POX isoenzyme pattern was physiologically identical to that in the control group (SI = 100.00%).

In the brain of control rats (Fig. 6b), it was found that the POX isoenzyme pattern was represented by six types (Rf values 0.12, 0.27, 0.42, 0.53, 0.73, and 0.89; Int. 192.96, 184.91, 158.05, 195.21, 186.79, and 175.60; B% 17.65, 16.91, 14.45, 17.85, 17.08, and 16.09, respectively). The four POX types (POX2, POX4, POX5, and POX6) that were identified at Rf values 0.27, 0.53, 0.73, and 0.89 are considered as common bands. The CPF caused physiological abnormalities in the POX isoenzyme pattern represented by hiding the POX1 and POX3 types with existence of one abnormal (characteristic) band (Rf 0.06; Int. 164.58; B% 17.74). Compared to the control group, the CPF-injected group exists with the lowest SI value (SI = 72.73%). The nano-extract showed a higher ameliorative effect in the pre-treated group by hiding the abnormal band with restoring the two normal POX types (POX1 and POX3 types) identified at Rf values 0.12 and 0.42 (Int. 193.25 and 158.51; B% 18.09 and 14.83, respectively). The POX isoenzyme pattern in the nano-extract pre-treated group was physiologically identical to that in the control group (SI = 100.00%). In the post-treated group, the nano-extract showed a slight ameliorative effect by hiding the abnormal band with restoring one normal type (POX1) (Rf 0.11; Int. 187.29; B% 21.05). Therefore, the POX isoenzyme pattern in the nano-extract post-treated group was identical to that in the control group by 90.91%.

#### Electrophoretic α-Amy pattern

**Fig. 7:**
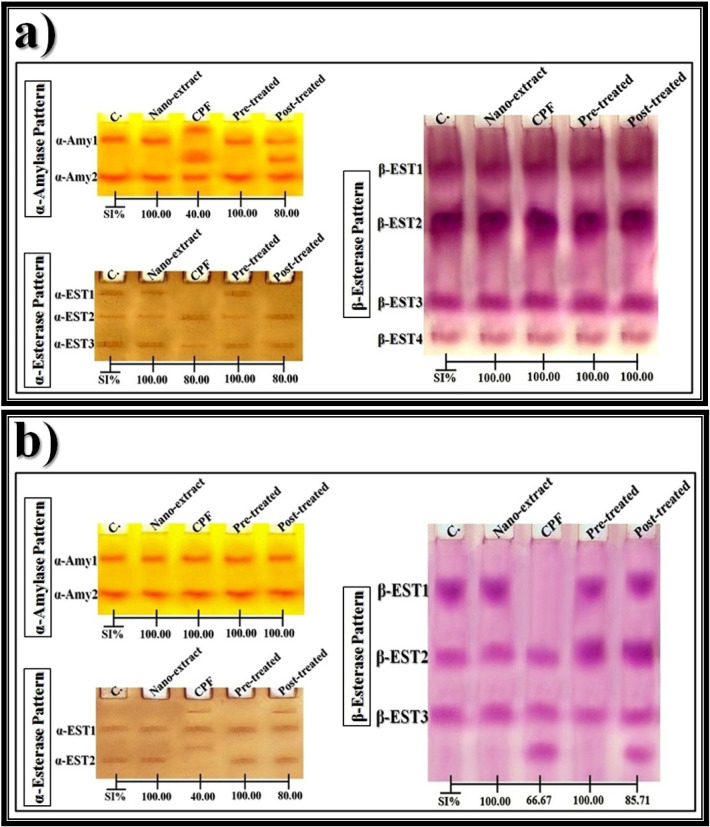
Native electrophoretic isoenzymes patterns showing an effect of *C. equisetifolia* extract incorporated with gold nanoparticles (Au-NPs) against the physiological alterations induced in α-amylase (α-Amy), α-esterase (α-EST), and β-esterase (β-EST) by chlorpyrifos (CPF) in **a**) liver and **b**) brain tissues of rats

In the liver of control rats (Fig. 7a), the electrophoretic α-Amy isoenzyme pattern was represented by two types (Rf values 0.24 and 0.70; Int. 95.47 and 105.18; B% 47.58 and 52.42, respectively). The second α-Amy type (α-Amy2) identified at Rf 0.70 is considered as a common band. The qualitative abnormalities in the α-Amy isoenzyme pattern were represented in the CPF-injected group by hiding the α-Amy1 type with appearance of two abnormal bands (Rf values 0.10 and 0.45; Int. 98.19 and 122.22; B% 30.28 and 37.69, respectively). The first abnormal band (Rf 0.10) is considered as a characteristic band. Compared to the control group, the CPF-injected group exists with the lowest SI value (SI = 40.00%). The nano-extract ameliorated the α-Amy isoenzyme pattern in the pre-treated group by hiding the abnormal bands with restoring the normal α-Amy type (α-Amy1) (Rf 0.26; Int. 98.97; B% 46.59). Therefore, the α-Amy isoenzyme pattern in this group was physiologically identical to that in the control group (SI = 100.00%). In the post-treated group, the nano-extract showed a slight ameliorative effect by restoring the normal α-Amy type (α-Amy1) identified at Rf 0.25 (Int. 86.01 and B% 29.00) with hiding only one abnormal band. Therefore, the α-Amy isoenzyme pattern in this group was identical to that in the control group by 80.00%.

In the brain of control rats (Fig. 7b), the electrophoretic α-Amy isoenzyme pattern was represented by two types (Rf values 0.29 and 0.69; Int. 97.80 and 101.18; B% 49.15 and 50.85, respectively). Both of the two α-Amy types are considered as common bands. Injection of the CPF caused no qualitative or quantitative abnormalities in the α-Amy isoenzyme pattern. Administration of the nano-extract alone or in combination with CPF in the pre- and post-treated groups caused no differences from the control group in the α-Amy isoenzyme pattern. Therefore, the α-Amy isoenzyme pattern in all treated groups was physiologically identical to that in the control group (SI = 100.00%).

#### Electrophoretic EST pattern

In the liver of control rats (Fig. 7a), the electrophoretic α-EST isoenzyme pattern was represented by three types (Rf values 0.11, 0.34, and 0.64; Int. 159.05, 113.20, and 184.45; B% 34.83, 24.79, and 40.39, respectively). The two α-EST types (α-EST1 and α-EST2) identified at Rf values 0.34 and 0.64 are considered as common bands. The CPF caused qualitative abnormalities in the α-EST isoenzyme pattern represented by hiding the first α-EST (α-EST1) type without existence of abnormal bands. The CPF-injected group exists with the lowest SI value (SI = 80.00%) compared to the control group. Administration of the nano-extract showed a higher ameliorative effect in the pre-treated group by restoring the normal α-EST (α-EST1) type (Rf 0.09; Int. 154.57; B% 37.69). Therefore, the α-EST isoenzyme pattern in the nano-extract pre-treated group was physiologically identical to that in the control group (SI = 100.00%). In the post-treated group, the nano-extract could not prevent the physiological abnormalities. Therefore, the SI value in this group was equal to that in the CPF-injected group (SI = 80.00%) when compared to the control group.

In the brain of control rats (Fig. 7b), the α-EST isoenzyme pattern was represented by two types (Rf values 0.36 and 0.74; Int. 115.95 and 111.25; B% 51.03 and 48.97, respectively). The first α-EST type (α-EST1) identified at Rf 0.36 is considered as a common band. The CPF caused qualitative abnormalities in this isoenzyme pattern represented by hiding the α-EST2 type with existing two abnormal bands (Rf values 0.16 and 0.57; Int. 95.15 and 101.04; B% 30.59 and 32.48, respectively). The second abnormal band (Rf 0.57) is considered as a characteristic (unique) band. Compared to the control group, the CPF-injected group exists with the lowest SI value (SI = 40.00%). Administration of the nano-extract showed a higher ameliorative effect in the pre-treated group by restoring the normal α-EST (α-EST2) type identified at Rf 0.74 (Int. 98.91 and B% 47.46) with hiding the abnormal band. Therefore, the α-EST isoenzyme pattern in this group was qualitatively identical to that in the control group (SI = 100.00%). Regarding the post-treated group, the nano-extract showed a slight ameliorative effect by restoring α-EST2 type identified at Rf 0.73 (Int. 97.92 and B% 31.30) without hiding the abnormal band (Rf 0.16; Int. 94.36; B% 30.16). Therefore, the α-EST isoenzyme pattern in this group was qualitatively similar to that in the control group by 80.00%.

In the liver of control rats (Fig. 7a), the electrophoretic β-EST isoenzyme pattern was represented by four types (Rf values 0.17, 0.42, 0.74, and 0.89; Int. 189.18, 229.45, 149.51, and 96.66; B% 28.46, 34.51, 22.49, and 14.54, respectively). Both of the β-EST types are considered as common bands. Injection of the CPF caused no qualitative or quantitative abnormalities in the β-EST isoenzyme pattern. Administration of the nano-extract solely or in combination with CPF in the pre- and post-treated groups caused no differences from the control group in this isoenzyme pattern. Therefore, the β-EST isoenzyme pattern in all treated groups was physiologically identical to that in the control group (SI = 100.00%).

In the brain of control rats (Fig. 7b), the β-EST isoenzyme pattern was represented by three types (Rf values 0.20, 0.45, and 0.68; Int. 131.85, 110.46, and 103.21; B% 38.16, 31.97, and 29.87, respectively). The two β-EST types (β-EST2 and β-EST3) identified at Rf values 0.45 and 0.68 are considered as common bands. In all treated groups, no characteristic bands were identified. In the CPF-injected group, the qualitative alterations were represented by hiding the first β-EST (β-EST1) type with existing one abnormal band (Rf 0.82; Int. 108.56; B% 33.93). Compared to the control group, the CPF-injected group exists with the lowest SI value (SI = 66.67%). Administration of the nano-extract showed a higher ameliorative effect in the pre-treated group by restoring the normal β-EST (β-EST1) type (Rf 0.19; Int. 123.61; B% 34.92) with hiding the abnormal band. Therefore, the β-EST isoenzyme pattern in this group was qualitatively identical to that in the control group (SI = 100.00%). Regarding the post-treated group, the nano-extract ameliorated this pattern slightly by restoring the normal β-EST (β-EST1) type identified at Rf 0.19 (Int. 125.50 and B% 26.43) without hiding the abnormal band (Rf 0.83; Int. 104.26; B% 21.96). Therefore, the β-EST isoenzyme pattern in this group was qualitatively similar to that in the control group by 85.71%.

### Molecular assay

**Fig. 8:**
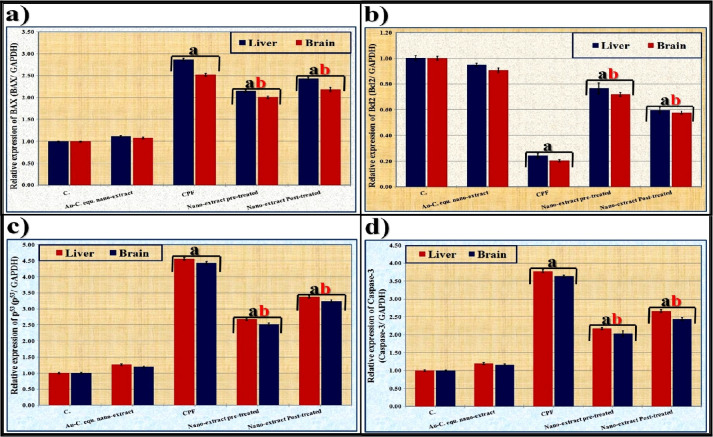
Effect of *C. equisetifolia* extract incorporated with gold nanoparticles (Au-NPs) against the alterations in the relative expression of apoptotic genes, **a**) BAX, **b**) Bcl2, and **c**) p^53^, and **d** caspase-3 genes, induced by chlorpyrifos (CPF) in liver and brain tissues of rats

It was carried out by detecting the expression of the apoptotic genes (BAX, Bcl2, p^53^, and caspase-3). As shown in Fig. 8, it was noticed that the relative expression of BAX, p^53^, and caspase-3 genes elevated significantly (*P* ≤ 0.05) with decreasing expression of Bcl2 gene in both liver and brain tissues of CPF-injected group compared to that of the control group. The nano-extract at all therapeutic modes resulted in decreasing the relative expression of BAX, p^53^, and caspase-3 genes significantly (*P* ≤ 0.05), as well as increasing the relative expression of Bcl2 gene in both liver and brain tissues compared to the CPF-injected group. Although nano-extract showed a higher ameliorative effect in the pre-treated group, it could not restore the relative expression levels of these genes to normal values.

## Discussion

The toxicity may occur mainly due to eating the meat of animals immersed in solutions of the OP compounds which are used widely in animal husbandry and agriculture [63]. Herbal products can be used as an effective strategy for enhancing the biological processes. Therefore, there is a considerable inclination to consume these natural remedies to diminish the toxicity brought on by the exposure to these toxicants [64]. The medicinal plants have gained prominence in nanobiotechnology due to availability of secondary metabolites that used during Au-NP biosynthesis as reducing agents [65]. The present work was designed to appraise the efficiency of the phytochemically synthesized Au-NPs against the toxicity induced by CPF. Hematological, biochemical, and molecular parameters as well as the histopathological examination were assessed to support the proposed hypothesis.

### Body weight gain and relative organ weight assays

Relative organ weight analysis is a crucial criterion for identifying substances that affect human health negatively [66]. It was demonstrated that the body weight gain decreased after CPF injection throughout the duration of the study and there is a decrease in the rate of the weight recovery. This was in accordance with Mossa and Abbassy [67] who postulated that the decline in body weight gains of CPF-intoxicated group might be related to decreasing consumption of the food that corresponded to lowering water and food intake compared to control rats and/or due to the direct effect of CPF on increasing degradation of lipids and proteins [68]. Also, it might refer to the ability of CPF to cause physical or reflex development [69].

The relative organ weights were used as the most sensitive indicator for revealing the deleterious effects of toxins [70]. The present study showed that the absolute and relative weights of liver and brain tissues increased significantly in rats injected with CPF, and this was supported with Pollakova et al. [71] who supposed that the relative liver weight increased in the CPF-injected group due to decreasing cell proliferation and damage of cell membrane in liver tissues. Regarding the brain tissue, the CPF caused an increase in the relative brain weight and this agreed with Akhtar et al. [72] who emphasized that the changes in brain weights might be related to reducing the body weight gain in addition to severity of neurotoxicity that occurs with histopathological changes. The Au-*C. equisetifolia* nano-extract alone caused a decrease in the body weight gains due to the presence of the Au-NPs that have the ability to accumulate rapidly in the abdominal fat tissue showing significant reduction in abdominal fat mass by improving lipid metabolic profile compared to control [73]. Treatment of the CPF-injected rats with nano-extract increased the body weight gain, and this agreed with Fereidounni and Dhawan [74] who suggested that the biosynthesized Au-NPs achieved a remarkable improvement in body weight due to increasing the phenolic groups in plant extract which prevented oxidative injury by increasing antioxidant action.

### Hematological measurements

It is well known that CPF affected spleen and bone marrow cells by accelerating apoptosis and inducing the DNA damage [75]. The current study showed that indices of red blood cells (RBCs, HB, HCT, MCV, RDW, MPV, and PLT) declined in the CPF-intoxicated group and this was in agreement with Deabes et al. [76] who demonstrated that the hematological measurements were altered in the CPF-injected group due to elevating products of the peroxidation reactions as a consequence of stimulating the oxidative stress. Moreover, the differential WBCs decreased in the CPF-injected group compared to the control group. This was coincided with Hashem and El-Sharkawy [77] who reported that CPF caused lymphopenic leucopenia due to the oxidative damage to the leukocytes. Treatment of the CPF-injected rats with Au-*C. equisetifolia* nano-extract normalized levels of these measurements in all groups treated with nano-extract, and this might be attributed to the existence of various polyphenolic compounds with high antioxidant activity and high ability to scavenge the ROS generated due to the exposure to CPF [76]. The nano-extract is rich in the active phytoconstituents which are able to improve absorption of iron by facilitating conversion of the iron from the ferrous (oxidized) state to the ferric (reduced) state in addition to ameliorating the integrity of the RBCs and WBCs compromised by the oxidative stress induced by CPF [78]. Moreover, they improved platelet count due to their ability to protect the platelet from oxidative damage, thus reducing the production of lipid peroxides within platelet membranes [79].

The AChE is considered as a standard biomarker for neurotoxic effects induced by OP compounds [80]. It is a membrane-bound enzyme that catalyzes the transformation of the acetylcholine (neurotransmitter) at the myoneural junction into choline and acetate [81]. The present study showed that CPF decreased the activity of the AChE enzyme in the plasma, and this was in accordance with Wielgomas and Krechniak [82] who suggested that decreasing the activity of this enzyme might be attributed to the binding of CPF to AChE, causing its inhibition irreversible at neuromuscular junctions and cholinergic synapses due to the oxidative damage to cholinergic cells by CPF. This consequently leads to accumulating acetylcholine in the synapses resulting in cholinergic toxicity due to inducing the hyperactivity in cholinergic pathways. As a result of blocking acetylcholine release from presynaptic membranes, the accumulated acetylcholine prevents conduction of the nerve impulse eventually [83]. Treatment of CPF-injected rats with nano-extract enhanced the activity of AChE enzyme in plasma and RBCs compared to that of the CPF-injected group due to the presence of the active (phenolic) ingredients that possess antioxidant properties [84]. Recently, Bekheit et al. [85] supported that efficiency of these phenolic compounds increased after incorporating Au-NPs.

### Biochemical measurements

Profiles of the blood chemistry were measured to assess the physiological and pathology state of vital organs in animals [86]. Data of our study showed a progressive increment in levels of the liver enzymes in sera of CPF-injected group, and this was supported by Heikal et al. [87], who proposed that the increase in activities of these enzymes is a result of functional damage in the liver (the final target site of CPF). Elevated levels of these hepatic enzymes could indicate dysfunction of the hepatocytes and disturbance in the biosynthesis of these enzymes, as well as changes in hepatocyte membrane permeability [88, 89]. The current study revealed that levels of urea, creatinine, and uric acid elevated in CPF-injected rats and this was in accordance with Kasteel et al. [90] who have proven that increasing these measurements might refer to degradation of both purines and pyrimidines and/or accelerating rate of protein catabolism rather than decreased urinary excretion of urea in addition to impairment of kidney function and reduction of the glomerular filtration rate that consequently lead to retention of urea in the blood. Also, concentrations of total protein and albumin decreased in CPF-injected rats due to increasing protein catabolism [91]. Intoxication of CPF increased levels of the heart enzymes (CK and LDH) and measurements of lipid profile. This agreed with Saoudi et al. [92] who documented that perturbations of the heart functions were attributable to an oxidative damage in CPF-injected rats. Moreover, the increase in levels of these measurements might be related to increasing permeability of cellular membranes [93]. The Au-*C. equisetifolia* nano-extract alleviated the hepatic, renal, and heart toxicity induced by CPF especially in the pre-treated group. The protective potential against liver damages might refer to the role of the phenolic compounds (the major phytoconstituents) in plant extracts that were responsible for an inhibitory effect on activities of transaminases [94]. Also, the biosynthesized Au-NPs showed a prominent decrease in activities of the liver enzymes due to stabilizing membrane of hepatocytes and preventing liberation of these enzymes [95]. Levels of the renal measurements restored to normalcy due to the presence of the bioactive components that exhibit various biological activities [96]. The presence of the Au-NPs enhanced the regenerative ability of the renal tubules (the primary site for Au-NP accumulation) and showed a pronounced effect on protein content due to its ability to normalize measurements of profile [97]. Levels of heart enzymes and lipid measurements were restored by nano-extract to normal values due to the hypolipidemic properties of the phytoconstituents which decrease levels of TC, TGs, and LDL [98]. Level of HDL increased by the nano-extract because of stimulating lecithin cholesterol acyltransferase involved in regulating serum lipids [99].

### Markers of oxidative stress

The changes in activities of antioxidant enzymes (SOD, CAT, and GPx) have been reported to be an indicator of oxidative stress induced by the exposure to OP compounds [100]. The current study showed that intoxication of CPF produced a significant decrease in markers of the antioxidant system in hepatic and brain tissues, and these findings are consistent with Ibrahim and Khwanes [101] who showed that CPF decreased activities of these enzymes due to overproduction of the ROS which could be linked to hepatocyte and brain lesions, as well as insufficient detoxification capacity of the CPF. The present study emphasized that the level of LPO elevated in hepatic and brain tissues in the CPF-injected group and this agreed with Kasteel et al. [90] who suggested that the LPO level increased due to disequilibrium in the biological system between pro-oxidants and antioxidants, resulting in overproduction of the ROS that consequently leads to damage of cellular biomacromolecules or cell death. Also, level of TPC content increased in those tissues after injecting CPF and this agreed with Ojha and Gupta [102] who suggested that the TPC content level increased in liver and brain (especially cerebrum and cerebellum regions) following the exposure to CPF due to the influx of ROS, which take protons from amino acids (methylene groups) to produce carbonyls; nucleophilic centers are often damaged; and sulfhydryl groups are oxidized, leading to the loss of protein activities. Furthermore, lowering levels of enzymatic (SOD, CAT, and GPx) and non-enzymatic (TAC and GSH) antioxidants might be associated with increasing peroxidation reaction products (LPO and TPC content) [103]. The Au-*C. equisetifolia* nano-extract alleviated the negative effects induced by CPF in the pre-treated group by restoring measurements of the antioxidant system in liver and brain tissues due to enhancing the phenolic compounds which are responsible for the antioxidant activity in nano-extract [104]. The phycocyanin, carotenoids, vitamins, lipids, proteins, and carbohydrates belong to the major phytoconstituents that exist in the *C. equisetifolia* extract and exhibit antioxidant and scavenging activities as proposed by Dib et al. [105] and supported consequently by Aboulthana et al. [106]. These components decreased the levels of LPO and TPC content due to scavenging the reactive species that induce lipid peroxidation or by inhibiting xanthine oxidase, a recognized enzyme that generates free radicals [107]. In addition, the presence of Au-NPs in the nano-extract enhanced the antioxidant properties and demonstrated higher protective role against ROS [33].

### Inflammatory and fibrotic markers

Cytokines (small molecular weight proteins) are considered as biomarkers for inflammation, immune reactivity, tissue injury, and repair [108]. In the current study, the pro-inflammatory cytokines (TNF-α and IL-6) elevated in liver and brain tissues of CPF-injected group significantly and this was coincided with the findings obtained by Abolaji et al. [109] who suggested that the inflammasome and subsequent innate immune response were stimulated by the mitochondrial oxidative stress mediated by CPF. Therefore, the ROS scavengers might be used to repair the OP compound–induced lesions [110]. Level of hydroxyproline (fibrotic marker) increased in the CPF-injected group, and this might refer to inducing damage to intestinal barrier and increasing the intestinal permeability [111] and/or due to translocation of gut bacteria to liver, leading to observed inflammatory reaction [112].

The CPF injection reduced the activity of AChE enzyme in both liver and brain tissues, and this was in accordance with Mohamed et al. [89] who proposed that the decrease in AChE activity is strongly related to excessive generation of the ROS that affect the enzyme activity due to a possible oxidative injury to cholinergic cells. Moreover, AChE activity might be decreased as a result of the microsomal oxidation in the liver and formation of the active metabolite CPF-oxone forms (a potent inhibitor of AChE in the peripheral tissues) that were generated after hepatic metabolism of CPF by cytochrome P450 2B6 enzyme and this metabolite possesses inhibiting potency higher than that of the parent compounds [113]. By inhibiting AChE activity, accumulation of acetylcholine is increased by CPF, stimulating its receptors more and downregulating them as a result [114]. In brains of rats, CPF caused significant inhibition in the AChE and this was in accordance with Imam et al. [115] who emphasized that AChE inhibition is categorized as the primary mechanism of the CPF action affecting redox processes and the lipid peroxidation that are greatly implicated in the chronic outcomes following the CPF exposure. Pre-administration of the nano-extract ameliorated the inflammation induced as a result of intoxication of CPF. This was in agreement with Rahmouni et al. [116] who demonstrated that the nano-extract is rich in flavonoids and other constituents with polyphenolic structures enable them to possess anti-inflammatory properties by decreasing levels of the pro-inflammatory biomarkers. The presence of the Au-NPs in the plant extract interferes with transmission of inflammatory signaling, and this is mostly attributed to the extracellular interactions with IL-6 which aggregates around Au-NPs, thus inhibiting IL-6 binding to cellular receptors [117]. The Au-NPs decreased the progression of inflammation by decreasing mRNA expression specific to TNF-α and IL-6 in addition to inducible nitric oxide synthase [118].

### Histopathological examination

The biochemical and histopathological studies carried out by Almazroo et al. [119] showed that the liver and brain are the most target organs susceptible for CPF accumulation. During the current study, the microscopic examination of the liver in the CPF-injected group revealed various histopathological changes represented by severe alterations in the architecture of the liver with increasing degeneration of the hepatic cells. These changes are consistent with the biochemical measurements assessed during our investigation. This agreed with Tanvir et al. [88] who revealed that the CPF can easily cross the cell membrane due to its lipophilic properties causing damage inside the liver (an organ responsible for detoxification). The histopathological changes in the CPF-injected group might be related to the oxidative stress that causes severe lesions in the hepatocytes due to decreasing measurements of the antioxidant system in liver tissues, leading to overproduction of ROS and hence injury then death of healthy cells [106]. In regard to brain tissues, severe histopathological alterations were detected in the CPF-injected group and this agreed with Altun et al. [120] who proposed that the hemorrhages in brain tissues were caused by the acute CPF intoxication. Latuszynska et al. [121] added that the CPF caused numerous histopathological changes, including formation of eosinophilic plagues, hemorrhage in the covering meninges, and pyknosis in the cerebellum area with edema and pyknosis in the cerebral cortex accompanied with accumulation of the cytoplasm in neurocytes. The oxygen, hydroxyl, and hydroperoxyl radicals belong to ROS that were produced mostly in mitochondria during electron transport chain to form peroxynitrite (disrupting the redox status) after the interaction with NO [122]. Treatment of the CPF-injected rats with Au-*C. equisetifolia* nano-extract might protect the liver and brain tissues against oxidative lesions induced by CPF. It caused less histopathological changes in liver tissues than in the CPF-injected group, indicating its protective effects and stimulated restoration of the total number and mass of hepatocytes. This might be attributed to existence of the flavonoids that exhibit ability to donate electrons or hydrogen molecules [123]. These constituents are well known by their ability to act as free radical scavengers to prevent oxidative damage and to stabilize membranes [124]. Furthermore, the nano-extract decreased scores of the histopathological damage due to existence of the Au-NPs that exhibit antioxidant and the anti-inflammatory effects. In agreement with the previous findings, other investigators reported that Au-NPs reduced leukocyte migration and also macrophage infiltration [125].

### Electrophoretic patterns

Electrophoresis is used for detecting the mutagenic differences that may occur by hiding normal bands and/or existing abnormal ones in the different protein and isoenzyme patterns [126]. Percent of the SI is related the physiological state of tissue, and it is inversely proportional to the qualitative alterations [127].

The present study showed that CPF caused qualitative abnormalities in the native electrophoretic protein patterns in liver and brain tissues. Changing composition of the native protein might be related to altering function and organization of the DNA and hence affect expression of different proteins [128]. Also, the disturbances in the protein pattern might refer to the role of CPF in inhibiting expression of some genes or activating the others to produce specific mRNAs which may be translated subsequently into specific proteins and/or due to altering metabolism (synthesis/degradation) of various proteins [129]. Furthermore, appearance of new protein bands in the liver and brain of CPF-injected rats might refer to changing the cytoplasmic protein due to inhibiting the protein anabolic actions and/or due to altering the functional conformations of the structural proteins, resulting in denaturation of the high molecular weight proteins [130].

Lipids possess an important impact in maintaining the integrity of the cell membrane [131]. The present study demonstrated that CPF caused physiological variations in lipid moiety of native protein pattern in liver tissue and this agreed with Kalender et al. [8] who showed that CPF affected the permeability of the hepatocyte membranes and might be attributable to the blockage of the bile duct which reduces the secretion of cholesterol into the duodenum. Also, this might refer to increasing the hepatic synthesis and/or diminishing hepatic degradation of lipids due to the reducing activity of the lipoprotein lipase [132]. In the brain of CPF-injected group, the alterations in the protein pattern might refer to its adverse effect on the cellular lipids, causing abnormal synthesis or defective degradation of lipids [133]. Moreover, the peroxidation products that accumulated due to depletion of the antioxidant defenses affect the lipid portion by inducing the formation of the ROS that leads consequently to modifications of the lipid moieties oxidatively and hence their denaturation [134].

Calcium-binding proteins are acidic proteins with low molecular weights, exhibiting a crucial role in resistance of the tissues against the deleterious effects induced by the toxic substances. They altered the mineralization process by inhibiting the formation of the hydroxyapatite [135]. The current study showed that CPF injection caused changes in calcium moiety of the native protein and this agreed with Abulyazid et al. [136] who reported that the abnormal tissue mineralization is responsible for altering this native protein pattern. El-Sayed et al. [134] demonstrated that the changes in quality and quantity of this native protein pattern might refer to overproduction of the ROS that convert an active hydrogen atom from these biologically macromolecules. Moreover, these alterations might be related to an increasing concentration of calcium which stimulates oxidative stress in the tissue, leading to variations in this protein pattern [137].

The treatment of the CPF-injected group with gold nano-extract had an ameliorative effect against the changes induced by the attack of free radical in the different electrophoretic protein patterns. This could allude to the existence of the active constituents (polyphenolic compounds) that elevated as a result of incorporation of M-NPs [138]. The nano-extract ameliorated the lipid moiety of native protein pattern due to presence of the phytoconstituents that exhibit lipolysis-stimulating agent in adipocytes. This lipolytic action was mainly mediated by phosphorylation of hormone-sensitive lipase which is regulated post-transcriptionally by reversible phosphorylation by protein kinase A [139].

Activities of the antioxidant enzymes vary from tissue to tissue. Therefore, they are considered as a tissue-dependent tool. The changes in their activities in the CPF-injected group might be related to degenerating the protein contents or altering the metabolic pathways due to the attack of the ROS targeting protein contents [140]. As shown in the current study, CPF altered the electrophoretic CAT pattern, which may be explained by the fact that when CPF binds to native macromolecules, it causes modifications in the secondary structure of the enzyme [141]. The abnormalities in the electrophoretic POX pattern might be related to the peroxidation reactions stimulated by the oxidative stress [142]. Additionally, ROS may have an impact on these enzymes directly or on the protein component of these enzymes, which would explain the decline in their activity. Hence, these ROS have the ability to alter the physico-chemical and immunologic characteristics of endogenous CAT and POX enzymes.

The α-amylase belongs to the digestive enzymes that catalyze hydrolysis of the α-(1,4)-d-glycosidic bonds of starch and other glucose polymers, which leads to the conversion of dietary carbohydrates to oligosaccharides and disaccharides [143]. The present study demonstrated that CPF altered the electrophoretic α-Amy isoenzyme pattern and this might refer to changing the fractional activity that is correlated with altering the rate of protein expression secondary to DNA damage caused oxidatively by ROS [127]. Abulyazid et al. [136] added that the oxidative stress induced structural changes in protein portion of native enzymes. The alterations in expression of the different proteins are responsible for changing the enzymatic activities. Moreover, the alterations in the α-Amy isoenzyme pattern might be related to the hormonal and metabolic alterations [144]. Esterases are a wide family of polymorphic and tissue-specific lysosomal lipolytic enzymes that are distinguished by their capacity to catalyze hydrolyzing ester linkages in neutral lipids into the corresponding carboxylic acids [145]. The brain is rich in EST enzymes that exist in membrane structures and exhibit their role in the neurotransmission and communication of messages by stimulating the breakdown of acetylcholine liberated during nervous stimulation [146]. The present study showed that the CPF injection caused mutagenicity in the electrophoretic α- and β-EST patterns due to changing fluidity of the membrane, reflecting alterations in the physical state of the membrane lipids. Therefore, these membrane-bound enzymes are sensitive to these deleterious effects [147]. Also, these variations might be directly related to excessive generation of the ROS which stimulate the fragmentation of polypeptide chain through sulfhydryl-mediated cross-linking of labile amino acids affecting the integrity of protein molecule [148]. The gold nano-extract ameliorated the electrophoretic isoenzyme patterns due to existence of the active phytoconstituents that stimulate the antioxidant status of the tissue to resist the attack of the ROS targeting these biomacromolecules [149]. Incorporation of the Au-NPs increased the polyphenolic compounds and hence enhanced the ameliorative effect of the extract in maintaining the integrity of these biomacromolecules against the adverse effect induced by oxidative reactions [85].

### Molecular assay

The current study showed that the CPF injection increased levels of the relative expression levels of caspase-3, p^53^, and Bax genes associated with decreasing expression of the Bcl2 gene in liver tissues. This might be attributable to the role of CPF in upregulating the pro-apoptotic mediators in this tissue [89] in addition to the role of the excessive ROS in inducing cell apoptosis in the CPF-injected group [73]. In brain tissues, levels of the relative expression of the apoptotic genes increased in the CPF-injected group due to inducing transcription of the critical genes required for apoptosis [150]. The relative expression of the Bcl2 protein decreased with increasing the relative Bax expression contrarily in both cerebrum and cerebellum. The relative expression of these proteins changed due to initiation of apoptotic machinery by ROS generation and releasing the stress signal in response to CPF intoxication [151]. Treatment of CPF-injected rats with Au-*C. equisetifolia* nano-extract regulated the expression of the pro- and anti-apoptosis mediators and produced a synergistic effect on the expression of apoptosis-related proteins by decreasing the expression of p^53^, Bax, and caspase-3 with increasing the Bcl2 expression. This agreed with El Gamal et al. [152] who demonstrated that the presence of flavonoids and polyphenolic chemicals, which have potent antioxidant, radical scavenging, and anti-inflammatory properties, may be responsible for upregulation of anti-apoptotic proteins and downregulation of pro-apoptotic proteins. Also, the presence of the Au-NPs demonstrated strong antioxidant activity and was capable of simulating the activity of antioxidant enzymes by interacting with hydroxyl radicals and superoxide anions directly to produce less-reactive side products [85, 153]. Zhou et al. [154] demonstrated that Au-NPs directly bind and neutralize reactive species, and this effect depends on the size and surface of the Au-NPs.

## Conclusions

Our results proved that the alterations induced by CPF were diminished by Au-*C. equisetifolia* nano-extract which restore levels of the hematological and biochemical parameters in all nano-extract-treated groups to normalcy. It normalized measurements of the antioxidant system and restored levels of the inflammatory and fibrotic markers in both liver and brain tissues in the nano-extract pre-treated group. Moreover, severity of the histopathological lesions induced in these tissues by the CPF was decreased by the nano-extract which showed the highest ameliorative efficiency in the pre-treated group. The different electrophoretic protein and isoenzyme patterns altered in the CPF-intoxicated group were ameliorated by the nano-extract especially in the pre-treated group.

### Supplementary Information

**Additional file 1: Supplementary Table 1. **The primers with the sequences suitable to be used for *qRT-PCR*. **Supplementary Table 2.** Effect of *C. equisetifolia* extract incorporated with gold nanoparticles (Au-NPs) against toxicity induced by chlorpyrifos (CPF) on different hematological measurements in rats. Data were calculated from five replicates and expressed as mean ± SE. ^a^Significant versus control group, ^b^Significant versus toxic (CPF) group at *P*≤0.05. **Supplementary Table 3.** Effect of *C. equisetifolia* extract incorporated with gold nanoparticles (Au-NPs) against toxicity induced by chlorpyrifos (CPF) on different biochemical measurements in rats. Data were calculated from five replicates and expressed as mean ± SE. ^a^Significant versus control group, ^b^Significant versus toxic (CPF) group at *P*≤0.05. **Supplementary Figure 1.** Effect of *C. equisetifolia* extract incorporated with gold nanoparticles (Au-NPs) against toxicity induced by chlorpyrifos (CPF) on **a)** the change in body weights, **b)** the relative organ weights (organ/body weights ratio) of rats. Data were calculated from five replicates and expressed as mean ± SE. ^a^Significant versus control group, ^b^Significant versus toxic (CPF) group at *P*≤0.05.

**Additional file 2. **

**Additional file 3. **

## Data Availability

This paper has data included as electronic supplementary material.

## Abbreviations

AChE: Acetylcholinesterase
ALP: Alkaline phosphatase
ALT: Alanine aminotransferase
ANOVA: Analysis of variance
AST: Aspartate aminotransferase
Au-NPs: Gold nanoparticles
Aβ: β-Amyloid
B%: Band percent
BAX: Bcl2-associated X protein
Bcl2: B-cell leukemia/lymphoma
CAT: Catalase
CBB: Coomassie brilliant blue
CK: Creatine kinase
CPF: Chlorpyrifos
DMSO: Dimethyl sulfoxide
EST: Esterase
GGT: Gamma-glutamyl aminotransferases
GPx: Glutathione peroxidase
GSH: Reduced glutathione
HB: Hemoglobin
HCT: Hematocrit
HDL-c: High-density lipoprotein cholesterol
IL-6: Interleukin-6
Int.: Band intensity
LD_50_: Median lethal dose
LDH: Lactate dehydrogenase
LDL-c: Low-density lipoprotein cholesterol
LPO: Lipid peroxidation product
MCH: Mean corpuscular hemoglobin
MCHC: Mean corpuscular hemoglobin concentration
MCV: Mean corpuscular volume
M-NPs: Metal nanoparticles
MPV: Mean platelet volume
OP: Organophosphorus
p^53^: Tumor suppressor protein 53
PAGE: Polyacrylamide gel electrophoresis
PLT: Platelet
RBCs: Red blood cells
RDW: Red blood cell distribution width
Rf: Relative mobility
ROS: Reactive oxygen species
SBB: Sudan Black B
SE: Standard error
SI%: Percentage of similarity index
SOD: Superoxide dismutase
TAC: Total antioxidant capacity
TC: Total cholesterol
TGs: Triglycerides
TNF-α: Tumor necrosis factor-α
TPC: Total protein carbonyl
WBCs: White blood cells
α-Amy: α-Amylase
