# Supplementary material for: The hepato- and neuroprotective effect of gold Casuarina equisetifolia bark nano-extract against Chlorpyrifos-induced toxicity in rats
Source: J Genet Eng Biotechnol. 2023 Dec 1;21:158. doi: 10.1186/s43141-023-00595-6 (PMC10692062; doi:10.1186/s43141-023-00595-6)
Supplement: Supplementary file 1 — Additional file 1: Supplementary Table 1. The primers with the sequences suitable to be used for qRT-PCR. Supplementary Table 2. Effect of C. equisetifolia extract incorporated with gold nanoparticles (Au-NPs) against toxicity induced by chlorpyrifos (CPF) on different hematological measurements in rats. Data were calculated from five replicates and expressed as mean ± SE. aSignificant versus control group, bSignificant versus toxic (CPF) group at P≤0.05. Supplementary Table 3. Effect of C. equisetifolia extract incorporated with gold nanoparticles (Au-NPs) against toxicity induced by chlorpyrifos (CPF) on different biochemical measurements in rats. Data were calculated from five replicates and expressed as mean ± SE. aSignificant versus control group, bSignificant versus toxic (CPF) group at P≤0.05. Supplementary Figure 1. Effect of C. equisetifolia extract incorporated with gold nanoparticles (Au-NPs) against toxicity induced by chlorpyrifos (CPF) on a) the change in body weights, b) the relative organ weights (organ/body weights ratio) of rats. Data were calculated from five replicates and expressed as mean ± SE. aSignificant versus control group, bSignificant versus toxic (CPF) group at P≤0.05. [file 43141_2023_595_MOESM1_ESM.docx]

**Supplementary Table 1.** The primers with the sequences suitable to be used for *qRT-PCR*.

| **Gene** | **Primers sequences (5' - 3')** | **NCBI Reference** |
| --- | --- | --- |
| BAX | **F:** TCA TGA AGA CAG GGG CCT TT  **R:** GTC CAC GTC AGC AAT CAT CC | NM_007527.3 |
| Bcl2 | **F:** TTG TAA TTC ATC TGC CGC CG  **R:** AAT GAA TCG GGA GTT GGG GT | NM_009741.5 |
| p53 | **F:** ACA GTC GGA TAT CAG CCT CG  **R:** GCT TCA CTT GGG CCT TCA AA | AB021961.1 |
| Caspase-3 | **F:** gct tca ctt ggg cct tca aa  **R:** aca ggc cca ttt gtc cca ta | NM_001284409.1 |
| GAPDH | **F:** caa ctc cca ctc ttc cac ct  **R:** gag ttg gga tag ggc ctc tc | NM_001289726.1 |


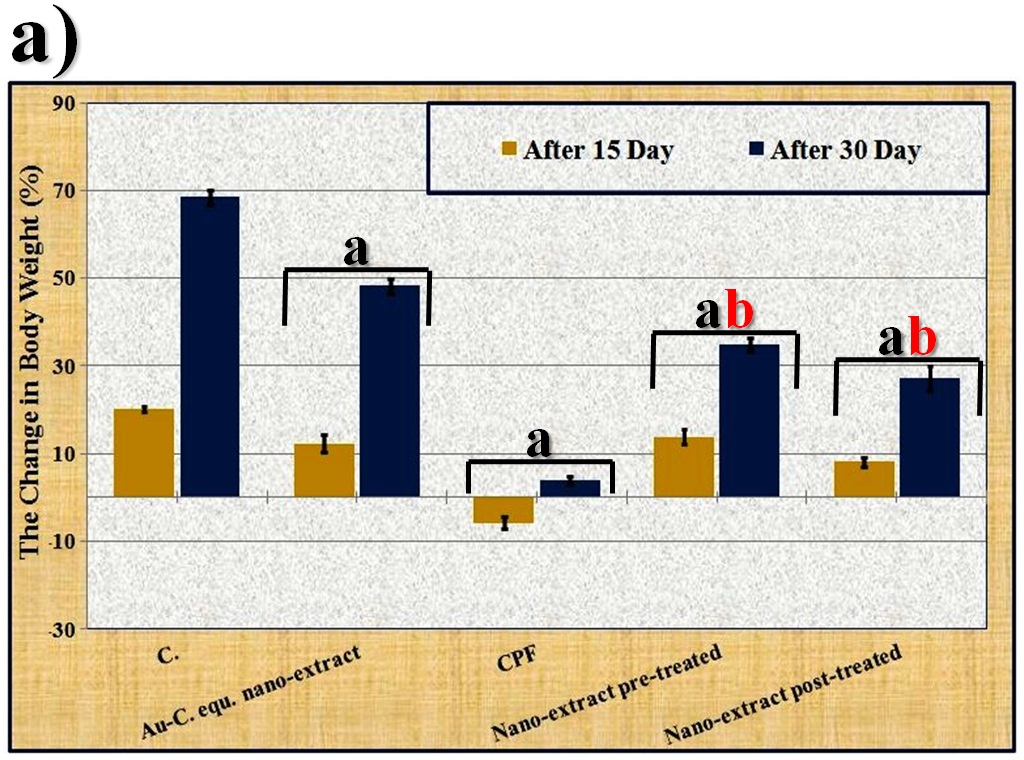

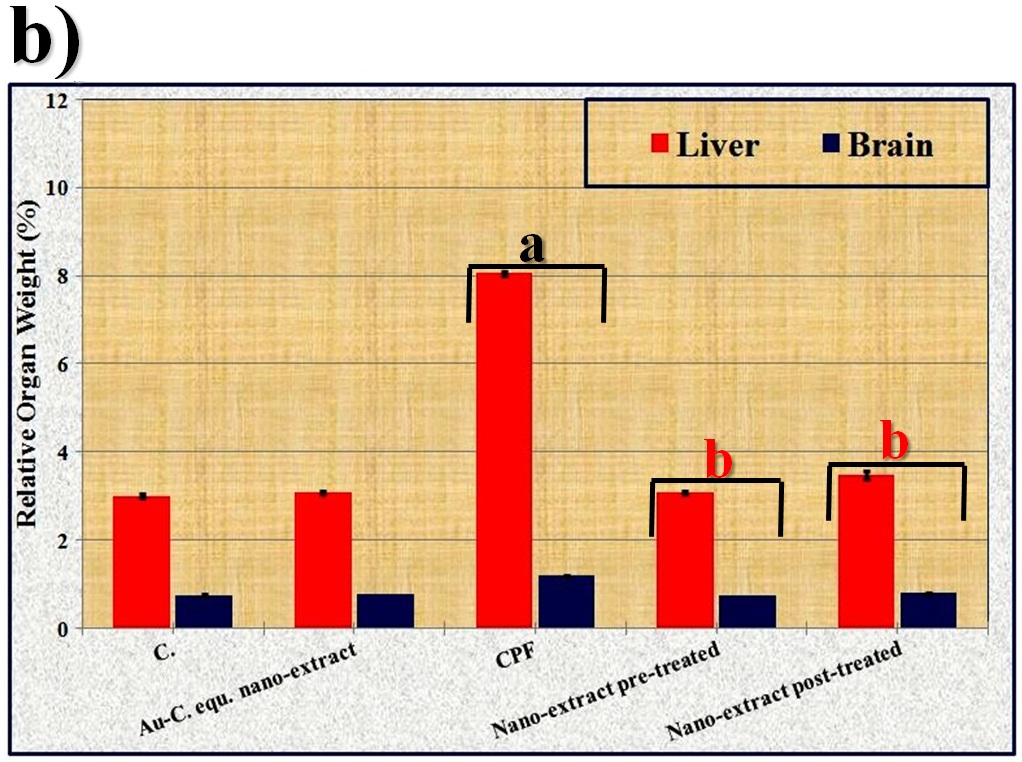


**Supplementary Figure 1.** Effect of *C. equisetifolia* extract incorporated with gold nanoparticles (Au-NPs) against toxicity induced by chloropyrifos (CPF) on **a)** the change in body weights, **b)** the relative organ weights (organ/body weights ratio) of rats. Data were calculated from five replicates and expressed as mean ± SE, **a**: significant versus control group, **b**: significant versus toxic (CPF) group at P≤0.05.

**Supplementary Table 2.** Effect of *C. equisetifolia* extract incorporated with gold nanoparticles (Au-NPs) against toxicity induced by chloropyrifos (CPF) on different hematological measurements in rats.

|  | | **C.** | **Au-*C. equisetifolia* nano-extract** | **CPF** | **CPF + Au-*C. equisetifolia* nano-extract** | |
| --- | --- | --- | --- | --- | --- | --- |
|  |  |  |  |  | **Pre-treated** | **Post-treated** |
| **Formed Elements** | **RBCs (**10^6^/ul**)** | 6.30 ± 0.01 | 6.11 ± 0.20 | 3.62 ± 0.03**^a^** | 6.29 ± 0.01**^b^** | 6.30 ± 0.01**^b^** |
|  | **HB (**g/dl**)** | 13.26 ± 0.01 | 13.27 ± 0.01 | 7.62 ± 0.05**^a^** | 13.24 ± 0.03**^b^** | 13.26 ± 0.04**^b^** |
|  | **HCT (**%**)** | 39.84 ± 0.13 | 39.70 ± 0.10 | 22.92 ± 0.11**^a^** | 39.80 ± 0.16**^b^** | 39.90 ± 0.19**^b^** |
|  | **MCV (**um^3^**)** | 58.83 ± 0.08 | 58.87 ± 0.07 | 33.84 ± 0.25**^a^** | 58.76 ± 0.16**^b^** | 58.83 ± 0.15**^b^** |
|  | **MCH (**pg**)** | 20.76 ± 0.10 | 20.79 ± 0.18 | 20.92 ± 0.11 | 20.73 ± 0.07 | 20.75 ± 0.08 |
|  | **MCHC (**g/dl**)** | 36.63 ± 0.06 | 36.68 ± 0.08 | 36.72 ± 0.11 | 36.83 ± 0.10 | 36.86 ± 0.08 |
|  | **RDW (**%**)** | 15.19 ± 0.01 | 15.19 ± 0.01 | 10.614 ± 0.15**^a^** | 15.21 ± 0.04**^b^** | 15.19 ± 0.03**^b^** |
|  | **MPV (**um^3^**)** | 8.10 ± 0.01 | 8.11 ± 0.01 | 6.49 ± 0.17**^a^** | 8.1 ± 0.02**^b^** | 8.09 ± 0.02**^b^** |
|  | **PLT (**10^3^/ul**)** | 445.00 ± 0.84 | 446.80 ± 1.02 | 243.79 ± 0.97**^a^** | 445.54 ± 1.33**^b^** | 445.01 ± 1.69**^b^** |
|  | **WBCs (**10^3^/ul**)** | 7.85 ± 0.02 | 7.84 ± 0.03 | 4.51 ± 0.04**^a^** | 7.84 ± 0.03**^b^** | 7.85 ± 0.02**^b^** |
| **Differential Count** | **Lymp. (**10^3^/ul**)** | 6.37 ± 0.01 | 6.37 ± 0.01 | 3.66 ± 0.02**^a^** | 6.36 ± 0.01**^b^** | 6.37 ± 0.02**^b^** |
|  | **Mono. (**10^3^/ul**)** | 0.80 ± 0.01 | 0.79 ± 0.01 | 0.46 ± 0.01**^a^** | 0.80 ± 0.01**^b^** | 0.80 ± 0.01**^b^** |
|  | **Gran. (**10^3^/ul**)** | 0.62 ± 0.00 | 0.62 ± 0.00 | 0.36 ± 0.00**^a^** | 0.62 ± 0.00**^b^** | 0.62 ± 0.00**^b^** |

Data were calculated from five replicates and expressed as mean ± SE, **a**: significant versus control group, **b**: significant versus toxic (CPF) group at P≤0.05.

**Supplementary Table 3.** Effect of *C. equisetifolia* extract incorporated with gold nanoparticles (Au-NPs) against toxicity induced by chloropyrifos (CPF) on different biochemical measurements in rats.

|  | | **C.** | **Au-*C. equisetifolia* nano-extract** | **CPF** | **CPF + Au-*C. equisetifolia* nano-extract** | |
| --- | --- | --- | --- | --- | --- | --- |
|  |  |  |  |  | **Pre-treated** | **Post-treated** |
| **Liver** | **ALT (**U/L**)** | 48.60 ± 0.60 | 48.80 ± 0.74 | 73.84 ± 0.57**^a^** | 49.20 ± 0.37**^b^** | 61.40 ± 0.40**^ab^** |
|  | **AST (**U/L**)** | 97.40 ± 0.25 | 97.00 ± 0.32 | 148.30 ± 1.00**^a^** | 97.20 ± 0.59**^b^** | 125.26 ± 0.49**^ab^** |
|  | **ALP (**U/L**)** | 188.80 ± 0.58 | 189.00 ± 0.71 | 296.66 ± 0.64**^a^** | 188.60 ± 0.51**^b^** | 229.80 ± 0.37**^ab^** |
|  | **GGT (**U/L**)** | 21.14 ± 0.01 | 21.15 ± 0.01 | 35.14 ± 0.01**^a^** | 21.15 ± 0.01**^b^** | 28.14 ± 0.01**^ab^** |
| **Kidney** | **Urea (**mg/dl**)** | 30.38 ± 0.26 | 30.40 ± 0.25 | 55.01 ± 0.04**^a^** | 30.39 ± 0.25**^b^** | 41.28 ± 0.01**^ab^** |
|  | **Creat. (**mg/dl**)** | 1.09 ± 0.00 | 1.09 ± 0.00 | 1.69 ± 0.01**^a^** | 1.09 ± 0.00**^b^** | 1.42 ± 0.00**^ab^** |
|  | **Uric Acid (**mg/dl**)** | 3.19 ± 0.01 | 3.19 ± 0.01 | 6.19 ± 0.01**^a^** | 3.19 ± 0.01**^b^** | 4.97 ± 0.01**^ab^** |
|  | **T. Protein (**g/dl**)** | 5.97 ± 0.01 | 5.97 ± 0.01 | 3.74 ± 0.01**^a^** | 5.94 ± 0.02**^b^** | 4.96 ± 0.01**^ab^** |
|  | **Albumin (**g/dl**)** | 3.88 ± 0.01 | 3.88 ± 0.01 | 1.11 ± 0.01**^a^** | 3.88 ± 0.01**^b^** | 2.86 ± 0.01**^ab^** |
| **Heart** | **CK (**U/L**)** | 123.20 ± 0.37 | 123.40 ± 0.51 | 193.42 ± 0.59**^a^** | 123.40 ± 0.51**^b^** | 151.54 ± 0.46**^ab^** |
|  | **LDH (**U/L**)** | 242.40 ± 0.40 | 242.20 ± 0.37 | 380.57 ± 0.63**^a^** | 242.20 ± 0.37**^b^** | 298.15 ± 0.49**^ab^** |
| **Lipid Profile** | **TC (**mg/dl**)** | 78.40 ± 0.25 | 78.20 ± 0.37 | 99.60 ± 0.81**^a^** | 78.20 ± 0.37**^b^** | 89.00 ± 0.32**^ab^** |
|  | **T.Gs (**mg/dl**)** | 72.80 ± 0.37 | 72.80 ± 0.37 | 93.20 ± 0.37**^a^** | 72.80 ± 0.37**^b^** | 82.40 ± 0.25**^ab^** |
|  | **LDL-c** **(**mg/dl**)** | 24.44 ± 0.41 | 24.44 ± 0.50 | 56.76 ± 0.92**^a^** | 24.24 ± 0.41**^b^** | 42.12 ± 0.38**^ab^** |
|  | **HDL-c** **(**mg/dl**)** | 39.40 ± 0.25 | 39.20 ± 0.37 | 24.20 ± 0.37**^a^** | 39.40 ± 0.25**^b^** | 30.40 ± 0.25**^ab^** |

Data were calculated from five replicates and expressed as mean ± SE, **a**: significant versus control group, **b**: significant versus toxic (CPF) group at P≤0.05.
