# Supplementary material for: The hepato- and neuroprotective effect of gold Casuarina equisetifolia bark nano-extract against Chlorpyrifos-induced toxicity in rats
Source: J Genet Eng Biotechnol. 2023 Dec 1;21:158. doi: 10.1186/s43141-023-00595-6 (PMC10692062; doi:10.1186/s43141-023-00595-6)
Supplement: Supplementary file 2 — Additional file 2. [file 43141_2023_595_MOESM2_ESM.doc]

**نموذج إقرار للباحثين بشأن**

**الرعاية الجيدة للحيوانات المستخدمة في البحوث**

|  | إسم الباحث الرئيس في البحث: وائل محمود كامل أبو الثنا |  |
| --- | --- | --- |
|  | تخصص الباحث المتعامل مع حيوانات التجارب: الكيمياء الحيوية |  |
|  | العنوان: 10 شارع قمر متفرع من شارع معهد الأبحاث - وراق الحضر- محافظة الجيزة |  |
|  | المحمول: 01095522103 تليفون أرضي: 0235421367 |  |
| عنوان البحث باللغة الإنجليزية:  **Evaluation of the ameliorative Effect of *Casuarina equisetifolia* Bark Extract after Incorporating Gold Nanoparticles against Toxicity Induced by Pesticides in Rats** | |  |
|  | عنوان البحث باللغة العربية:  **تقييم التأثير التحسيني لمستخلص لحاء شجرة الكازارينا بعد دمجها بجسيمات الذهب النانومترية ضد السمية المستحثة بالمبيدات في الجرذان** |  |
|  | نوع الحيوان المستخدم و العدد: جرذان تجارب غير صالحة للأكل  Seventy-two (72) adult albino mice and eighty (80) adult male Wistar albino rats |  |
|  | الهدف من إجراء البحث: زيادة كفاءة مستخلص لحاء شجرة الكازارينا عن طريق دمجه بجسيمات الذهب النانومترية وعمل دراسة مقارنة لكشف المستخلص المدمج الأكثر فاعلية ودراسة كفائته ضد السمية التي تحدث كنتيجة للتعرض لمبيدات الفسفور العضوي وتحديدا مادتي الكلوروبايروفوس والإثيون في جرذان التجارب. |  |
|  | الفائدة العامة من البحث: إنتاج منتج نباتي فعال ضد الخلل الذي قد يحدث في الوظائف الحيوية كنتيجة للتعرض لبعض الملوثات البيئية. |  |
|  | الفائدة المرجوة للحيوانات في البحث: تقضي حياتها طول فترة التجربة تحت ظروف بيئية نظيفة ومعينة وفي الآخر تستهلك في خدمة العلم والبشرية وتساهم في إنتاج منتجات نباتية قد تساعد في حماية أوعلاج خلل الوظائف الحيوية. |  |
|  | مكان إيداع الحيوانات طوال فترة البحث: بيت الحيوان بالمركز القومي للبحوث. |  |
|  | تفصيل ما سوف يتم إجرائه على الحيوانات المستخدمة في البحث:  **1. Preliminary Phytochemical Screening Tests**  The total carbohydrates and /or glycosides, free and combined flavonoids, coumarins, saponins, alkaloids, nitrogenous compounds, sterols and /or triterpenes, tannins, proteins and anthraquinones will be determined in *Casuarina equisetifolia* bark.  **2. Yields, physical and chemical characters will be determined in the crude alcoholic, petroleum ether and aqueous extracts**  **3. Determination of total phenolic compounds**  The total polyphenols will be estimated using Folin-Ciocalteu reagent according to method suggested by **Singleton and Rossi (1965)**.  **4. Investigation of the lipoidal matter**  4.1. The total steroidal, terpenoidal and free fatty acids contents will be quantitatively estimated.  4.2. The free fatty acids will be converted into the methylated ester form then analyzed by gas chromatography / mass spectrometer (GC/MS) technique.  4.3. The major steroidal and/or terpenoidal compounds will be isolated from the petroleum ether extract then identified using different spectrophotometric techniques.  **5. Chromatographic and spectrophotometric analysis of the isolated compounds**  Due to the high solubility (in water and/or organic solvents), the HPLC can offer a suitable tool for qualitative and quantitative analysis. Modern detection methods, such as mass spectrometry (MS) and nuclear magnetic resonance (NMR), will be combined with HPLC **(Schutz *et al*., 2006)**, allowing rapid structural analysis and identification of compounds with minimal manipulation of the sample.  **6. Preparation of sample extract for Au-NPs biosynthesis**  The *C. equisetifolia* bark will be weighed, thoroughly washed by double deionised water to remove surface impurities. It will be crushed using a blender and finely macerated. After homogenization, 100ml double deionised water will be added and heated over water bath maintained at 80 °C for 15 minutes. The extract obtained will be filtered through *Whatmann No*.1 filter paper (pore size 25 and used immediately for the biosynthesis of nanoparicles).  **7. *In vitro* antioxidant and cytotoxic activities of the different extracts**  All these measurements will be assayed in the different plant extracts. Also, these assays will be carried out in the extracts before and after incorporating nanoparticles.  7.1. Total polyphenolic compounds  Concentration of the total polyphenols will be estimated in the different plant extracts and nano-extracts by folin ciocalteu reagent using gallic acid as standard according to method described by **Singleton and Rossi (1965)**.  7.2. Total antioxidant capacity  The total antioxidant capacity of extracts and nano-extracts will be evaluated through the assay of the green phosphate/Mo^5+^ complex according to the method described by **Prieto *et al*. (1999)**.  7.3. Total reducing power  The total reducing power will be determined according to method suggested by **Oyaizu (1986)**.  7.4. Free radical scavenging activity  The scavenging activities will be determined against the free radicals initiated by1,1-Diphenyl-2-picryl-hydrazyl (DPPH) based on the method described by **Brand-Williams *et al*. (1995)**.  7.5. Anticancer activity  Cytotoxic activity test (*In vitro* bioassay on human tumor cell lines) will be conducted and determined. It will be performed on human hepatocellular carcinoma cell line (HepG2) based on the method suggested by **Mosmann (1983)** and human colon carcinoma cell line according to protocol suggested by **Vichai and Kirtikara (2006)**. All the tumor cells will be purchased from CSIR-National Chemical Laboratory, Pune, India.  **8. Synthesis of gold nanoparticles (Au-NPs)**  Based on the in vitro antioxidants (total polyphenolic compounds, total antioxidant capacity, total reducing power and scavenging activities against free radicals initiated by DPPH in addition to the cytotoxic activity against growth of human liver and colon carcinoma) that will be assayed in *C. equisetifolia* bark, the most effective plant extracts will be selected to be incorporated by Au-NPs.  8.1. Preparation of gold nanoparticles (Au-NPs)  Generally, the fabrication of Au-NPs via the chemical reduction method will be carried out through two main parts: (I) the first part will be concerned with reduction of Au^+3^ (HAuCl_4_) to Au^0^. For this purpose, the reduction reaction between tetrachloroauric acid (HAuCl) and trisodium citrate (Na_3_C_6_H_5_O_7_.2H_2_O) will be used in an aqueous solution. The second part will be the stabilization using cetyltrimethylammonium bromide (CTAB) in order to avoid aggregation of the particles **(Zhao *et al*., 2013)**.  8.2. Preparation of cellulose nanocrystal (CNC)  A desired amount of dried rice straw powder will be weighed and transferred into a round bottom flask. Alkali solution (4-wt % NaOH) will be added and the treatment will be performed at reflux condition at 100-120ºC for 2 h. The mixture will be then filtered and washed with distilled water several times to remove lignin and hemicellulose that dissolved in the solution. The resulting fiber will be dried before used for bleaching treatment. The bleaching treatment will be performed at reflux condition at 110-130 ºC for 4 hours after adding 30 g of fiber into 200 ml of each solution of 1.7% NaClO_2_, acetic buffer and distilled water. The mixture will be then allowed to cool before filtered and washed with distilled water until white cellulose will be obtained. The cellulose obtained will be dried by using freeze dryer (Labconco) at -39ºC for 24 h. Cellulose nanocrystal will be prepared by using sulfuric acid hydrolysis. A 65 wt% H_2_SO_4_ will be prepared before approximately 5% of cellulose fiber will be added to the solution. The time and temperature will be fixed at 45 °C for 45 min in order to achieve the optimum yield. The hydrolyzed cellulose sample will be washed five times by centrifugation (10,000 rpm, 10 min) to remove excess sulphuric acid. The suspension will be then dialyzed against distilled water until a constant pH will be achieved. The resultant cellulose nanocrystal (CNC) suspension will be stored in refrigerator until further used.  8.3. Preparation of gold nano-extract  Nanoemulsion of the plant extracts will be prepared using crude plant extracts, non-ionic surfactant Tween 20 (HLB-16.7), crystline nanocellulose (CNC) and water via spontaneous emulsification method. Nanoemulsion will be carried out in two steps: in the first step, organic phase will be fabricated through mixing plant crude sample with the chosen surfactant (Tween20) in the following ratio (1:5) then 3 gm of CNC will be added and the mixture will be sonicated for 30 min. In the second stage, the organic phase (Plant extract, Tween 20 and CNC) will be added drop by drop (20 ml/min) to water using separating fennel and stirring the system magnetically (800 rpm) at 60 °C for 5 hrs. Then the prepared gold nanoparticles (Au-NPs) will be added to the prepared nanoemulsion by the following ratio (1%) the mixture will be sonicated to another 30 min at 50 °C.  **9. Characterization of biosynthesized nanoparticles**  9.1. X-ray Diffraction (XRD) Studies  The crystal structure of the filler powders will be determined using a Philips X-ray diffractometer (PW 1930 generator, PW 1820 goniometer) equipped with Cu Kα radiation (45 kV, 40 mA, with λ = 0.15418 nm ). The scans of the analysis will run in 2θ range of 5 to 80º with step size of 0.02 and step time of 1s.  9.2. Ultraviolet-Visible (UV-VIS) Spectroscopy  Synthesis of the nanoparticles using plant extracts will be monitored by measuring UV-VIS spectrum of the reaction mixture at λ 200 - 800 nm after 10-fold dilution of the samples with deionised water. The UV-spectroscopy will be carried by Shimadzu UV-Vis recording spectrophotometer UV-240.  9.3. Transmission Electron Microscope (TEM)  This technique will be employed to visualize size and shape of nanoparticles at high resolution level (200 KV). The morphological and particles size of prepared samples will be demonstrated by using TEM model JEM-1230, Japan, operated at 120 kV, with maximum magnification of 600X10^3^ and a resolution until 0.2 nm. A drop of an aqueous dispersion of the prepared samples will be placed on a carbon-coated copper grid and allowed to dry in air before characterization.  9.4. Dynamic Light Scattering (DLS) measurements  Distribution of the synthesized nanoparticles will be measured by photon correlation spectroscopy (PCS) using Malvern Zetasizer Nano ZS (Malvern Instruments Ltd., Malvern, United Kingdom). Samples will be diluted with double distilled water prior to analysis at room temperature with an angle of detection of 90°.  9.5. Fourier Transform Infrared Spectroscopy (FT-IR) analysis  The FT-IR analysis will be carried out by using FT-IR technique manufactured by Bruker depending on the standard method documented by **Eaton *et al*. (1995)**.  **10. Median lethal dose of different extracts (LD_50_)**  The most effective extract (after incorporating Au-NPs) will be studied separately for evaluating the LD_50_. One hundred and sixty adult albino mice (weight 20-25 g) will be divided into 10 groups (8 mice in each group) for calculating the LD_50_ of extract and 10 groups for that of nano-extracts. The groups will be treated orally by stomach tube with rising the doses. Mortality will be recorded after 24 hrs of extract and nano-extract treatment. The LD_50_ will be calculated using equation suggested by **Paget and Barnes (1974)**.  **11. Administration of plant nano-extracts**  The gold nano-extract will be administrated daily by stomach tube at the suitable safe dose taking in the consideration the difference in weight of the animals. According to the therapeutic window, the dose 1/20 of LD_50_ is the most effective and less toxic. Therefore, it will be selected for the therapeutic purposes.  **12. Induction of toxicity by insecticides**  The animals will be fasted for 18 hrs prior to dosing. The insecticides (chloropyrifos ethyl and ethion) that selected to be under study will be administered orally to rats using stomach tube. Volume of the dose will depend on weight of the animals. These compounds will be dissolved in dimethyl sulfoxide (DMSO) **(Raj *et al*., 2013)**. The dose recommended to induce toxicity is 1/20 of LD_50_ of the insecticide used. It was found that the LD_50_ of chloropyrifos ethyl was about 229 mg/kg bw for rats **(Gosselin *et al*., 1984)**. Therefore, the dose that will be administrated will be 22.9 mg/kg bw or 11.45 mg/kg bw. As regard to ethion, it was found that the LD_50_ was about 208 mg/kg bw for rats **(Meister, 1992)**. Therefore, the dose that will be administrated will be 20.8 mg/kg bw or 10.40 mg/kg bw.  **13. Experimental design**  Eighty adult male Wistar rats (weighting 120 - 150 g) will be housed in eight cages (ten per cage). The animals will be provided with water *ad libitum* and standard food and maintained under normal environmental conditions at 25 ± 2 °C. The experimental procedures will be carried out according to the ethical protocol and guidelines approved by the institutional animal care of National Research Centre, Dokki, Giza, Egypt. The rats will be randomly divided into eight groups as the following:  Control group (G1): Rats will be fed with normal diet as *ad libitum* and received distilled water in parallel with DMSO for 28 days.  Gold *C. equisetifolia* bark nano-extract treated group (G2): Rats will be fed with normal diet associated with the treatment with gold *C. equisetifolia* bark nano-extract orally at a dose of 1/20 of LD_50_ for 21 days.  Toxic chloropyrifos ethyl treated group (G3): Rats will be treated orally with chloropyrifos ethyl at a dose of 1/20 of LD_50_ for 28 days.  Toxicity induced by chloropyrifos ethyl and gold nano-extract simult.-treated group (G4): Rats will be treated with chloropyrifos ethyl for 28 days and simultaneously treated by gold *C. equisetifolia* bark nano-extract for 21 days.  Toxicity induced by chloropyrifos ethyl and gold nano-extract post-treated group (G5): Rats will be treated with chloropyrifos ethyl for 28 days followed by the treatment with gold *C. equisetifolia* bark nano-extract orally for another 21 days.  Toxic ethion treated group (G6): Rats will be injected with ethion at a dose of 1/20 of LD_50_ for 28 days.  Toxicity induced by ethion and gold nano-extract simult.-treated group (G7): Rats will be treated with ethion for 28 days and simultaneously treated by gold *C. equisetifolia* bark nano-extract for 21 days.  Toxicity induced by ethion and gold nano-extract post-treated group (G8): Rats will be treated with ethion for 28 days followed by the treatment with gold *C. equisetifolia* bark nano-extract orally for another 21 days.  **14. Collection and preparation of samples**  At end of the experiment, the overnight fasting rats will be anesthetized through slight exposure to diethyl ether and the blood samples will be drawn from retro-orbital plexus and divided into two parts, part I: will be deposited in heparinized vacuum tubes (Becton Dickinson, New York, NJ) for the hematological measurements. Part II of the blood samples: will be allowed for clotting at room temperature and then centrifuged at 3000 rpm for 15 minutes; the serum will be separated and kept in clean stoppered vials at -20 °C until the biochemical assay. After sacrificing the animals by cervical dislocation, the liver and brain tissues will be excised and washed in ice-cold saline. A small portions will be autopsied from those tissues then immediately preserved in 10% neutral buffered formalin solution for histopathological investigation and another portion will be homogenized in potassium phosphate buffer (pH 7.4) using Tissue Master TM125 (Omni International, USA). The tissue homogenates will be centrifuged at 3000 rpm for 10 min. and the clear supernatants will be stored at -80 ^o^C to be used for biochemical assays. The last part of tissues will be rapidly frozen with liquid nitrogen for electrophoretic analysis.  **15. Hematological Measurements**  The blood will be analyzed using an automatic blood analyzer (ABX Micros 60 manufactured by HORIBA ABX SAS) to quantify hemoglobin (HB), red blood cells (RBCs), hematocrit (HCT), corpuscular volume (MCV), mean corpuscular haemoglobin (MCH), mean corpuscular haemoglobin concentration (MCHC), platelets, white blood cells (WBCs) and differential blood cells (lymphocytes, neutrophils, monocytes and eosinophils).  **16. Biochemical Measurements**  All the traditional biochemical parameters were estimated spectrophotometrically in serum samples by colorimetric methods following instruction of the commercial kits.  16.1. Liver functions  Activities of liver enzymes (ALT and AST) will be determined by method based on the principle documented by **Rietman and Frankle (1957)**. Also, alkaline phosphatase (ALP) activity **(Kochmar and Moss, 1976)** and concentration of total bilirubin **(Henry, 1974)** will be determined.  16.2. Protein Profile  Protein profile includes total protein and albumin. The total protein and albumin will be measured in the serum by Biuret colorimetric endpoint method **(Koller, 1984)** and bromocresol green binding assay **(Doumas *et al*., 1971)**, respectively.  16.3. Renal Functions Test  The renal functions will be represented by levels of urea and creatinine. Urea will be measured by urease-colorimetric method based on the Fenton reaction with the Diazinechromogen formed being absorbed strongly at 540 nm according to the procedure suggested by **Patton and Crouch, (1977)**. Creatinine will be measured by method based on colorimetric alkaline picrate method with creatinine-picrate complex measured at 492 nm **(Bowers and Wong, 1980)**.  16.4. Lipid Profile  The lipid profile will be represented mainly by cholesterol and triglycerides (T.Gs). Cholesterol will be measured in the serum by CHOD-PAP-enzymatic colorimetric method according to **Thomas (1992)**. Level of T.Gs will be measured in the serum by GPO-PAP-enzymatic colorimetric method as suggested by **Fossati, (1982)**. Furthermore, levels of low-density lipoprotein cholesterol (LDL-C) and high-density lipoprotein cholesterol (HDL-C) will be estimated based on method suggested by **Lopes-Virella (1977)**.  16.5. RBCs and Plasma Cholinesterase Activities  Activity of cholinesterase will be determined in blood by Ellman Method suggested by **Ellman *et al*. (1961)** and modified by **Gorun *et al*. (1978)**.  **17. Biochemical Assays in Tissues Homogenates**  17.1. Cholinesterase Activity Assay  Activity of cholinesterase will be determined in clear supernatants by Ellman Method suggested by **Ellman *et al*. (1961)** and modified by **Gorun *et al*. (1978)**.  17.2. Markers of the Oxidative Stress  17.2.1. Lipid Peroxidation Product (LPO)  The LPO will be expressed as malondialdehyde (MDA) which is the end product of lipid peroxidation reaction. It will be determined as thiobarbituric acid reactive substance (TBARs) according to method of **Ohkawa *et al*. (1979)**.  17.2.2. Total Antioxidant Capacity  It will be determined in the tissue homogenates by method suggested by **Koracevic *et al*. (2001)**.  17.2.3. Superoxide Dismutase (SOD) Activity  The SOD activity will be measured spectrophotometrically in the tissue homogenates according to method of **Flohé and Ötting (1971)**.  17.2.4. Catalase Activity  It will be assayed in the tissue homogenates by method suggested by **Aebi (1984)**.  17.2.5. Glutathione Peroxidase Activity  It will be measured in the tissue homogenates according to the method described by **Paglia and Valentine (1967)**.  **18. Statistical Analysis**  All data will be statistically analyzed by one-way analysis of variance test (one-way ANOVA) using the Statistical Package for Social Sciences (SPSS for windows, version 11.0) followed by least significant difference (LSD) test and confirmed by Benferoni test.  **19. Histopathological Examination**  After sacrifice, specimens were autopsied from liver and brain of different groups then studied hispathologically according to method suggested by **Banchroft *et al*. (1996)**. The histopathological changes will be scored according to **Dommels *et al*. (2007)**. A rating score between 0 (no damage) and +++ (maximal damage) will be assigned for each investigated section. Sections from at least five rats will be carefully investigated.  **20. Native Electrophoretic Assays**  20.1. Native Electrophoretic Protein Pattern  The vertical slab polyacrylamide gel electrophoresis (PAGE) will be carried out using Mini-gel electrophoresis (BioRad, USA) **(Laemmli, 1970)** with the modification that samples, gels and running buffers were lacking SDS to determine the relative molecular weight of isolated proteins **(Darwesh *et al*., 2015)**. After completing the electrophoretic run, protein bands will be visualized by staining with Commassie Brilliant Blue G-250. The molecular weight of the separated proteins was estimated in comparison to standard molecular weight markers that specially designed for assay quantitative and size determination.  20.2. Electrophoretic Lipid Moieties of Native Protein Pattern  The native gels will be stained for detecting lipid moiety of native protein with Sudan Black B (SBB) according to method described by **Subramaniam and Chaubal (1990)**. The stained lipoprotein bands will appear as black bands. Relative mobilities (Rf) and band percent (B%) of lipoprotein bands will be determined.  20.3. Electrophoretic Isoenzymes (Zymography)  After electrophoretic run, the native gels will be stained according to their enzyme system at the appropriate substrate and chemical solutions then incubation occurs at room temperature in dark in order to complete staining.  20.3.1. Electrophoretic Esterase Pattern  The native gels will be processed for localization of in-gel esterases activity according to method modified by **Ahmad *et al*. (2012)**. The gel will be stained in reaction mixture containing α, β-naphthyl acetate as substrates along with dye coupler Fast Blue RR at 25 °C in dark. The α-naphthyl acetate will be used as substrate for α-esterases and β-naphthyl acetate will be used as substrate for β-esterases. The α-esterases will appear as dark brown bands and β-esterases will appear as dark pink bands.  20.3.2. Electrophoretic Catalase Pattern  The native gel will be processed for catalase pattern according to method suggested by **Siciliano and Shaw (1976)**. After electrophoretic run, the native gel will be incubated with substrate consisting of hydrogen peroxide solution and will be stained by Pot. Iodide solution. The catalase subunits will appear as yellow bands.  20.3.3. Electrophoretic Peroxidase Pattern  The native gel will be incubated with hydrogen peroxide solution as substrate and stained according to the method suggested by **Rescigno *et al*. (1997)**. The peroxidase subunits will appear as dark brown bands.  20.4. Data Analysis  The polyacrylamide gel plate will be photographed, scanned and then analyzed using Quantity One software (Version 4.6.2).  **21. Molecular Biological Analysis (Gene Expression)**  21.1. Isolation of total RNA  TRIzol® Reagent (cat#15596-026, Invitrogen, Germany) will be used to extract total RNA from different tissues of treated rats according to the manufacturer’s instructions with minor modifications. Total RNA will be treated with 1 unit of RQ1 RNAse-free DNAse (Invitrogen, Germany) to digest DNA residues, re-suspended in DEPC-treated water and quantified photospectrometrically at 260 nm. Purity of total RNA will be assessed by the 260/280 nm ratio which was between 1.8 and 2.1. Additionally, integrity will be assured with ethidium bromide-stain analysis of 28S and 18S bands by formaldehyde-containing agarose gel electrophoresis. Aliquots will be used immediately for reverse transcription (RT), otherwise they will be stored at -80°C.  21.2. Reverse transcription (RT) reaction  The complete Poly(A)+ RNA isolated from tissues of treated rats from all groups will be reverse transcribed into cDNA in a total volume of 20 µl using Revert AidTM First Strand cDNA Synthesis Kit (Fermentas, Germany). An amount of total RNA (5µg) will be used with a master mix (MM). The MM will be consisted of 50 mM MgCl_2_,10x reverse transcription (RT) buffer (50 mMKCl; 10 mM Tris-HCl; pH 8.3), 10 mM of each dNTP, 50 µM oligo-dT primer, 20 IU ribonuclease inhibitor (50 k Da recombinant enzyme to inhibit RNase activity) and 50 IU MuLV reverse transcriptase. The mixture of each sample will be centrifuged for 30 sec at 1000 g and transferred to the thermo cycler. The RT reaction will be carried out at 25°C for 10 min, followed by 1 h at 42°C, and finished with a denaturation step at 99 °C for 5 min. Afterwards the reaction tubes containing RT preparations will be flash-cooled in an ice chamber until being used for cDNA amplification through Real Time polymerase chain reaction (RT-PCR).  21.3. Quantitative Real Time- PCR (qRT-PCR)  Qiagen Cycler will be used to determine the rat's cDNA copy number. PCR reactions will be set up in 25 μL reaction mixtures containing 12.5 μL 1× SYBR® Premix Ex Taq TM (TaKaRa, Biotech. Co. Ltd.), 0.5 μL 0.2 μM sense primer, 0.5 μL 0.2 μM antisense primer, 6.5 μL distilled water, and 5 μL of cDNA template. Each experiment included a distilled water control. The sequences of specific primer of the genes will be used (p53, Bax, Bcl2 & caspase 3). At the end of each q RT-PCR a melting curve analysis will be performed at 95.0°C to check the quality of the used primers (Khalil and Booles, 2011).  **References**  **Aebi, H. (1984)**. Catalase in vitro. Methods Enzymol., 105 : 121-126.  **Ahmad, A., Maheshwari, V., Ahmad, A., Saleem, R. and Ahmad, R. (2012).** Observation of Esterase-Like-Albumin Activity during N'-Nitrosodimethyl amine Induced Hepatic Fibrosis in a Mammalian Model. Macedonian Journal of Medical Sciences, 5(1): 55-61.  **Banchroft, J.D. ; Stevens, A. and Turner, D.R. (1996).** Theory and practice of histological techniques. Fourth Ed. Churchil Livingstone, New York, London, San Francisco, Tokyo.  **Bowers, L.D. and Wong, E.T. (1980).** Kinetic serum creatinine assays. II. A critical evaluation and review. Clin. Chem., 26(5): 555 - 561.  **Brand-Williams, W. ; Cuvelier, M.E. and Berset, C. (1995).** Use of a free radical method to evaluate antioxidant activity. Lebenson Wiss Technol., 28:25-30.  **Darwesh, O.M. ; Moawad, H. ; Barakat, O.S. and Abd El-Rahim, W.M. (2015).** Bioremediation of textile reactive blue azo dye residues using nanobiotechnology approaches. Research Journal of Pharmaceutical Biological and Chemical Sciences, 6(1): 1202-1211.  **Dommels, Y.E.M. ; Butts, C.A. ; Zhu, S. ; Davy, M. ; Martell, S. ; Hedderley, D. ; Barnett, M.P.G. ; McNabb, W.C. and Roy, N.C. (2007).** Characterization of intestinal inflammation and identification of related gene expression changes in mdr1a−/− mice. Genes & nutrition, 2(2): 209-223.  **Doumas, B.T. ; Watson, W.A. and Biggs, H.G. (1971).** Albumin standards and the measurement of serum albumin with bromcresol green. Clin. Chim. Acta., 31(1):87-96.  **Ellman, G.L. ; Courtney, K.D. ; Andres Jr, V. and Featherstone, R.M. (1961).** A New and Rapid Colorimetric Determination of Acetyl-cholinesterase Activity. Biochemical Pharmacology, 7 (2): 88–95.  **Flohé, L. and Ötting, F. (1971).** Superoxide dismutase assays. Methods Enzymol., 105: 93–104.  **Fossati, P. and Principe, L. (1982).** Serum triglycerides determined calorimetrically with an enzyme that produces hydrogen peroxide. Clinical Chem., 28: 2077-2080.  **Gorun, V. ; Proinov, L. ; Baltescu, V. ; Balaban, G. and Barzu, O. (1978).** Modified Ellman Procedure for Assay of Cholinesterase in Crude Enzymatic Preparations. Analytical Biochemistry, 86(1): 324-326.  **Gosselin, R.E. ; Smith, R.P. ; Hodge, H.C. and Jeannet Braddock, J. (1984).** Clinical toxicology of commercial products. Fifth edition. Baltimore, MD: Williams and Wilkins.  **Henry, R.J. ; Cannon, D.C. and Winkelman, W. (1974).** Clinical Chemistry Principales and Techniques, 11^th^ edition Harper and Row, pp 1629.  **Khalil, W.K.B. and Booles H.F. (2011).** Protective Role of Selenium against Over-Expression of Cancer-Related Apoptotic Genes Induced by o-Cresol in Rats. Arh. Hig. Rada. Toksikol., 62: 121-129.  **Kochmar, J.F. and Moss, D.W. (1976)**. Fundamentals of Clinical Chemistry. WB Saunders and Co., Philadelphia, PA., pp: 604.  **Koller, A. and Kaplan, L.A., (1984).** Total serum protein. Clinical Chemistry, Theory, Analysis, and Correlation. St. Louis: Mosby Company, pp.1316-1319.  **Koracevic, D. ; Koracevic, G. ; Djordjevic, V. ; Andrejevic, S. and Cosic, V. (2001).** Method for the measurement of antioxidant activity in human fluids. Journal of Clinical Pathology, 54(5): 356-361.  **Laemmli, U.K. (1970)**. Cleavage of structural proteins during the assembly of the head of Bacteriophage T4. Nature, 227: 680-685.  **Lopes-Virella, M.F. (1977).** Colorimetric determination of low and high density lipoproteins. Clin. Chem., 23: 882-882.  **Meister, R.T. (ed.). (1992).** Farm Chemicals Handbook '92. Meister Publishing Company, Willoughby, OH.  **Mosmann, T. (1983).** Rapid colorimetric assays for cellular growth and survival: Application to proliferation and cytotoxicity assays. J. Immunol. Methods, 65: 55-63.  **Ohkawa, H. ; Ohishi, N. and Yagi, K. (1979).** Assay for lipid peroxides in animal tissues by thiobarbituric acid reaction. Anal. Biochem., 95: 351 - 358.  **Oyaizu, M. (1986).** Studies on product of browning reaction prepared from glucose amine. Japanese Journal of nutrition, 44:307-315.  **Paget, G.E. and Barnes, J.M. (1964).** Toxicity tests. In: Laurance DR, Bacharach AL, editors. Evaluation of Drug Activities: Pharmacometrics, Vol 1. New York: Academic Press: p. 135-65.  **Paglia D.E. and Valentine, W.N. (1967)**. Studies on the Quantitive and Qualitative Charecterization of Erythrocyte Glutathione Peroxidase. The Journal of Laboratory and Clinical Medicine, 70(1): 158-163.  **Patton, C.J. and Crouch, S.R. (1977).** Spectrophotometric and kinetics investigation of the Berthelot reaction for the determination of ammonia. Anal. Chem., 49 (3): 464–469.  **Prieto, P. ; Pineda, M. and Aguilar, M. (1999).** Spectrophotometric quantitation of antioxidant capacity through the formation of a phosphomolybdenum complex: Specific application to the determination of vitamin E. Anal. Biochem., 269: 337-341.  **Raj, J. ; Chandra, M. ; Dogra, T.D. ; Pahuja, M. and Raina, A. (2013).** Determination of median lethal dose of combination of endosulfan and cypermethrin in wistar rat. Toxicol. Int., 20(1):1-5.  **Rescigno, A., Sanjust, E., Montanari, L., Sollai, F., Soddu, G., Rinaldi, A.C., Oliva, S. and Rinaldi, A. (1997).** Detection of laccase, peroxidase, and polyphenol oxidase on a single polyacrylamide gel electrophoresis. Analytical letters, 30(12): 2211-2220.  **Rietman, S. and Frankle, S. (1957)**. A colorimetric method for the determination of serum glutamic oxalacetic and glutamic pyruvic transaminases. Am. J. Clin. Pathol., 28(1):56-63.  **Schutz, K. ;Persike, M. ; Carle, R. and Schieber, A. (2006).** Characterization and quantification of anthocyanins in selected artichoke (Cynarascolymus L.) cultivars by HPLC-DAD-ESI-MSn. Anal.Bioanal. Chem., 384: 1511-1517.  **Siciliano, M.J. and Shaw, C.R. (1976).** Separation and visualization of enzymes on gels. In'Chromatographic and Electrophoretic Techniques. Vol. 2. Zone Electrophoresis'. 4th Edn.(Ed. I. Smith.) pp. 185-209.  **Singleton, V.L. and Rossi, J.A. (1965).** Colorimetry of total phenolics with phosphomolybdicphosphotungstic acid reagents. Am. J. Enol.Vitic., 16 (3): 144 - 158.  **Subramaniam, H.N. and Chaubal, K.A. (1990).** Evaluation of intracellular lipids by standardized staining with a Sudan black B fraction. Journal of biochemical and biophysical methods, 21(1): 9-16.  **Thomass, L. (1992).** Colorimetric Determination of Tcholesterol. Labor and Diagnosis: Clinical Chemistry. 5th Edn., Books Verlagsgeselschaft, Frankfurt, pp: 327.  **Vichai, V. and Kirtikara, K. (2006).** Sulforhodamine B colorimetric assay for cytotoxicity screening. Nature Protocols, 1(3):1112-1116.  **Zhao, P. ; Li, N. and Astruc, D. (2013).** State of the art in gold nanoparticle synthesis. Coord. Chem. Rev., 257(3-4): 638-665. |  |
|  | هل تتوقع حدوث أعراض جانبية مثل ألم أوأذى أو معاناة للحيوان؟  - لا ليس من المتوقع حدوث أي آثار جانبية أو ألم لمجموعات الجرذان التي سوف يتم إجراء التجربة عليها |  |
|  | كيف سيتم التعامل معها أو محاولة تجنبها أو تخفيفها؟  - التجربة قصيرة المدة ولا يوجد هناك متسع من الوقت حتى لو كان هناك آثار جانبية غير متوقعه مدة التجربة القصيرة لا تدع الحيوان يتألم فترة طويلة. |  |
|  | هل سيتم إتباع إجراءات التخلص الآمن من الحيوانات:  - نعم وتم قراءة إرشادات التخلص الآمن من الحيوانات النافقة و المخلفات الطبية و البيولوجية في المرفقات رقم 1 و 2 و 3 وتم إجازة خطوات هذا البحث من الناحية العلمية من قبل اللجنة المختصة بعد التأكد من أن البحث يتوافق مع الميثاق الأخلاقى لرعاية الحيوان، وأن الفائدة المرجوة منه تربو على المخاطر المحتملة من البحث. هذا بالإضافة إلى قراءة دليل المركز القومي للبحوث للتعامل الأخلاقي مع حيوانات التجارب في البحث العلمي (مرفق 4) قبل التوقيع علي هذا الإقرار. |  |

**إسم الباحث الرئيس: وائل محمود كامل أبو الثنا**

**التوقيع:**

**التاريخ: 6/ 11/ 2022**

**إقرار لإجراء بحث على الحيوانات البيطرية أو حيوانات التجارب**

**(الباحث الرئيس)**

أقر أنا الباحث الرئيس/ **وائل محمود كامل أبو الثنا**

لبحث عنوانه:

**تقييم التأثير التحسيني لمستخلص لحاء شجرة الكازارينا بعد دمجها بجسيمات الذهب النانومترية ضد السمية المستحثة بالمبيدات في الجرذان**

بأن أكون حريصا على التعامل الرحيم مع الحيوانات التي يتضمنها البحث خاصة فيما يتعلق بالآتي:

1. تسكين الحيوانات في الأماكن المخصصة لرعاية الحيوان تحت إشراف شخص مدرب ومؤهل للتعامل مع الحيوانات مع توفر التهوية الجيدة و الظروف البيئية المناسبة و التغذية و النظافة و الرعاية البيطرية و خلافه
2. تخدير الحيوانات قبل إجراء عمليات جراحية عليها على أن يقوم بالتخدير و الجراحة طبيب بيطرى أوعالم أو فنى حاصلون على التدريب اللازم
3. الرعاية اللاحقة و إعادة تأهيل الحيوانات بعد إجراء التجارب.
4. التخلص الآمن من الحيوانات المستخدمة فى التجارب في حالة التضحية بها اثناء التجربة و التي قد تعرضت لأضرار بالغة تحت تأثير التخدير و الذين ينطوي شفائهم على ألم أو معاناة مع عدم اللجوء إلى القتل الرحيم إلا في الحالات الآتية:
5. حدوث عجز حركي أو شلل للحيوانات أو فقد لقدراتها على أداء وظائفها الطبيعية أو الإدراكية
6. إذا تعرضت الحيوانات أثناء إجراء التجارب لآلام متكررة أو معاناة أو أضرار بالغة أو سينطوي شفاؤهم على ألم أو معاناة
7. عندما يصبح استمرار حياة حيوانات التجارب مهددة لحياة البشر أو لحياة الحيوانات الأخرى

**المقر بما فيه**

**الإسم: وائل محمود كامل أبو الثنا**

**التوقيع:**

**التاريخ: 6/ 11/ 2022**

**مرفق (1)**

**لجنة السلامة و الصحة المهنية**

**إرشادات التخلص الآمن من الحيوانات النافقة و المخلفات الطبية و البيولوجية**

**في حالة وجود استفسارات** **تليفون لجنة السلامة 2025**

**مرفق (2)**

**لجنة السلامة و الصحة المهنية**

**إرشادات التخلص الآمن من الحيوانات النافقة أو المخلفات الطبية أو البيولوجية أو معالجتها**

**التخلص الآمن من حيوانات التجارب و الملوثة بمواد كيميائية عالية السمية**

1. يجب على متداول ىالمخلفات البيولوجية ارتداء معدات الوقاية الشخصية القياسية **(PPE)** اللازمة لأعمال المعامل، و معطف، و نظارات السلامة، والقفازات، و الأحذية المغلقة .للحماية من الإنبعاثات يجب إستخدام دولاب السلامة الحيوية (Biosafety cabinet).
2. يجب فصل و التخلص من جميع المخلفات بما في ذلك الأنسجة و السوائل البيولوجية و المواد و الحاويات الملوثة و الأدوات الحادة و غيرها من العناصر بشكل صحيح على النحو المفصل فى الجدول (1).
3.
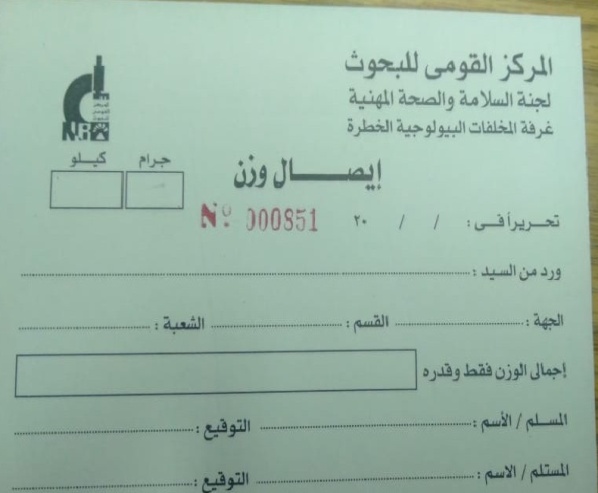
يتم تسليم جميع المخلفات البيولوجية فى غرفة تجميع المخلفات البيولوجية خلف مبنى شعبة النسيج وإستلام إيصال وزن بالتسليم فى خلال 24 ساعة فى الصيف و 48 ساعة فى الشتاء علي النموذج المعد لذلك
4. يتم التخلص من جميع الذبائح و الأنسجة الحيوانية كمخلفات طبية لمنع تلوث حاويات المخلفات الصلبة و لضمان التخلص من الحيوانات المحورة جينيا حيث يجب التخلص منها كمخلفات طبية.
5. يتم مراجعة بروتوكولات استخدام الحيوانات من قبل لجنة أخلاقيات البحوث الطبية بالمركز لتحديد أي ظروف نادرة أو غير عادية من شأنها أن تؤثر على التخلص من المخلفات أو السلامة المهنية.
6. يجب تجميد ذبائح الحيوانات للتخلص منها و وضعها في الكيس الأحمر. و يجب إستخدام كيس مزدوج لمنع الثقوب لحين تسليمها فى غرفة تجميع المخلفات البيولوجية خلف مبنى شعبة النسيج وإستلام إيصال بالتسليم فى خلال 24 ساعة فى الصيف و 48 ساعة فى الشتاء.
7. طبقا لوكالة حماية البيئة **EPA** تعتبر فراش الحيوانات مخلفات صلبة عادية و يتم التخلص منها في حاويات المخلفات الصلبة إلا أنه يجب التعامل معها بعناية لأغراض السلامة و الصحة المهنية لأنه قد يحتوي الفراش الحيواني الناتج عن الحيوانات على طعام أو ماء غير مأكول أو إفرازات تحتوي على كميات صغيرة جدًا من المواد الكيميائية الخطرة، لذا يجب التعامل مع الفراش باستخدام معاطف مختبر كاملة الأزرار و قفازات و نظارات السلامة عند التفريغ فى مكان جيد التهويه مستخدماً قناع الحماية التنفسي أو دولاب سحب الغازات الكيميائى أو كابينة الأمان و السلامة البيولوجية الحيوية. ضع الفراش داخل الكيس، ثم انزع الغطاء، ثم أفرغ الفراش في الكيس الأسود، ثم يجب إغلاق و تأمين الكيس.

**في حالة وجود استفسارات** **تليفون لجنة السلامة 2025**

**مرفق (3)**

**لجنة السلامة و الصحة المهنية**

**إرشادات التخلص الآمن من الحيوانات النافقة أو المخلفات الطبية أو البيولوجية**

**فصل و تجميع المخلفات الطبية أو البيولوجية**

Segregation and Collection of Biological Waste

| Container | Content | Disposal |
| --- | --- | --- |
| Red bag | 1. Infectious waste: Wastes containing infectious agents, such as bacteria, viruses, parasites, and fungi, that could spread the disease   *Examples*: tissues/ swabs/ materials or equipment that have been in contact with infected subject   1. Pathological waste, Tissue or body fluids from humans or animals.   *Examples*: blood, body parts, organs, animal carcasses | These wastes must be kept in red bags, tightly closed and filled more than 3/4, and kept refrigerated till transportation. |
| Special container | Sharp waste: Sharp objects that can easily cut or injure a handler.  *Examples*: syringe needles, scalpels, knives, infusion sets, broken glass | Special containers tightly lidded. |

**في حالة وجود استفسارات** **تليفون لجنة السلامة 2025**
