# Supplementary material for: The hepato- and neuroprotective effect of gold Casuarina equisetifolia bark nano-extract against Chlorpyrifos-induced toxicity in rats
Source: J Genet Eng Biotechnol. 2023 Dec 1;21:158. doi: 10.1186/s43141-023-00595-6 (PMC10692062; doi:10.1186/s43141-023-00595-6)
Supplement: Supplementary file 3 — Additional file 3. [file 43141_2023_595_MOESM3_ESM.doc]

| ***N.B. This unified application form contains FOUR sections, you need to fill only the appropriate section(s) according to the type and design of your study. The basic information as well as the Investigator Commitment (the last page) are mandatory to be filled. To accelerate the review process, it is recommended that you consult with a Research Ethics Advisor (REA) at your institute before submission. You should also check that all data are filled and questions are answered. Any missing data will result in delay in processing your documents to the general meeting which is usually scheduled on the 1^st^ THURSDAY of each month. We should receive the completed application at least before the 16^th^ day the preceding month.***  *MREC Reviewers can write their comments at the right red column or at the end of each section* | | | | | | | |  | |
| --- | --- | --- | --- | --- | --- | --- | --- | --- | --- |
|  | | | | | | | |  | |
| ***Part I: Basic information*** | | | *MERC Reviewer check* | **FOR OFFICE USE ONLY** | | | |  | |
| - 1. **Principal Investigator Data** | | |  |  | | | |  | |
| **PI Name:** Wael Mahmoud Kamel Aboulthana | | | 🞏 Yes 🞏 No | **Date Application Received:** ..…/….../…... | | | |  | |
| **Affiliation:** Biochemistry Department, Biotechnology Research Institute, National Research Centre. | | | 🞏 Yes 🞏 No | **Study Code:** ……………………..…………. | | | |  | |
| **Position:** Assistant Professor | | | 🞏 Yes 🞏 No | **Approval Period:** ………………………… | | | |  | |
| **Phone:** 00201095522103 | | | 🞏 Yes 🞏 No | **From** | | **To** | |  | |
| **Address:** 10 Kamar St., Warrak El Hadder, Giza, Egypt. | | | 🞏 Yes 🞏 No | ……/……/…… | | ……/……/……… | |  | |
| **E-mail:** wmkamel83@hotmail.com. | | | 🞏 Yes 🞏 No |  | |  | |  | |
|  | | |  | *Approve* | *Needs Explanation* | | *Disapprove* | | |
| **Scientific degree and specialty:** | **الدرجة العلمية و التخصص** | | | 🞏 | 🞏 | | 🞏 | | |
| Assistant Professor of Biochemistry | | | |  |  | |  | | |
| **Areas of expertise** | **خبرات الباحث الرئيس في موضوع البحث** | | | 🞏 | 🞏 | | 🞏 | | |
| His scientific work during his M.Sc. and PhD theses, he was concerned with molecular radiation biology through studying effect of gamma irradiation on different body functions at biochemical and molecular levels and hence studying efficiency of willow leaves extract against the adverse effect of gamma irradiation. Moreover, more advanced and deeper studies were carried out through studying effect of gamma irradiation on both of male and female fertility genes and evaluation of the ameliorative of the most abundant phyto-constituent of willow leaves (salicin) against the deleterious effect of irradiation. Recently, he is interesting to evaluate the phytochemical and biological efficiency of different plant extracts then selecting the most effective one. Consequently, the efficiency of the selected extract will be enhanced by incorporating nanoparticles (silver and gold). Then, the nano-extract will be studied against progression of tumors induced chemically in rats as compared to healthy group. Moreover, he is looking forward to use the electrophoresis technique to identify native biomarkers and for detecting the different mutations (qualitative and quantitative) through evaluation of the natured and denatured proteins, different moieties of protein and different isoenzymes. He demonstrated as a personal investigator on the internal projects of the 11th plan (No: 11010344) entitled "Evaluation of Antioxidant Efficiency of Silver Nanoparticles Biosynthesized using *Croton tiglium* L. Seeds Extract against Azoxymethane Induced Colon Cancer in Rats" and the 12th plan (No: E121111) entitled "Evaluation of the ameliorative Effect of *Casuarina equisetifolia* Bark Extract after Incorporating Gold Nanoparticles against Toxicity Induced by Pesticides in Rats" funded by National Research Centre. He published some scientific papers related to this field from his previous work. | | | |  |  | |  | | |
| **Role/tasks in the research/project** | **دور الباحث الرئيس في البحث** | | | 🞏 | 🞏 | | 🞏 | | |
| Suggesting the idea and collected the previously published papers related to it. Also, he is responsible for putting the practical plan and carrying out biochemical analysis as well as molecular assays. Moreover, he will be communicated with journals’ editors asking to publish the manuscript. | | | |  |  | |  | | |
| **Research site and Approval** | **خطاب موافقة الجهات التي سيتم بها البحث** | | | 🞏 | 🞏 | | 🞏 | | |
| …………………………………………………………………………………. | | | |  |  | |  | | |
|  | |  | | | | | | |  |

| **1.2 Protocol Title in English** | | |  | | | *MERC Reviewer check*  Language integrity 🞏 Approve 🞏 Needs Adjustment | | | | | | | | | |
| --- | --- | --- | --- | --- | --- | --- | --- | --- | --- | --- | --- | --- | --- | --- | --- |
| **Evaluation of the ameliorative Effect of *Casuarina equisetifolia* Bark Extract after Incorporating Gold Nanoparticles against Toxicity Induced by Pesticides in Rats** | | | | | | | | | | | | | | | |
| **1.3 Protocol Title in Arabic** | | |  | | | سلامه اللغة في العنوان العربي 🞏 موافق 🞏 يحتاج تعديل | | | | | | | | | |
| **تقييم التأثير التحسيني لمستخلص لحاء شجرة الكازارينا بعد دمجها بجسيمات الذهب النانومترية ضد السمية المستحثة بالمبيدات في الجرذان** | | | | | | | | | | | | | | | |
| **1.3 Study type** | Descriptive studies | | | 🞏 Case- Report | | | 🞏 Case -Serious | | | | | 🞏 Surveys | | |  |
|  | Observational studies | | | 🞏 Cohort Study | | | 🞏 Case – Control | | | | | 🞏 Cross Sectional | | |  |
|  | Interventional Studies | | | 🞏 In Vitro Study | | | 🞏 Using Biological Fluids | | | | | | | |  |
|  |  |  |  |  |  |  | 🞏 Using Cells / Tissues | | | | | | | |  |
|  |  |  |  | 🞏 Clinical Study | | | Using drugs or instruments already approved for such indication | | | | | | | |  |
|  |  |  |  | 🞏 Clinical Trial | | | Using drugs or instruments not approved for such indication or nor registered in Egypt | | | | | | | |  |
|  | Medicinal Plants | | | 🞏 Herbal medicine **🞏 Plant extract** 🞏 | | | | | | | | | | |  |
|  | Others Please specify | | |  | | | | | | | | | | |  |
| **1.4 Anticipated Start Date** | 20 / 09/ 2020 | | | | | | *MERC Reviewer check*  🞏 Yes 🞏 No | | | | | | | |  |
| **1.5 Duration of the study?** | 2 Months | | | | | | 🞏 Yes 🞏 No | | | | | | | |  |
| **1.6 Type of submission** | **🞏 New submission** | | | 🞏 Renewal | | | 🞏 Re-submission | | | | | 🞏 Yes 🞏 No | | |  |
|  | If Re-Submission or Renewal, please provide a copy of the previous approval | | | | | | | | | | | | | |  |
| **1.7 This protocol is for** | | **🞏 Project** | | | 🞏 MD / PhD / MSc | | | | 🞏 Research | | | |  | | |
| **1.8 The protocol was reviewed by scientific reviewing committee** | | **🞏 Yes (Members of Project Office)** | | | | | | | 🞏 No | | | 🞏 Yes 🞏 No | |  | |
| **1.9 Literature Search for Duplication.** This must be within the last 3 months | | 🞏 Pubmed | | | **🞏 Google** | | | | 🞏 Science Direct | | | |  | | |
|  |  | 🞏Others (Please specify)…………… | | | | | | 🞏 Yes 🞏 No | | | | |  | | |
| **1.10 What is the time period covered by the search** | | **🞏 5 years** 🞏 10 years  🞏 Others (If others please specify) | | | | | | 🞏 Yes 🞏 No | | | | |  | | |
| **1.11 Funding Agent** | | 🞏 Self **🞏 Local** 🞏 National 🞏 International  Please specify name of funding agent ………………… | | | | | | | | | Approval from funding agency  موافقة جهة التمويل  🞏 Yes 🞏 No | |  | | |
| **1.12 Fund amount: (60000)** | |  | | | | | | | | | | 🞏 Yes 🞏 No |  | | |
| **1.13** **A contract between the PI and the funding agent** stipulating who possesses the right to publish the results of the study | | **عقد بين الباحث الرئيس و الجهة الممولة للبحث يوضح فيه من المسئول عن نشر النتائج** | | | | | | | | | | 🞏 Yes 🞏 No |  | | |
| **1.13 Keywords in the search and Acronyms or abbreviations used**  Pesticides; *Casuarina equisetifolia* Bark; Green nanotechnology; Gene Expression; Electrophoretic Isoenzymes | | | | | | | | | | 🞏 Yes 🞏 No | | |  | | |

| **Part II: Team Work** |  | | | | | | |  |
| --- | --- | --- | --- | --- | --- | --- | --- | --- |
| **Research Team Information and the process he/ she is responsible for** (Add more lines if necessary) | | | | | | | |  |
| **Co-Investigator 1** | | | | | | | |  |
| Name: Noha El-Sayed Ibrahim | | 🞏 Yes 🞏 No | | | | | |  |
| Affiliation: Microbial Biotechnology Department, Biotechnology Research Institute, National Research Centre | | 🞏 Yes 🞏 No | | | | | |  |
| Position: Assistant Professor | | 🞏 Yes 🞏 No | | | | | |  |
| Phone: 01271974663 | | 🞏 Yes 🞏 No | | | | | |  |
| Email address: nohaelsayed855@gmail.com | | 🞏 Yes 🞏 No | | | | | |  |
| Qualifications and experience: Biochemistry | | 🞏 *Approve* | 🞏 *Needs Explanation* | 🞏 *Disapprove* | | | |  |
| The procedure: he /she is responsible for: She will be responsible for determination of antioxidant activity of the extracts before and after incorporating Au-NPs. | | 🞏 *Approve* | 🞏 *Needs Explanation* | 🞏 *Disapprove* | | | |  |
| **Co-Investigator 2** | | | | | | | |  |
| Name: Esraa Abdel Hamid Refaat | | 🞏 Yes 🞏 No | | | | | |  |
| Affiliation: Pharmacognosy Department, National Research Centre | | 🞏 Yes 🞏 No | | | | | |  |
| Position: Researcher | | 🞏 Yes 🞏 No | | | | | |  |
| Phone: 01000344954 | | 🞏 Yes 🞏 No | | | | | |  |
| Email address: esraa.refaat84@gmail.com | | 🞏 Yes 🞏 No | | | | | |  |
| Qualifications and experience: Pharmacognosy | | 🞏 *Approve* | 🞏 *Needs Explanation* | | 🞏 *Disapprove* | | |  |
| The procedure he /she is responsible for: She will be concerned with undergoing phytochemical screening and preparation of plant extracts. | | 🞏 *Approve* | 🞏 *Needs Explanation* | | 🞏 *Disapprove* | | |  |
| **Co-Investigator 3** | | | | | | | |  |
| Name: Sally Eid Khaled | | 🞏 Yes 🞏 No | | | | | |  |
| Affiliation: Pharmacognosy Department, National Research Centre | | 🞏 Yes 🞏 No | | | | | |  |
| Position: Researcher | | 🞏 Yes 🞏 No | | | | | |  |
| Phone: 01141405050 | | 🞏 Yes 🞏 No | | | | | |  |
| Email address: sasoo_khaled@yahoo.com | | 🞏 Yes 🞏 No | | | | | |  |
| Qualifications and experience: Pharmacognosy | | 🞏 *Approve* | 🞏 *Needs Explanation* | | | 🞏 *Disapprove* | |  |
| The procedure he /she is responsible for: She will be concerned with preparation of plant extracts after Au-NPs incoprporation. | | 🞏 *Approve* | 🞏 *Needs Explanation* | | | 🞏 *Disapprove* | |  |
| **Co-Investigator 4** | | | | | | |  | |
| Name: Ahmed Mahmoud Youssef | | 🞏 Yes 🞏 No | | | | | |  |
| Affiliation: Packaging Materials Department, National Research Centre | | 🞏 Yes 🞏 No | | | | | |  |
| Position: Professor | | 🞏 Yes 🞏 No | | | | | |  |
| Phone: 01270577770 | | 🞏 Yes 🞏 No | | | | | |  |
| Email address: drahmadyoussef1977@gmail.com | | 🞏 Yes 🞏 No | | | | | |  |
| Qualifications and experience: chemistry of Packaging Materials | | 🞏 *Approve* | 🞏 *Needs Explanation* | | | 🞏 *Disapprove* | |  |
| The procedure he /she is responsible for: He will be concerned with biosynthesis of Au-NPs and preparing plant nano-extracts. | | 🞏 *Approve* | 🞏 *Needs Explanation* | | | 🞏 *Disapprove* | |  |
| **Co-Investigator 5** | | | | | | | |  |
| Name: Wagdy Khalil Bassally | |  |  | | | 🞏 Yes 🞏 No | |  |
| Affiliation: Cell Biology Department, Biotechnology Research Institute, National Research Centre | |  |  | | | 🞏 Yes 🞏 No | |  |
| Position: Professor | |  |  | | | 🞏 Yes 🞏 No | |  |
| Phone: 01227410600 | |  |  | | | 🞏 Yes 🞏 No | |  |
| Email address: wagdykh@yahoo.com | |  |  | | | 🞏 Yes 🞏 No | |  |
| Qualifications and experience: Molecular Biology | | 🞏 *Approve* | 🞏 *Needs Explanation* | | | 🞏 *Disapprove* | |  |
| The procedure he /she is responsible for: He will be responsible for carrying out the molecular assays | | 🞏 *Approve* | 🞏 *Needs Explanation* | | | 🞏 *Disapprove* | |  |
| **Co-Investigator 6** | | | | | | | |  |
| Name: Amgad Kamal Hassan | | 🞏 Yes 🞏 No | | | | | |  |
| Affiliation: Biochemistry Department, Biotechnology Research Institute, National Research Centre | | 🞏 Yes 🞏 No | | | | | |  |
| Position: Assistant Professor | | 🞏 Yes 🞏 No | | | | | |  |
| Phone: 01111777068 | | 🞏 Yes 🞏 No | | | | | |  |
| Email address: sakhkakh@yahoo.co.uk  Qualifications and experience: Biochemistry  The procedure he /she is responsible for: He will be responsible for determination of the different biochemical functions in the clear supernatants | | 🞏 *Approve* | 🞏 *Needs Explanation* | | | 🞏 *Disapprove* | |  |
|  | | 🞏 *Approve* | 🞏 *Needs Explanation* | | | 🞏 *Disapprove* | |  |

| **Co-Investigator 7** | | | |
| --- | --- | --- | --- |
| Name: Hassan Abdel-Gawad Hassan | 🞏 Yes 🞏 No | | |
| Affiliation: Applied Organic Chemistry Department, National Research Centre | 🞏 Yes 🞏 No | | |
| Position: Professor | 🞏 Yes 🞏 No | | |
| Phone: 01063157828 | 🞏 Yes 🞏 No | | |
| Email address: abdelgawadhassan@hotmail.com | 🞏 Yes 🞏 No | | |
| Qualifications and experience: Synthetic Organic Chemistry | 🞏 *Approve* | 🞏 *Needs Explanation* | 🞏 *Disapprove* |
| The procedure he /she is responsible for: He will be concerned with purification and preparation of the insecticides. | 🞏 *Approve* | 🞏 *Needs Explanation* | 🞏 *Disapprove* |
| **Co-Investigator 8** | | | |
| Name: Hamdy Ahmed Taha |  |  | 🞏 Yes 🞏 No |
| Affiliation: Applied Organic Chemistry Department, National Research Centre |  |  | 🞏 Yes 🞏 No |
| Position: Professor |  |  | 🞏 Yes 🞏 No |
| Phone: 01001835536 |  |  | 🞏 Yes 🞏 No |
| Email address: hamdytaha69@yahoo.com |  |  | 🞏 Yes 🞏 No |
| Qualifications and experience: Synthetic Organic Chemistry | 🞏 *Approve* | 🞏 *Needs Explanation* | 🞏 *Disapprove* |
| The procedure he /she is responsible for: He will be responsible for determination of the different sensitive enzymes affected by insecticides in the different targeting tissues in rats. | 🞏 *Approve* | 🞏 *Needs Explanation* | 🞏 *Disapprove* |

| *MREC Reviewer Comment* | *Approve* | *Needs Explanation* | *Disapprove* |
| --- | --- | --- | --- |
| - Completely fulfilled and informative data | 🞏 | 🞏 | 🞏 |
| - Specialties/ qualifications/expertise of members are suitable for commanding the research | 🞏 | 🞏 | 🞏 |
| - Members cover all the research methods and techniques | 🞏 | 🞏 | 🞏 |

| **Part III: Study Information and Overview** | | | | |
| --- | --- | --- | --- | --- |
| 3.1 Subject Area (Choose the most appropriate topic) | | | |  |
| 🞏 Behavior | **🞏 Biochemistry** | 🞏 Biomaterials | 🞏 Yes 🞏 No | |
| 🞏Cell Biology | 🞏 Clinical Sciences | 🞏 Drug Development |  | |
| 🞏 Ecology | 🞏 Genetics | 🞏 Gene manipulation |  | |
| 🞏 Molecular Biology | 🞏 Parasitology | 🞏 Neurobiology |  | |
| 🞏 Pharmacology | 🞏 Physiology | 🞏 Toxicology |  | |
| 🞏 Embryology | 🞏 Immunology | 🞏 Surgery |  | |
| 🞏 Others (Please specify) …………………………………………………………………………………… | | | | |

| **3.2 The objective (s), hypothesis and outcomes of this protocol (Add more lines if necessary)** | | | |
| --- | --- | --- | --- |
| **3.2.1. Primary Objective (s)** | **الهدف الأساسي**🞏 Yes 🞏 No | | |
| - How to use green nanotechnology to enhance efficiency of the plant extract by incorporating gold nanoparticles.  - Utilization of the plant extract after incorporating the nanoparticles for resisting (prevention and treatment) the deleterious effect induced a result of the exposure to the widely distributed chemicals.  - Adding new therapeutic strategy called green nanotechnology by combining the phytochemistry with nanotechnology. | | | |
| **3.2.2 Secondary Objectives** | | | **الأهداف الثانوية** 🞏 Yes 🞏 No |
| - Due to availability of these constituents in the nature, it will be expected that the industries will produce and distribute their products after incorporating M-NPs into the natural extract with low cost. The products consisting of nano-extracts will be taken to resist the deleterious effect induced chemically due to the exposure to the toxic substances.  - It will be expected to publish number of scientific papers in this research field to provide us and colleagues of the researchers with data and knowledge to facilitate extension of the idea for undergoing further researches. | | | |
| **3.3 Hypothesis, Rationale and Scientific Validity** | | | **الفكرة و الإثبات العلمي**  🞏 Approve 🞏 Disapprove  🞏Needs Adjustment |
| - The study will be carried out based on the methods suggested by the previously published papers and the obtained results will be discussed according to the previous studies carried out by previous researchers of same scientific field. The data that will be obtained will reveal possibility of the idea to be carried out. | |  | |
| **3.4 Background** Please provide adequate background with references (not more 200 words) | | | **الخلفية العلمية**🞏 Yes 🞏 No |

Chlorpyrifos, [*O,O*-diethyl-*O*-(3,5,6-trichloro-2-pyridyl) phosphorothionate] (CPF) is the commonly used Organophosphorus (OP) insecticide that utilized extensively throughout the world under different registered trademarks **(Toan *et al*., 2013)**. Ethion *(O,O,O,O*-tetraethyl *S,S*-methylene bis (phosphorodithioate)) was first registered as a nonsystemic insecticide and acaricide for use on a wide variety of fruits and vegetables in the United States in 1965 **(EPA, 1989)**.

*Casuarina equisetifolia* bark contained various active phytoconstituents including carbohydrates, alkaloids, proteins, glycosides, saponins, phenolics, flavonoids, tannins, steroids, gum, reducing sugars and triterpenoids **(Al-Snafi, 2015)**. Most of the biologically active components absorbed slowly due to their high molecular weights and this decreases their ability to cross the cellular membrane and hence decreases their efficacy and bioavailability **(Mamillapalli *et al*., 2016)**. Development of nano-extracts containing metal nanoparticles (M-NPs) is considered to be one of the most promising solutions to their inherent stability problem **(Rozenberg and Tenne, 2008)**. The study was designed to select the most effective plant extract to be incorporated by gold nanoparticles (Au-NPs) and hence to investigate its ameliorative effect against the adverse effect induced by OP pesticides in rats.

| **3.5 Sample size and groups** |  | **عدد المشاركين في البحث و المجموعات المختلفة** | | 🞏Yes 🞏 No | |
| --- | --- | --- | --- | --- | --- |
| **Explain how did you calculate the sample size?** | | **كيفية حساب أعداد المشاركين في البحث** | | 🞏Yes 🞏 No | |
| **🞏 Study Power** | | 🞏 Reference | 🞏 Pilot Study | 🞏 Other | |
| If other (please specify) | | | | | |
| **3.6 Procedure in Details with Gantt and Flowcharts:** | | | | | 🞏Yes 🞏 No |

**1. Preliminary Phytochemical Screening Tests**

The total carbohydrates and /or glycosides, free and combined flavonoids, coumarins, saponins, alkaloids, nitrogenous compounds, sterols and /or triterpenes, tannins, proteins and anthraquinones will be determined in *Casuarina equisetifolia* bark.

**2. Yields, physical and chemical characters will be determined in the crude alcoholic, petroleum ether and aqueous extracts**

**3. Determination of total phenolic compounds**

The total polyphenols will be estimated using Folin-Ciocalteu reagent according to method suggested by **Singleton and Rossi (1965)**.

**4. Investigation of the lipoidal matter**

4.1. The total steroidal, terpenoidal and free fatty acids contents will be quantitatively estimated.

4.2. The free fatty acids will be converted into the methylated ester form then analyzed by gas chromatography / mass spectrometer (GC/MS) technique.

4.3. The major steroidal and/or terpenoidal compounds will be isolated from the petroleum ether extract then identified using different spectrophotometric techniques.

**5. Chromatographic and spectrophotometric analysis of the isolated compounds**

Due to the high solubility (in water and/or organic solvents), the HPLC can offer a suitable tool for qualitative and quantitative analysis. Modern detection methods, such as mass spectrometry (MS) and nuclear magnetic resonance (NMR), will be combined with HPLC **(Schutz *et al*., 2006)**, allowing rapid structural analysis and identification of compounds with minimal manipulation of the sample.

**6. Preparation of sample extract for Au-NPs biosynthesis**

The *C. equisetifolia* bark will be weighed, thoroughly washed by double deionised water to remove surface impurities. It will be crushed using a blender and finely macerated. After homogenization, 100ml double deionised water will be added and heated over water bath maintained at 80 °C for 15 minutes. The extract obtained will be filtered through *Whatmann No*.1 filter paper (pore size 25 and used immediately for the biosynthesis of nanoparicles).

**7. *In vitro* antioxidant and cytotoxic activities of the different extracts**

All these measurements will be assayed in the different plant extracts. Also, these assays will be carried out in the extracts before and after incorporating nanoparticles.

7.1. Total polyphenolic compounds

Concentration of the total polyphenols will be estimated in the different plant extracts and nano-extracts by folin ciocalteu reagent using gallic acid as standard according to method described by **Singleton and Rossi (1965)**.

7.2. Total antioxidant capacity

The total antioxidant capacity of extracts and nano-extracts will be evaluated through the assay of the green phosphate/Mo^5+^ complex according to the method described by **Prieto *et al*. (1999)**.

7.3. Total reducing power

The total reducing power will be determined according to method suggested by **Oyaizu (1986)**.

7.4. Free radical scavenging activity

The scavenging activities will be determined against the free radicals initiated by1,1-Diphenyl-2-picryl-hydrazyl (DPPH) based on the method described by **Brand-Williams *et al*. (1995)**.

7.5. Anticancer activity

Cytotoxic activity test (*In vitro* bioassay on human tumor cell lines) will be conducted and determined. It will be performed on human hepatocellular carcinoma cell line (HepG2) based on the method suggested by **Mosmann (1983)** and human colon carcinoma cell line according to protocol suggested by **Vichai and Kirtikara (2006)**. All the tumor cells will be purchased from CSIR-National Chemical Laboratory, Pune, India.

**8. Synthesis of gold nanoparticles (Au-NPs)**

Based on the in vitro antioxidants (total polyphenolic compounds, total antioxidant capacity, total reducing power and scavenging activities against free radicals initiated by DPPH in addition to the cytotoxic activity against growth of human liver and colon carcinoma) that will be assayed in *C. equisetifolia* bark, the most effective plant extracts will be selected to be incorporated by Au-NPs.

8.1. Preparation of gold nanoparticles (Au-NPs)

Generally, the fabrication of Au-NPs via the chemical reduction method will be carried out through two main parts: (I) the first part will be concerned with reduction of Au^+3^ (HAuCl_4_) to Au^0^. For this purpose, the reduction reaction between tetrachloroauric acid (HAuCl) and trisodium citrate (Na_3_C_6_H_5_O_7_.2H_2_O) will be used in an aqueous solution. The second part will be the stabilization using cetyltrimethylammonium bromide (CTAB) in order to avoid aggregation of the particles **(Zhao *et al*., 2013)**.

8.2. Preparation of cellulose nanocrystal (CNC)

A desired amount of dried rice straw powder will be weighed and transferred into a round bottom flask. Alkali solution (4-wt % NaOH) will be added and the treatment will be performed at reflux condition at 100-120ºC for 2 h. The mixture will be then filtered and washed with distilled water several times to remove lignin and hemicellulose that dissolved in the solution. The resulting fiber will be dried before used for bleaching treatment. The bleaching treatment will be performed at reflux condition at 110-130 ºC for 4 hours after adding 30 g of fiber into 200 ml of each solution of 1.7% NaClO_2_, acetic buffer and distilled water. The mixture will be then allowed to cool before filtered and washed with distilled water until white cellulose will be obtained. The cellulose obtained will be dried by using freeze dryer (Labconco) at -39ºC for 24 h. Cellulose nanocrystal will be prepared by using sulfuric acid hydrolysis. A 65 wt% H_2_SO_4_ will be prepared before approximately 5% of cellulose fiber will be added to the solution. The time and temperature will be fixed at 45 °C for 45 min in order to achieve the optimum yield. The hydrolyzed cellulose sample will be washed five times by centrifugation (10,000 rpm, 10 min) to remove excess sulphuric acid. The suspension will be then dialyzed against distilled water until a constant pH will be achieved. The resultant cellulose nanocrystal (CNC) suspension will be stored in refrigerator until further used.

8.3. Preparation of gold nano-extract

Nanoemulsion of the plant extracts will be prepared using crude plant extracts, non-ionic surfactant Tween 20 (HLB-16.7), crystline nanocellulose (CNC) and water via spontaneous emulsification method. Nanoemulsion will be carried out in two steps: in the first step, organic phase will be fabricated through mixing plant crude sample with the chosen surfactant (Tween20) in the following ratio (1:5) then 3 gm of CNC will be added and the mixture will be sonicated for 30 min. In the second stage, the organic phase (Plant extract, Tween 20 and CNC) will be added drop by drop (20 ml/min) to water using separating fennel and stirring the system magnetically (800 rpm) at 60 °C for 5 hrs. Then the prepared gold nanoparticles (Au-NPs) will be added to the prepared nanoemulsion by the following ratio (1%) the mixture will be sonicated to another 30 min at 50 °C.

**9. Characterization of biosynthesized nanoparticles**

9.1. X-ray Diffraction (XRD) Studies

The crystal structure of the filler powders will be determined using a Philips X-ray diffractometer (PW 1930 generator, PW 1820 goniometer) equipped with Cu Kα radiation (45 kV, 40 mA, with λ = 0.15418 nm ). The scans of the analysis will run in 2θ range of 5 to 80º with step size of 0.02 and step time of 1s.

9.2. Ultraviolet-Visible (UV-VIS) Spectroscopy

Synthesis of the nanoparticles using plant extracts will be monitored by measuring UV-VIS spectrum of the reaction mixture at λ 200 - 800 nm after 10-fold dilution of the samples with deionised water. The UV-spectroscopy will be carried by Shimadzu UV-Vis recording spectrophotometer UV-240.

9.3. Transmission Electron Microscope (TEM)

This technique will be employed to visualize size and shape of nanoparticles at high resolution level (200 KV). The morphological and particles size of prepared samples will be demonstrated by using TEM model JEM-1230, Japan, operated at 120 kV, with maximum magnification of 600X10^3^ and a resolution until 0.2 nm. A drop of an aqueous dispersion of the prepared samples will be placed on a carbon-coated copper grid and allowed to dry in air before characterization.

9.4. Dynamic Light Scattering (DLS) measurements

Distribution of the synthesized nanoparticles will be measured by photon correlation spectroscopy (PCS) using Malvern Zetasizer Nano ZS (Malvern Instruments Ltd., Malvern, United Kingdom). Samples will be diluted with double distilled water prior to analysis at room temperature with an angle of detection of 90°.

9.5. Fourier Transform Infrared Spectroscopy (FT-IR) analysis

The FT-IR analysis will be carried out by using FT-IR technique manufactured by Bruker depending on the standard method documented by **Eaton *et al*. (1995)**.

**10. Median lethal dose of different extracts (LD_50_)**

The most effective extract (after incorporating Au-NPs) will be studied separately for evaluating the LD_50_. One hundred and sixty adult albino mice (weight 20-25 g) will be divided into 10 groups (8 mice in each group) for calculating the LD_50_ of extract and 10 groups for that of nano-extracts. The groups will be treated orally by stomach tube with rising the doses. Mortality will be recorded after 24 hrs of extract and nano-extract treatment. The LD_50_ will be calculated using equation suggested by **Paget and Barnes (1974)**.

**11. Administration of plant nano-extracts**

The gold nano-extract will be administrated daily by stomach tube at the suitable safe dose taking in the consideration the difference in weight of the animals. According to the therapeutic window, the dose 1/20 of LD_50_ is the most effective and less toxic. Therefore, it will be selected for the therapeutic purposes.

**12. Induction of toxicity by insecticides**

The animals will be fasted for 18 hrs prior to dosing. The insecticides (chloropyrifos ethyl and ethion) that selected to be under study will be administered orally to rats using stomach tube. Volume of the dose will depend on weight of the animals. These compounds will be dissolved in dimethyl sulfoxide (DMSO) **(Raj *et al*., 2013)**. The dose recommended to induce toxicity is 1/20 of LD_50_ of the insecticide used. It was found that the LD_50_ of chloropyrifos ethyl was about 229 mg/kg bw for rats **(Gosselin *et al*., 1984)**. Therefore, the dose that will be administrated will be 22.9 mg/kg bw or 11.45 mg/kg bw. As regard to ethion, it was found that the LD_50_ was about 208 mg/kg bw for rats **(Meister, 1992)**. Therefore, the dose that will be administrated will be 20.8 mg/kg bw or 10.40 mg/kg bw.

**13. Experimental design**

Eighty adult male Wistar rats (weighting 120 - 150 g) will be housed in eight cages (ten per cage). The animals will be provided with water *ad libitum* and standard food and maintained under normal environmental conditions at 25 ± 2 °C. The experimental procedures will be carried out according to the ethical protocol and guidelines approved by the institutional animal care of National Research Centre, Dokki, Giza, Egypt. The rats will be randomly divided into eight groups as the following:

Control group (G1): Rats will be fed with normal diet as *ad libitum* and received distilled water in parallel with DMSO for 28 days.

Gold *C. equisetifolia* bark nano-extract treated group (G2): Rats will be fed with normal diet associated with the treatment with gold *C. equisetifolia* bark nano-extract orally at a dose of 1/20 of LD_50_ for 21 days.

Toxic chloropyrifos ethyl treated group (G3): Rats will be treated orally with chloropyrifos ethyl at a dose of 1/20 of LD_50_ for 28 days.

Toxicity induced by chloropyrifos ethyl and gold nano-extract simult.-treated group (G4): Rats will be treated with chloropyrifos ethyl for 28 days and simultaneously treated by gold *C. equisetifolia* bark nano-extract for 21 days.

Toxicity induced by chloropyrifos ethyl and gold nano-extract post-treated group (G5): Rats will be treated with chloropyrifos ethyl for 28 days followed by the treatment with gold *C. equisetifolia* bark nano-extract orally for another 21 days.

Toxic ethion treated group (G6): Rats will be injected with ethion at a dose of 1/20 of LD_50_ for 28 days.

Toxicity induced by ethion and gold nano-extract simult.-treated group (G7): Rats will be treated with ethion for 28 days and simultaneously treated by gold *C. equisetifolia* bark nano-extract for 21 days.

Toxicity induced by ethion and gold nano-extract post-treated group (G8): Rats will be treated with ethion for 28 days followed by the treatment with gold *C. equisetifolia* bark nano-extract orally for another 21 days.

**14. Collection and preparation of samples**

At end of the experiment, the overnight fasting rats will be anesthetized through slight exposure to diethyl ether and the blood samples will be drawn from retro-orbital plexus and divided into two parts, part I: will be deposited in heparinized vacuum tubes (Becton Dickinson, New York, NJ) for the hematological measurements. Part II of the blood samples: will be allowed for clotting at room temperature and then centrifuged at 3000 rpm for 15 minutes; the serum will be separated and kept in clean stoppered vials at -20 °C until the biochemical assay. After sacrificing the animals by cervical dislocation, the liver and brain tissues will be excised and washed in ice-cold saline. A small portions will be autopsied from those tissues then immediately preserved in 10% neutral buffered formalin solution for histopathological investigation and another portion will be homogenized in potassium phosphate buffer (pH 7.4) using Tissue Master TM125 (Omni International, USA). The tissue homogenates will be centrifuged at 3000 rpm for 10 min. and the clear supernatants will be stored at -80 ^o^C to be used for biochemical assays. The last part of tissues will be rapidly frozen with liquid nitrogen for electrophoretic analysis.

**15. Hematological Measurements**

The blood will be analyzed using an automatic blood analyzer (ABX Micros 60 manufactured by HORIBA ABX SAS) to quantify hemoglobin (HB), red blood cells (RBCs), hematocrit (HCT), corpuscular volume (MCV), mean corpuscular haemoglobin (MCH), mean corpuscular haemoglobin concentration (MCHC), platelets, white blood cells (WBCs) and differential blood cells (lymphocytes, neutrophils, monocytes and eosinophils).

**16. Biochemical Measurements**

All the traditional biochemical parameters were estimated spectrophotometrically in serum samples by colorimetric methods following instruction of the commercial kits.

16.1. Liver functions

Activities of liver enzymes (ALT and AST) will be determined by method based on the principle documented by **Rietman and Frankle (1957)**. Also, alkaline phosphatase (ALP) activity **(Kochmar and Moss, 1976)** and concentration of total bilirubin **(Henry, 1974)** will be determined.

16.2. Protein Profile

Protein profile includes total protein and albumin. The total protein and albumin will be measured in the serum by Biuret colorimetric endpoint method **(Koller, 1984)** and bromocresol green binding assay **(Doumas *et al*., 1971)**, respectively.

16.3. Renal Functions Test

The renal functions will be represented by levels of urea and creatinine. Urea will be measured by urease-colorimetric method based on the Fenton reaction with the Diazinechromogen formed being absorbed strongly at 540 nm according to the procedure suggested by **Patton and Crouch, (1977)**. Creatinine will be measured by method based on colorimetric alkaline picrate method with creatinine-picrate complex measured at 492 nm **(Bowers and Wong, 1980)**.

16.4. Lipid Profile

The lipid profile will be represented mainly by cholesterol and triglycerides (T.Gs). Cholesterol will be measured in the serum by CHOD-PAP-enzymatic colorimetric method according to **Thomas (1992)**. Level of T.Gs will be measured in the serum by GPO-PAP-enzymatic colorimetric method as suggested by **Fossati, (1982)**. Furthermore, levels of low-density lipoprotein cholesterol (LDL-C) and high-density lipoprotein cholesterol (HDL-C) will be estimated based on method suggested by **Lopes-Virella (1977)**.

16.5. RBCs and Plasma Cholinesterase Activities

Activity of cholinesterase will be determined in blood by Ellman Method suggested by **Ellman *et al*. (1961)** and modified by **Gorun *et al*. (1978)**.

**17. Biochemical Assays in Tissues Homogenates**

17.1. Cholinesterase Activity Assay

Activity of cholinesterase will be determined in clear supernatants by Ellman Method suggested by **Ellman *et al*. (1961)** and modified by **Gorun *et al*. (1978)**.

17.2. Markers of the Oxidative Stress

17.2.1. Lipid Peroxidation Product (LPO)

The LPO will be expressed as malondialdehyde (MDA) which is the end product of lipid peroxidation reaction. It will be determined as thiobarbituric acid reactive substance (TBARs) according to method of **Ohkawa *et al*. (1979)**.

17.2.2. Total Antioxidant Capacity

It will be determined in the tissue homogenates by method suggested by **Koracevic *et al*. (2001)**.

17.2.3. Superoxide Dismutase (SOD) Activity

The SOD activity will be measured spectrophotometrically in the tissue homogenates according to method of **Flohé and Ötting (1971)**.

17.2.4. Catalase Activity

It will be assayed in the tissue homogenates by method suggested by **Aebi (1984)**.

17.2.5. Glutathione Peroxidase Activity

It will be measured in the tissue homogenates according to the method described by **Paglia and Valentine (1967)**.

**18. Statistical Analysis**

All data will be statistically analyzed by one-way analysis of variance test (one-way ANOVA) using the Statistical Package for Social Sciences (SPSS for windows, version 11.0) followed by least significant difference (LSD) test and confirmed by Benferoni test.

**19. Histopathological Examination**

After sacrifice, specimens were autopsied from liver and brain of different groups then studied hispathologically according to method suggested by **Banchroft *et al*. (1996)**. The histopathological changes will be scored according to **Dommels *et al*. (2007)**. A rating score between 0 (no damage) and +++ (maximal damage) will be assigned for each investigated section. Sections from at least five rats will be carefully investigated.

**20. Native Electrophoretic Assays**

20.1. Native Electrophoretic Protein Pattern

The vertical slab polyacrylamide gel electrophoresis (PAGE) will be carried out using Mini-gel electrophoresis (BioRad, USA) **(Laemmli, 1970)** with the modification that samples, gels and running buffers were lacking SDS to determine the relative molecular weight of isolated proteins **(Darwesh *et al*., 2015)**. After completing the electrophoretic run, protein bands will be visualized by staining with Commassie Brilliant Blue G-250. The molecular weight of the separated proteins was estimated in comparison to standard molecular weight markers that specially designed for assay quantitative and size determination.

20.2. Electrophoretic Lipid Moieties of Native Protein Pattern

The native gels will be stained for detecting lipid moiety of native protein with Sudan Black B (SBB) according to method described by **Subramaniam and Chaubal (1990)**. The stained lipoprotein bands will appear as black bands. Relative mobilities (Rf) and band percent (B%) of lipoprotein bands will be determined.

20.3. Electrophoretic Isoenzymes (Zymography)

After electrophoretic run, the native gels will be stained according to their enzyme system at the appropriate substrate and chemical solutions then incubation occurs at room temperature in dark in order to complete staining.

20.3.1. Electrophoretic Esterase Pattern

The native gels will be processed for localization of in-gel esterases activity according to method modified by **Ahmad *et al*. (2012)**. The gel will be stained in reaction mixture containing α, β-naphthyl acetate as substrates along with dye coupler Fast Blue RR at 25 °C in dark. The α-naphthyl acetate will be used as substrate for α-esterases and β-naphthyl acetate will be used as substrate for β-esterases. The α-esterases will appear as dark brown bands and β-esterases will appear as dark pink bands.

20.3.2. Electrophoretic Catalase Pattern

The native gel will be processed for catalase pattern according to method suggested by **Siciliano and Shaw (1976)**. After electrophoretic run, the native gel will be incubated with substrate consisting of hydrogen peroxide solution and will be stained by Pot. Iodide solution. The catalase subunits will appear as yellow bands.

20.3.3. Electrophoretic Peroxidase Pattern

The native gel will be incubated with hydrogen peroxide solution as substrate and stained according to the method suggested by **Rescigno *et al*. (1997)**. The peroxidase subunits will appear as dark brown bands.

20.4. Data Analysis

The polyacrylamide gel plate will be photographed, scanned and then analyzed using Quantity One software (Version 4.6.2).

**21. Molecular Biological Analysis (Gene Expression)**

21.1. Isolation of total RNA

TRIzol® Reagent (cat#15596-026, Invitrogen, Germany) will be used to extract total RNA from different tissues of treated rats according to the manufacturer’s instructions with minor modifications. Total RNA will be treated with 1 unit of RQ1 RNAse-free DNAse (Invitrogen, Germany) to digest DNA residues, re-suspended in DEPC-treated water and quantified photospectrometrically at 260 nm. Purity of total RNA will be assessed by the 260/280 nm ratio which was between 1.8 and 2.1. Additionally, integrity will be assured with ethidium bromide-stain analysis of 28S and 18S bands by formaldehyde-containing agarose gel electrophoresis. Aliquots will be used immediately for reverse transcription (RT), otherwise they will be stored at -80°C.

21.2. Reverse transcription (RT) reaction

The complete Poly(A)+ RNA isolated from tissues of treated rats from all groups will be reverse transcribed into cDNA in a total volume of 20 µl using Revert AidTM First Strand cDNA Synthesis Kit (Fermentas, Germany). An amount of total RNA (5µg) will be used with a master mix (MM). The MM will be consisted of 50 mM MgCl_2_,10x reverse transcription (RT) buffer (50 mMKCl; 10 mM Tris-HCl; pH 8.3), 10 mM of each dNTP, 50 µM oligo-dT primer, 20 IU ribonuclease inhibitor (50 k Da recombinant enzyme to inhibit RNase activity) and 50 IU MuLV reverse transcriptase. The mixture of each sample will be centrifuged for 30 sec at 1000 g and transferred to the thermo cycler. The RT reaction will be carried out at 25°C for 10 min, followed by 1 h at 42°C, and finished with a denaturation step at 99 °C for 5 min. Afterwards the reaction tubes containing RT preparations will be flash-cooled in an ice chamber until being used for cDNA amplification through Real Time polymerase chain reaction (RT-PCR).

21.3. Quantitative Real Time- PCR (qRT-PCR)

Qiagen Cycler will be used to determine the rat's cDNA copy number. PCR reactions will be set up in 25 μL reaction mixtures containing 12.5 μL 1× SYBR® Premix Ex Taq TM (TaKaRa, Biotech. Co. Ltd.), 0.5 μL 0.2 μM sense primer, 0.5 μL 0.2 μM antisense primer, 6.5 μL distilled water, and 5 μL of cDNA template. Each experiment included a distilled water control. The sequences of specific primer of the genes will be used (p53, Bax, Bcl2 & caspase 3). At the end of each q RT-PCR a melting curve analysis will be performed at 95.0°C to check the quality of the used primers (Khalil and Booles, 2011).

| **Part IV: Safety Issues** | | | 🞏Yes 🞏 No |
| --- | --- | --- | --- |
| 4.1 Does this protocol involve the use of substances that may pose any health risk (infectious, carcinogenic, toxic, bacteria, viruses, fungi, parasites, cell lines, primary cells, tissue, fluids, blood, recombinant DNA, chemicals, laser or radiation) to humans? | | | |
| 🞏 **NO** | **🞏 YES** | If yes, please indicate the hazards that the agent(s) may pose to humans and/or animals and mention the precautions that will be followed to minimize health risk. | |
| Agent | Method of Administration | How you are going to reduce or mitigate the risks? | |
| Chloropyrifos ethyl | Injecting Chloropyrifos ethyl at a dose of 22.9 mg/kg bw intraperitoneally (*i.p*.) for 28 continuous days | Taking in the consideration the most suitable precautions in the laboratory | |
| Ehion | Injecting Ethion at a dose of 20.8 mg/kg bw intraperitoneally (*i.p*.) for 28 continuous days | Taking in the consideration the most suitable precautions in the laboratory | |

| 4.2 Does this protocol involve the use of substances that may pose any health risk (infectious, carcinogenic or toxic) to humans and/ or animals (e.g. bacteria, viruses, fungi, parasites, cell lines, primary cells, tissue, fluids, blood, recombinant DNA, chemicals, laser or radiation)? **🞏Yes** 🞏 No  If yes, please indicate the hazards that the agent(s) may pose to humans and/or animals and mention the precautions that will be followed to minimize health risk.  Using gloves and musk and all possible precautions during the works with this toxic substance. Following instructions of the biosafety in the laboratory. | | 🞏Yes 🞏 No |
| --- | --- | --- |
| 4.3 Carefully describe the mitigation strategies to protect the researchers and other staff, human subjects from risks of occupational hazards and the intended procedures (biological, chemical, physical, radiological, etc.)?  Spreading the suitable knowledge about this toxic substance. Following instructions of the biosafety in the laboratory. Disposal the remaining waste after finishing the experiment. | | 🞏Yes 🞏 No |
| 4.4 Carefully describe the mitigation strategies to protect the biospecimen from being exposed to spoilage or spreading infection?  Following the necessary laboratory instructions of preservation of the biological specimens away from the contamination of infection. | | 🞏Yes 🞏 No |
| 4.5 Carefully describe the mitigation strategies to protect the environment?  Following the necessary laboratory instructions of disposal of the wastes obtained from the biological specimens. | | 🞏Yes 🞏 No |
|  | | |
|  | | |
| **Part V: List of References** | 🞏Yes 🞏 No | |
| **Aebi, H. (1984)**. Catalase in vitro. Methods Enzymol., 105 : 121-126.  **Ahmad, A., Maheshwari, V., Ahmad, A., Saleem, R. and Ahmad, R. (2012).** Observation of Esterase-Like-Albumin Activity during N'-Nitrosodimethyl amine  **Banchroft, J.D. ; Stevens, A. and Turner, D.R. (1996).** Theory and practice of histological techniques. Fourth Ed. Churchil Livingstone, New York, London, San Francisco, Tokyo.  **Bowers, L.D. and Wong, E.T. (1980).** Kinetic serum creatinine assays. II. A critical evaluation and review. Clin. Chem., 26(5): 555-561.  **Brand-Williams, W. ; Cuvelier, M.E. and Berset, C. (1995).** Use of a free radical method to evaluate antioxidant activity. Lebenson Wiss Technol., 28:25-30.  **Darwesh, O.M. ; Moawad, H. ; Barakat, O.S. and Abd El-Rahim, W.M. (2015).** Bioremediation of textile reactive blue azo dye residues using nanobiotechnology approaches. Research Journal of Pharmaceutical Biological and Chemical Sciences, 6(1): 1202-1211.  **Dommels, Y.E.M. ; Butts, C.A. ; Zhu, S. ; Davy, M. ; Martell, S. ; Hedderley, D. ; Barnett, M.P.G. ; McNabb, W.C. and Roy, N.C. (2007).** Characterization of intestinal inflammation and identification of related gene expression changes in mdr1a−/− mice. Genes & nutrition, 2(2): 209-223.  **Doumas, B.T. ; Watson, W.A. and Biggs, H.G. (1971).** Albumin standards and the measurement of serum albumin with bromcresol green. Clin. Chim. Acta., 31(1):87-96.  **Ellman, G.L. ; Courtney, K.D. ; Andres Jr, V. and Featherstone, R.M. (1961).** A New and Rapid Colorimetric Determination of Acetyl-cholinesterase Activity. Biochemical Pharmacology, 7 (2): 88-95.  **Environmental Protection Agency (EPA). (1989).** Guidance for the Reregistration of Pesticide Products Containing Ethion as the Active Ingredient. Washington, DC: U.S. Environmental Protection Agency.  **Flohé, L. and Ötting, F. (1971).** Superoxide dismutase assays. Methods Enzymol., 105: 93-104.  **Fossati, P. and Principe, L. (1982).** Serum triglycerides determined calorimetrically with an enzyme that produces hydrogen peroxide. Clinical Chem., 28: 2077-2080.  **Gorun, V. ; Proinov, L. ; Baltescu, V. ; Balaban, G. and Barzu, O. (1978).** Modified Ellman Procedure for Assay of Cholinesterase in Crude Enzymatic Preparations. Analytical Biochemistry, 86(1): 324-326.  **Gosselin, R.E. ; Smith, R.P. ; Hodge, H.C. and Jeannet Braddock, J. (1984).** Clinical toxicology of commercial products. Fifth edition. Baltimore, MD: Williams and Wilkins.  **Henry, R.J. ; Cannon, D.C. and Winkelman, W. (1974).** Clinical Chemistry Principales and Techniques, 11^th^ edition Harper and Row, pp 1629.  **Khalil, W.K.B. and Booles H.F. (2011).** Protective Role of Selenium against Over-Expression of Cancer-Related Apoptotic Genes Induced by o-Cresol in Rats. Arh. Hig. Rada. Toksikol., 62: 121-129.  **Kochmar, J.F. and Moss, D.W. (1976)**. Fundamentals of Clinical Chemistry. WB Saunders and Co., Philadelphia, PA., pp: 604.  **Koller, A. and Kaplan, L.A., (1984).** Total serum protein. Clinical Chemistry, Theory, Analysis, and Correlation. St. Louis: Mosby Company, pp.1316-1319.  **Siciliano, M.J. and Shaw, C.R. (1976).** Separation and visualization of enzymes on gels. In'Chromatographic and Electrophoretic Techniques. Vol. 2. Zone Electrophoresis'. 4th Edn.(Ed. I. Smith.) pp. 185-209.  **Singleton, V.L. and Rossi, J.A. (1965).** Colorimetry of total phenolics with phosphomolybdicphosphotungstic acid reagents. Am. J. Enol.Vitic., 16 (3): 144-158.  **Subramaniam, H.N. and Chaubal, K.A. (1990).** Evaluation of intracellular lipids by standardized staining with a Sudan black B fraction. Journal of biochemical and biophysical methods, 21(1): 9-16.  **Thomass, L. (1992).** Colorimetric Determination of Tcholesterol. Labor and Diagnosis: Clinical Chemistry. 5th Edn., Books Verlagsgeselschaft, Frankfurt, pp: 327.  **Toan, P.V. ; Sebesvari, Z. ; Blasing, M. ; Rosendahl, I. and Renaud, F.G. (2013).** Pesticide management and their residues in sediments and surface and drinking water in the Mekong Delta, Vietnam. Sci. Total Environ., 452: 28-39.  **Vichai, V. and Kirtikara, K. (2006).** Sulforhodamine B colorimetric assay for cytotoxicity screening. Nature Protocols, 1(3): 1112-1116.  **Zhao, P. ; Li, N. and Astruc, D. (2013).** State of the art in gold nanoparticle synthesis. Coord. Chem. Rev., 257(3-4): 638-665. | Induced Hepatic Fibrosis in a Mammalian Model. Macedonian Journal of Medical Sciences, 5(1): 55-61.  **Al-Snafi, A.E. (2015).** The pharmacological importance of *Casuarina equisetifolia* - an overview. International Journal of Pharmacological Screening Methods, 5(1): 4-9.  **Koracevic, D. ; Koracevic, G. ; Djordjevic, V. ; Andrejevic, S. and Cosic, V. (2001).** Method for the measurement of antioxidant activity in human fluids. Journal of Clinical Pathology, 54(5): 356-361.  **Laemmli, U.K. (1970)**. Cleavage of structural proteins during the assembly of the head of Bacteriophage T4. Nature, 227: 680-685.  **Lopes-Virella, M.F. (1977).** Colorimetric determination of low and high density lipoproteins. Clin. Chem., 23: 882-882.  **Mamillapalli, V. ; Atmakuri, A.M. and Khantamneni, P. (2016).** Nanoparticles for Herbal Extracts, Asian Journal of Pharmaceutics, 10(2): S54-S60.  **Meister, R.T. (ed.). (1992).** Farm Chemicals Handbook '92. Meister Publishing Company, Willoughby, OH.  **Mosmann, T. (1983).** Rapid colorimetric assays for cellular growth and survival: Application to proliferation and cytotoxicity assays. J. Immunol. Methods, 65: 55-63.  **Ohkawa, H. ; Ohishi, N. and Yagi, K. (1979).** Assay for lipid peroxides in animal tissues by thiobarbituric acid reaction. Anal. Biochem., 95: 351-358.  **Oyaizu, M. (1986).** Studies on product of browning reaction prepared from glucose amine. Japanese Journal of nutrition, 44: 307-315.  **Paget, G.E. and Barnes, J.M. (1964).** Toxicity tests. In: Laurance DR, Bacharach AL, editors. Evaluation of Drug Activities: Pharmacometrics, Vol 1. New York: Academic Press: p. 135-65.  **Paglia D.E. and Valentine, W.N. (1967)**. Studies on the Quantitive and Qualitative Charecterization of Erythrocyte Glutathione Peroxidase. The Journal of Laboratory and Clinical Medicine, 70(1): 158-163.  **Patton, C.J. and Crouch, S.R. (1977).** Spectrophotometric and kinetics investigation of the Berthelot reaction for the determination of ammonia. Anal. Chem., 49 (3): 464-469.  **Prieto, P. ; Pineda, M. and Aguilar, M. (1999).** Spectrophotometric quantitation of antioxidant capacity through the formation of a phosphomolybdenum complex: Specific application to the determination of vitamin E. Anal. Biochem., 269: 337-341.  **Raj, J. ; Chandra, M. ; Dogra, T.D. ; Pahuja, M. and Raina, A. (2013).** Determination of median lethal dose of combination of endosulfan and cypermethrin in wistar rat. Toxicol. Int., 20(1): 1-5.  **Rescigno, A., Sanjust, E., Montanari, L., Sollai, F., Soddu, G., Rinaldi, A.C., Oliva, S. and Rinaldi, A. (1997).** Detection of laccase, peroxidase, and polyphenol oxidase on a single polyacrylamide gel electrophoresis. Analytical letters, 30(12): 2211-2220.  **Rietman, S. and Frankle, S. (1957)**. A colorimetric method for the determination of serum glutamic oxalacetic and glutamic pyruvic transaminases. Am. J. Clin. Pathol., 28(1): 56-63.  **Rozenberg, B.A. and Tenne, R. (2008).** Polymer-assisted fabrication of nanoparticles and nanocomposites. Progress in Polymer Science, 33 (1): 40-112.  **Schutz, K. ;Persike, M. ; Carle, R. and Schieber, A. (2006).** Characterization and quantification of anthocyanins in selected artichoke (Cynarascolymus L.) cultivars by HPLC-DAD-ESI-MSn. Anal.Bioanal. Chem., 384: 1511-1517. | |

| *MREC Reviewer Comment* | *Approve* | *Needs Explanation* | *Disapprove* |
| --- | --- | --- | --- |
| Completely fulfilled and informative data | 🞏 | 🞏 | 🞏 |
| Comments to the applicant |  |  |  |
| Comment to the Committee |  |  |  |

| **Section (A): Application for Clinical Research Studies Involving Humans** | | | |
| --- | --- | --- | --- |
| 1. **Does your research involve any vulnerable groups:** 🞏 **Yes** 🞏 **No**   If yes explain why?........................................................................................................................................................................  ***N.B: Vulnerable groups as****: Children or young people < 18 years, Occupational groups, Staff at your work, members or employees of a certain community or work groups , Prisoners, Addiction groups, People with genetic, metabolic, and hormonal disorders, Athletes, People with a specific health condition, Health service providers, Pregnant women and/or human fetus, Families for familial and hereditary diseases, People who have a sight, hearing, or speech impairment and/or Communication disorders, People with high dependence on medical care who may be unable to give consent, e.g. coma or severely ill, People with a cognitive impairment, an intellectual disability, elderly, or a mental illness, Patients of physiotherapy units, rehabilitation centers* | | |  |
| 1. **Age range of participants** |  |  |  |
| 1. **Locality of recruited population** |  |  |  |
| 1. **Recruitment procedure** | **طريقة تجميع المشاركين** |  |  |
| **Inclusion Criteria** | **Exclusion criteria** |  |  |
| 1- | 1- |  |  |
| 2- | 2- |  |  |
| 3- | 3- |  |  |
| 4- | 4- |  |  |
| 5- | 5- |  |  |
| 6- | 6- |  |  |
| 7- | 7- |  |  |
| 1. **Criteria for study discontinuation** | معايير التوقف عن الدراسة | 🞏Yes 🞏 No |  |
| 1. **Clinical and laboratory tests needed** | الفحوص الإكلينيكية والمعملية المطلوبة | 🞏Yes 🞏 No |  |

| 1. **Drug Data** | معلومات دوائية | 🞏Yes 🞏 No |
| --- | --- | --- |
| 7.1 Toxicological and Pharmacological data | المعلومات السمية و الدوائية | 🞏Yes 🞏 No |
| 7.2 Intended dosages of the of drugs (devices) to be used | جرعات الأدوية أو الأجهزة التي سوف تستخدم | 🞏Yes 🞏 No |
| 7.3 Method of treatment administration | طرق إعطاء العلاج | 🞏Yes 🞏 No |
| 7.4 Planned duration of treatment | المدة المقررة للعلاج | 🞏Yes 🞏 No |
| 7.5 Risks / side effects to the participants | المخاطر/ الأعراض الجانبية للمشتركين | 🞏Yes 🞏 No |
| 7.6 Benefits to the participants | المنافع للمشتركين أو المتطوعين | 🞏Yes 🞏 No |
| 7.7 Methods of recording and reporting adverse events or reactions | طرق إعداد التقارير وتسجيل ردود الفعل السلبية | 🞏Yes 🞏 No |
| 7.8 Provisions for dealing with complications | كيفية الاستعداد لمعالجة المضاعفات | 🞏Yes 🞏 No |
| 7.9 Adequacy of the research site to deal with complications | كفاءة الإجراءات و الوسائل في مكان الدراسة | 🞏Yes 🞏 No |
| 1. **Clinical and laboratory tests needed** | الفحوص الإكلينيكية والمعملية المطلوبة | 🞏Yes 🞏 No |

| 1. **How you are going to protect the confidentiality of data?** | حماية سرية البيانات | 🞏Yes 🞏 No |
| --- | --- | --- |
| Plans and procedures for communicating with subjects about information arising from the study that could affect subjects’ willingness to continue in the study:  🞏 Coding 🞏 Limiting access to information 🞏 Others, please specify | الأشخاص المسئولين والخطط والإجراءات للاتصال بالمشتركين و اطلاعهم على المعلومات الناتجة عن البحث وعن الضرر أو المنفعة، أو غيرها من الأبحاث حول الموضوع نفسه، والتى يمكن أن تؤثرعلى رغبته في المواصلة فى الدراسة | 🞏Yes 🞏 No |
| 1. **Will the information be disclosed to third parties** (e.g., audits, reportable conditions, other legal requirements, others)? 🞏 Yes 🞏 No 🞏 N/A | هل سيتم إطلاع أفراد أو جهات أخري على بيانات المبحوثين؟ | 🞏Yes 🞏 No |
| 1. **The research will have a direct or potential benefit, in the present or future, for the population** | البحث سيكون له منفعة مباشرة أو محتملة في الحاضر أو المستقبل على المجتمع | 🞏Yes 🞏 No |
| 1. **Negative outcome:** an assurance that the results will be made available, as appropriate, through publication or by reporting to local ethics committee and the Egyptian Drug Administration | في حالة حدوث نتائج سلبية، التأكيد على أنها ستعلن، من خلال نشر التقرير و إبلاغ لجنة الأخلاقيات و هيئة الدواء المصرية | 🞏Yes 🞏 No |
| 1. **the Principal Investigator has signed a statement**, that the research will be conducted in conformity with the Declaration of Helsinki (2013) | الباحث الرئيس وقع- على أن البحث سيتم إجرائه وفقا لإعلان هلسنكي (2013) | 🞏Yes 🞏 No |

| *MREC Reviewer Comment* | *Approve* | *Needs Explanation* | *Disapprove* |
| --- | --- | --- | --- |
| Completely fulfilled and informative data | 🞏 | 🞏 | 🞏 |
| Comments to the applicant |  |  |  |
| Comment to the Committee |  |  |  |

**Section (B): Application for Research Studies Involving Animals**

| - ***Category*** | | | **🞏 Research** 🞏 Diagnostic 🞏 Product development  🞏 Training (state course name): ……………………… | | | | | |
| --- | --- | --- | --- | --- | --- | --- | --- | --- |
|  | | |  | | | | | |
| - ***Social relevance or significance*** | | | | | | | | |
| 🞏 Conservation/Environment 🞏 Veterinary Science **🞏 Basic Biology** 🞏 Medical Science  🞏 Other (please specify……………………………………….) | | | | | | | | |
|  | | | | | | | | |
| - ***Justification of animal use and 3Rs***   The MREC requires “that animals should be used only if the researcher’s best efforts to find an alternative have failed”. The three Rs (Replacement, Reduction and Refinement) are the cornerstone of ethical animal research, and MREC requires the investigators to implement the 3Rs whenever possible upon preparing to use animals for scientific or training purposes. | | | | | | | | |
| **Requested animals** | | | | | | | |  |
| Species /  Common Name | Strain/ Breed | Weight range and/or Age | | Sex  (M, F) | Total  Number | | Source |  |
| Mice  Rats | Adult albino  Wistar albino | 20-25 g  120-150 g | | M  M | 72  80 | | Animal House |  |
| N.B. In case of female animals, please check its reproductive status | | | | | | | |  |
| 🞏 Mature | 🞏 Immature | | | 🞏 Pregnant | | 🞏 Lactating | |  |
|  |  | | |  | |  | |  |
| 1. **Replacement:** It refers to methods that avoid or replace the use of animals | | | | | | | |  |
|  | | | | | | | |  |
| Justification of Animal Use: Mice fof calculation of Median Lethal dose (LD50) and Rats for experimental design (induction of hepatotoxicity and studying the therapeutic effect of the extract) | | | | | | | |  |
|  | | | | | | | |  |
| **Species – Specific Consideration** | | | | | | | |  |
|  | | | | | | | |  |
|  | | | | | | | |  |
| 1. **Reduction:** | | | | | | | |  |
|  | | | | | | | |  |
| **Is this a repetition of a previous study?** | | | | | | | |  |
| **🞏 No** | 🞏 Yes | If Yes, please justify why this needs to be repeated? | | | | | |  |

| **Justification of animal number:** Seventy-two adult albino mice (weight 20-25 g) were divided into 6 groups (6 mice in each group) for calculating the LD_50_ of total methanolic extract and 6 groups for that of gold nano-extract. The groups were treated orally by stomach tube with increasing the doses (2000, 4000, 6000, 8000, 10000 and 12000 mg/Kg). Number of dead mice was recorded after 24 hrs of extract administration.  Eighty adult male Wistar rats (weighting 120 - 150 g) will be housed in eight cages (ten per cage). The animals will be provided with water *ad libitum* and standard food and maintained under normal environmental conditions at 25 ± 2 °C. The experimental procedures will be carried out according to the ethical protocol and guidelines approved by the institutional animal care of National Research Centre, Dokki, Giza, Egypt. The rats will be randomly divided into eight groups as the following: Control group (G1): Rats will be fed with normal diet as *ad libitum* and received distilled water in parallel with DMSO for 28 days. Gold *C. equisetifolia* bark nano-extract treated group (G2): Rats will be fed with normal diet associated with the treatment with gold *C. equisetifolia* bark nano-extract orally at a dose of 1/20 of LD_50_ for 21 days. Toxic chloropyrifos ethyl treated group (G3): Rats will be treated orally with chloropyrifos ethyl at a dose of 1/20 of LD_50_ for 28 days. Toxicity induced by chloropyrifos ethyl and gold nano-extract simult.-treated group (G4): Rats will be treated with chloropyrifos ethyl for 28 days and simultaneously treated by gold *C. equisetifolia* bark nano-extract for 21 days. Toxicity induced by chloropyrifos ethyl and gold nano-extract post-treated group (G5): Rats will be treated with chloropyrifos ethyl for 28 days followed by the treatment with gold *C. equisetifolia* bark nano-extract orally for another 21 days. Toxic ethion treated group (G6): Rats will be injected with ethion at a dose of 1/20 of LD_50_ for 28 days. Toxicity induced by ethion and gold nano-extract simult.-treated group (G7): Rats will be treated with ethion for 28 days and simultaneously treated by gold *C. equisetifolia* bark nano-extract for 21 days. Toxicity induced by ethion and gold nano-extract post-treated group (G8): Rats will be treated with ethion for 28 days followed by the treatment with gold *C. equisetifolia* bark nano-extract orally for another 21 days. | | | | | |
| --- | --- | --- | --- | --- | --- |
| **Sample size calculation** | 🞏 study Power | 🞏 Reference | | 🞏 Pilot Study | 🞏 Other |
| 1. **Refinement:** |  |  | |  |  |
| Have you considered pilot studies? | **🞏 No** | 🞏 Yes | |  |  |
| If Yes please explain: | | | If No please justify: | | |

| **Did three Rs determine any possible reduction?** Statistical methods should be described where possible.  Data will be statistically analyzed by one-way analysis of variance test (one-way ANOVA) followed by Bonferoni test as Post-Hoc and presented in Tables and Figures as mean ± standard error (means ± SEM). The differences at a "P" value of less than 0.05 will be considered statistically significant. The GraphPad Prism-8 program will be used for illustrating data of genes expressions. | | | | | |
| --- | --- | --- | --- | --- | --- |
| *Reduction Alternative Category* | | | | | |
| **Animal Re-Use Strategy** | |  | | | |
| Does this protocol involve the re-use of any animals (more than one procedure applied for unrelated experiments on the same animals and in the same project? | | | | | |
| **🞏 No** |  | 🞏 Yes |  |  |  |
| If yes, please explain: | |  | | | |
| - Animals are healthy and previously unused in an experiment | | | | | |
| - Animals previously used for breeding and that have undergone no invasive Procedures (genotyping is not considered an invasive procedure). | | | | | |
| - Animals transferred from one protocol to another approved protocol for the purpose of immediate euthanasia by an approved method stated in the protocol. | | | | | |
| - Animals that have been used for simple experimental procedures but in which no invasive or painful procedures have been performed (e.g. single blood draw or injection), with appropriate justification, animals that have undergone an invasive procedure may be reused for training of a non-survival procedure under an approved protocol. | | | | | |
|  | | | | | |
|  | | |  | | |
| **Experimental procedures and timelines** | | | | | |
| Describe all procedures on the animals and how often they will be done. Surgery should be described here if applicable as it relates to the study design. Specific details on surgery, anesthesia for surgery, and postoperative care are requested  At end of the experiment, the overnight fasting rats will be anesthetized through slight exposure to diethyl ether and the blood samples will be drawn from retro-orbital plexus and divided into two parts, part I: will be deposited in heparinized vacuum tubes (Becton Dickinson, New York, NJ) for the hematological measurements. Part II of the blood samples: will be allowed for clotting at room temperature and then centrifuged at 3000 rpm for 15 minutes; the serum will be separated and kept in clean stoppered vials at -20 °C until the biochemical assay. After sacrificing the animals by cervical dislocation, the liver and brain tissues will be excised and washed in ice-cold saline. A small portions will be autopsied from those tissues then immediately preserved in 10% neutral buffered formalin solution for histopathological investigation and another portion will be homogenized in potassium phosphate buffer (pH 7.4) using Tissue Master TM125 (Omni International, USA). The tissue homogenates will be centrifuged at 3000 rpm for 10 min. and the clear supernatants will be stored at -80 ^o^C to be used for biochemical assays. The last part of tissues will be rapidly frozen with liquid nitrogen for electrophoretic analysis. | | | | | |
| **Experimental Agents:** these include investigational new drugs, placebos, tumor cells, stem cells, gene markers, tracers, radioisotopes, imaging contrast agents, viruses and other biological agents, etc. | | | | | |
| Species | Drug/Agent | Dose  (mg/kg B Wt.) | Route | Frequency | Duration |
|  |  |  |  |  |  |
| **Collection of biological samples** (blood*, body fluid, tissue, hair, swap, tail clip, etc.). | | | | | |
| sample | Site | Method | Amount (size/ volume) | | Frequency |
|  |  |  |  |  |  |

| **Degree of pain severity:** The principal investigator required to document those alternative procedures that may cause pain or distress to animals have been considered. | |
| --- | --- |
| Based on the experimental design and manipulated procedures in this study. Please check ONLY one box | |
| **The most invasive or potentially painful procedure determines the pain severity level** | |
| 🞏 No pain | Animals being bred, acclimatized, or held for use in teaching, testing, experiments, research, or surgery but not yet used for such purpose  *Example:*  Animals being bred or housed, without any research manipulation, prior to euthanasia or transfer to another protocol  Observation of animal behaviour in the wild without manipulating the animal or it’s environment |

| 🞏 Minimum | Animals are subject to procedures that cause no pain or distress, or only momentary or slight pain or distress and do not require the use of pain‐relieving drugs  *Examples:*  1. Holding or weighing animals in training, outreach or research activities  2. Observation of animal behaviour in the lab  3. Ear punching of rodents  4. Tail snips in mice ≤ 21 days old  5. Peripheral Injections, blood collection or catheter implantation  6. Feed studies, which do not result in clinical health problems  7. Routine agricultural husbandry procedures  8. Live trapping  9. Positive reward training or research.  10.Chemical restraint  11. Research procedures that involve no potential increase in pain or distress on client owned animals that are undergoing clinical procedures (ex: drawing extra blood, choice of antibiotics).  12.Exposure to alterations in environmental conditions (not extreme) with appropriate conditioning and microenvironment  13.Food restriction that reduces the animals weight by no more than 20% of normal age matched controls  14. Approved euthanasia procedures.  15. Euthanasia of breeding animals or unused offspring  16. Exsanguination with anaesthesia  17. Perfusion with anesthesia  18. Unknown genetically engineered phenotype | | | | | | |  |
| --- | --- | --- | --- | --- | --- | --- | --- | --- |
| 🞏 Moderate | Animals subjected to potentially painful or stressful procedures for which they receive appropriate anesthetics, analgesics and/or tranquilizer drugs.  *Examples:*  1. Survival surgery  2. Non‐survival surgical procedures  3. Laparoscopy or needle biopsies  4. Retro‐orbital blood collection  5. Exposure of blood vessels for catheter implantation  6. Induced infections or antibody production  7. Tattooing  8. Exposure of skin to UV light to induce sunburn  9. Tail snips in mice > 21 days old  10. Research procedures that could potentially increase pain or distress (anaesthesia /analgesia studies) on client owned animals that are undergoing clinical procedures.  11. Genetically engineered phenotype that causes pain or distress that will be alleviated | | | | | | |  |
| 🞏 Severe | | | Animals subjected to potentially painful or stressful procedures that are not relieved with anesthetics, analgesics and/or tranquilizer drugs.  *Examples:*  1.Toxicological or microbiological testing, cancer research or infectious disease research that requires continuation after clinical symptoms are evident without medical relief or require death as an endpoint  2.Ocular or skin irritancy testing  3.Food or water deprivation beyond that necessary for ordinary pre‐surgical preparation  4.Application of noxious stimuli such as electrical shock that the animal cannot avoid or escape  5.Any procedures for which needed analgesics, tranquilizers, sedatives, or anaesthetics must be withheld for justifiable study purposes  6.Exposure to extreme environmental conditions  7.Euthanasia by procedures not approved by the AVMA  8.Paralysis or immobilization of a conscious animal  9.Genetically engineered phenotype that causes pain or distress that will not be alleviated | | | | | |
| - **Are less painful or stressful alternative available?** | | | | | | | | |
| 🞏 **No** | | 🞏 **Yes** | | If yes, justify why they are not going to be used? | | | | |
|  | | | | | | | | |
| - **Describe the anticipated pain or distress for animals?** | | | | | | | | |
|  | | | | | | | | |
| - **Describe how pain or distress will be monitored?** | | | | | | | | |
|  | | | | | | | | |
| - **List who will monitor or observe animals?** | | | | | | | | |
|  | | | | | | | | |
| - **Indicate the schedule of monitoring?** | | | | | | | | |
|  | | | | | | | | |
| - **Animals in pain or stress:** Describe the interventions and / or dose, frequency and type of anesthetic or analgesic drugs or tranquilizers if pain or distress occurs. | | | | | | | | |
| Agent/Substance | | Drug | | Dosage | Frequency | Route of Administration | | |
| Analgesic Agent | |  | |  |  |  |  | |
| Tranquilizers | |  | |  |  |  |  | |
| Others | |  | |  |  |  |  | |

| - **Does this protocol involve surgery?** | | | | | |
| --- | --- | --- | --- | --- | --- |
| 🞏 **No** | 🞏 **Yes** | If the answer is Yes, complete the following section and if No, proceed to the following section | | | |
| - **Surgical procedures:** Give details and description of the surgical procedures guidelines and pain management during, and/or after surgical intervention. | | | | | |
|  | | | | | |
| - **Anesthetic, analgesic, antibiotic and other drugs used in pain management.** | | | | | |
| Agent/Substance | Drug | Dosage | Frequency | Route of Administration | |
| Anesthetic Agent |  |  |  |  |  |
| Post-operative Analgesic |  |  |  |  |  |
| Antibiotic |  |  |  |  |  |
| Others |  |  |  |  |  |
|  |  |  |  |  |  |
| - **Important Surgical Consideration** | | | | | |
|  | | | | | |
| - **Location (Room, Building) of surgery** | | | …………………………………………………….. | | |
|  | | |  | | |
| - **Pre-operative Care** | | | Describe any care given to the animals prior to the surgery: [e.g., fasting, sedation, pre-operative physical exam or blood work, etc.]. | | |
| - Describe how the level of anesthesia is assessed to be adequate to begin the procedure? | | | …………………………………………………….. | | |
| - Will animals be allowed to recover from anesthesia? | | | 🞏NO | 🞏 Yes |  |
| If the answer is Yes, will more than one major survival surgery be conducted on each animal? | | | | | |
| 🞏 NO | 🞏 Yes | If the answer with Yes, How many times? ( ……………………… ) | | | |
| - Provide scientific justification for more than one major survival surgery on each animal | | | | | |
|  | | | | | |
| - Aseptic techniques | | | | | |
| Preparation of the surgical space | | | …………………………………………………….. | | |
| Preparation of the surgeon: (surgical scrub of hands, donning surgical attire, sterile gloves, etc.) | | | …………………………………………………….. | | |
| Preparation of the animal: ((clip fur, clean surgical site with antiseptics, use of sterile drapes, application of eye ointment, etc.) | | | …………………………………………………….. | | |
| Sterilization of instruments: Describe how instruments will be sterilized: [e.g., autoclave, glass bead sterilizer, chemical sterilizer, etc.) | | | …………………………………………………….. | | |
| Will instruments be used in multiple animals? If so, describe how sterility will be maintained | | | …………………………………………………….. | | |

| - ***Humane Endpoints***   Some experimental manipulations or phenotype abnormalities can be expected to produce a degree of unavoidable pain, distress or illness in experimental animals. These adverse effects will be minimized or alleviated by choosing the earliest endpoints consistent with the scientific objectives of the research. | | | | | |  |
| --- | --- | --- | --- | --- | --- | --- |
| What is the expected time course of the study? (i.e. How long are animals maintained from the first experimental manipulation until the end of the experiment or planned euthanasia?).  What criteria, appropriate to the species, will trigger the decision to end the study, stop the procedure, or humanely euthanize an animal before the experimental objective is achieved? Examples could include the following: a weight loss limit (not more than 20%) as a percentage of body weight, allowable durations of anorexia, ulcerative skin lesions. | | | | | |  |
| - ***Experimental Procedures*** | | | | | |  |
| *Provide all proposed experiments and different measurements that will be applied on the collected samples.* | | | | | |  |
| - ***Euthanasia*** | | | | | | |
| *This must be answered even in a non-terminal study, where an animal may experience a Humane Endpoint not related to the research i.e. in case of planned or unplanned (emergency) euthanasia. Methods of euthanasia must be listed as acceptable by the most recent Report of the AVMA (American Veterinary Medical Association) Guidelines on Euthanasia (*[*https://www.avma.org/KB/Policies/Documents/euthanasia.pdf*](https://www.avma.org/KB/Policies/Documents/euthanasia.pdf)*).* | | | | | | |
|  | | |  | | |  |
| 🞏 Euthanasia is part of the study design | | | 🞏 Euthanasia is NOT part of the study design | | |  |
| **Species** | **Method** | **Drug** | **Dose (mg/kg)/ For gas use%** | | **Route** |  |
|  |  |  |  |  |  |  |
|  | Anesthetic overdose |  |  |  |  |  |
|  | Decapitation under anesthesia or tranquilization |  |  |  |  |  |
|  | Cervical dislocation (CD) under anesthesia or tranquilization |  |  |  |  |  |
|  | Exsanguination/cardiac perfusion under anesthesia |  |  |  |  |  |
|  | Other method (Please specify) |  |  |  |  |  |
| - **Methods of euthanasia*** N.B: If more than one method is used per species please list all methods. | | | | | |  |
| - Open chest inspection of the heart | | |  |  |  |  |
| - Exsanguination (cutting a major blood vessel) | | |  |  |  |  |
| - Physical method (specify): | | | ……………………………………………………………. | | |  |
| - Other (describe): | | |  |  |  |  |
| If you are using any one of the previous methods of euthanasia without using anesthesia, please provide scientific justification (with references if available) for why anesthesia cannot be used. | | | | | |  |
| - **[Confirmation of Death in Animals](http://www.research.uci.edu/compliance/animalcare-use/research-policies-and-guidance/euthanasia.html#euth4)** | | | | | |  |

- ***Animal Housing***

Animal Housing Requirements (Select and check)

| 1-Specify the intended Animal Housing Facility | |  |  |  |
| --- | --- | --- | --- | --- |
| 2-Animal Facility Supervisor | |  |  |  |
| 3-Microenvironment | Housing | 🞏 Group | 🞏 Individual |  |
|  | Cage type | 🞏 Conventional | 🞏 IVC | 🞏 Micro-isolator |
|  | Bedding | 🞏 Normal | 🞏 Special |  |
|  | Feeding | 🞏 Normal | 🞏 Special diet | 🞏 Special regime |
|  | Watering | 🞏 Normal | 🞏 Supplemented |  |
| 4-Macroenvironment | Temperature | 🞏 Ambient | 🞏 Other (Details……………………,,,,,.) | |
|  | Humidity | 🞏 Ambient | 🞏 Other (Details………………………..) | |
|  | Containment | 🞏 Normal | 🞏 Other (Details…………………….….) | |

- ***Animal disposition***

| 1-If animals are not to be euthanized at the completion of the protocol, please describe their ultimate use. | ……………………………………………………………. |
| --- | --- |
| 2-Identify and explain if any individual animal in this project will be used in any other project | ……………………………………………………………. |
| 3-What will be the method of disposal of dead animals | ……………………………………………………………. |

- ***Technical /Training requests***

Will researchers perform technical procedures on animals in addition to routine husbandry? 🞏 **NO** 🞏 **Yes**

If yes, please fill the following table:

| Procedure | Name of the researcher | Training |
| --- | --- | --- |
| 1 |  |  |
| 2 |  |  |
| 3 |  |  |
| 4 |  |  |
| 5 |  |  |

*Please explain how the researcher was trained to perform this procedure (Certificate, personnel training, video……..……etc.).

| *MREC Reviewer Comment* | *Approve* | *Needs Explanation* | *Disapprove* |
| --- | --- | --- | --- |
| Completely fulfilled and informative data | 🞏 | 🞏 | 🞏 |
| Comments to the applicant |  |  |  |
| Comment to the Committee |  |  |  |

**Part (C): Application for Research Studies using Medicinal Plants
(Wild or Cultivated)**

| 1. **Objectives:** The PI should comment on the following concerns: | | | | *MERC Reviewer check* |
| --- | --- | --- | --- | --- |
| - what is the social value of this research? | | | | 🞏Yes 🞏 No |
|  | | | |
| - what is the scientific validity? | | | | 🞏Yes 🞏 No |
|  | | | |
| - what is the environmental advantage in the future? | | | | 🞏Yes 🞏 No |
|  | | | |
| - what is the economic benefits? | | | | 🞏Yes 🞏 No |
|  | | | |
| - How to conserve the plant, if it is rare and possesses a valuable biological importance? | | | | 🞏Yes 🞏 No |
|  | | | |
| - In case of biotechnological plant researches, you should clarify the difference between the biological activity of natural plant fractions or compounds versus the genetically modified plant? | | | | 🞏Yes 🞏 No |
|  | | | |
| - In the event of important new findings, to whom the Intellectual Property Rights will belong to? | | | | 🞏Yes 🞏 No |
|  | | | |
| 1. **Testing medicinal plants on animals**: The PI should mention the following items | | | |  |
| Acute toxicity | LD50 | Effective dose | Side effects | 🞏Yes 🞏 No |
|  |  |  |  |  |
| 1. **Experimental procedure:** The PI should mention within the experimental procedure section the following items: | | | |  |
| - The site and date of collection of plants | | | | 🞏Yes 🞏 No |
| - The taxonomical identification of plants by specialized Taxonomist | | | | 🞏Yes 🞏 No |
| - Which part of the plant will be used (i.e., plant organ(s) used in the research). | | | | 🞏Yes 🞏 No |
| - The safety procedures taken to protect or mitigate the side effects of herbal extracts, fractions or compounds | | | | 🞏Yes 🞏 No |
| - The efficacy of herbal samples | | | | 🞏Yes 🞏 No |

| **Section (D): Application for Research Studies involving Biospecimens** | | |
| --- | --- | --- |
|  | | |
| *Biospecimens (biological specimens) include cells, DNA, RNA, recombinant DNA, primers, nucleotides, tissues, organs, blood, urine, teeth, hair, nail clippings, body fluids and effusions e.g. Plasma, serum, lymphatic, amniotic, peritoneal, pleural, cerebrospinal etc.* | | |
| 1. **Origin of biospecimen**  🞏 Human 🞏 Animals 🞏 Plants 🞏 Microbiology | | 🞏Yes 🞏 No |
| - Specify the biospecimens and their amounts in your research study even if you did not collect these materials | | 🞏Yes 🞏 No |
| 🞏 Cells | Count = | |
| 🞏 Tissues | dimensions x x = cc | |
| 🞏 Organs | Number = | |
| 🞏 Teeth | Number = | |
| 🞏 Blood | volume= … ml | |
| 🞏 Urine | volume= … ml | |
| 🞏 Body fluids and effusions | volume= … ml | |
| 🞏 Hair | amount | |
| 🞏 Nail clippings | amount | |
| 🞏 DNA and RNA, primers, nucleotides |  | |
| 🞏 Recombinant DNA |  | |
| 🞏Others, please specify type and amount |  | |
| 1. **Does the study involve** *(you can choose more than one item)*   🞏 Access to existing data sets or databanks  🞏 Human genetic research  🞏 Cell therapy  🞏 Exposure of human subjects to ionizing radiation, infections, environmental and occupational hazards, toxic materials, pharmacologic agents  🞏 Genetically modified cells  🞏 Use of gametes or use or creation of embryos  🞏 Use of drugs, alternative / complementary therapies; or surgical, or other therapeutic or diagnostic procedures and devices  🞏 An innovation or intervention which is not traditional practice in the research population  🞏 Developing a new cell line  🞏 Need additional material to be stored for c*onfirmation of origin (authentication)/ histopathological confirmation/ normal tissue for comparison.*  🞏 A cell line from another laboratory or cell banks  🞏Derivation of a new cell line  🞏 Using human embryonic stem cells  🞏 Use of human cell lines as therapeutic agents  🞏 Material-transfer agreements from other organization  🞏 Continuous cell lines  🞏 Other type of research not specified above: Please, specify …. | | |

| 1. **Does the biospecimen linked to any of the following identifiers?**   🞏 Names or any geographic subdivisions smaller than a governorate/state, including street, address, city, etc.  🞏 All elements of dates related directly to an individual, including birth date, visit date, admission date, discharge date, date of death  🞏 Telephone, Fax numbers and /or Electronic mail addresses  🞏 Medical record numbers or any Health plan beneficiary numbers  🞏 Certificate/license numbers, Device identifiers and serial numbers  🞏 Web universal resource locators (URLs) or Internet protocol address numbers  🞏 Biometric identifiers, including fingerprints and voiceprints or full-face photographic images and any comparable images.  🞏National ID or any other unique identifying number, characteristic, or code, unless otherwise permitted by the Privacy Rule for re-identification  🞏None of the above |
| --- |

| 1. **Specify the identification state of biospecimens in your research**   🞏 Identifiable biospecimen with private/ medical information: linked to specific live individuals or private data of a “human subject”  🞏 Identified cell lines or DNA information (private information) that can be associated with individuals.  🞏 Identifiable biospecimen with publicly available information involves collection or study of existing data, documents, records, pathological specimens, diagnostic specimens, death certificates, etc.  🞏De-identified/ coded biospecimen: cannot be linked to specific live individuals or private data of a “human subject”  (With no attached data or missing identifiers from data)  🞏Re-identification of de-identified data | 🞏Yes 🞏 No |
| --- | --- |
| 1. **Mention the source of identifiable biospecimen**   🞏 Hospital 🞏 Clinic 🞏 Laboratory 🞏 Other, please specify …………. | 🞏Yes 🞏 No |
| 1. **Specify the source of coded biospecimens** *(de-identified/ not identifiable if there are legal requirements prohibiting release of the code key to the researcher)* 2. Specimen provider: ………………………. 3. Biobank: ……………………………. 4. Research center/institute/department: …………………… 5. University/ faculty/ department: …………….   Other, please specify …………. | |
| Carefully describe the mitigation strategies to protect the biospecimen from being exposed to spoilage or spreading infection? | |
| 1. **Are biospecimens to be used for commercial purposes:** 🞏**Yes** 🞏 **No**   يحظر الاتجار- بأي صورة كانت – بأي عينات بشرية تم الحصول عليها بغرض استخدامها في البحوث الطبية | |
| 1. **What is the expected duration of storage (if there will be) of biospecimens?** ……   لا يجوز تخزين تلك العينات بعد الانتهاء من البحث الطبي أو المواد الفائضة منها لغرض استخدامها في بحوث مستقبلية لأي غرض دون الحصول مسبقا على موافقة المجلس الأعلى لمراجعة أخلاقيات البحوث الطبية الإكلينيكية وموافقة مستنيرة من المبحوث أو ممثله القانوني | |

| 1. **Does the study involve stored biospecimens from another research?** 🞏 **Yes** 🞏 **No**   في حالة الإجابة بنعم فيجب الحصول مسبقا على موافقة المجلس الأعلى لمراجعة أخلاقيات البحوث الطبية الإكلينيكية وموافقة مستنيرة من المبحوث أو ممثله القانوني على استخدام العينات بعد انتهاء البحث السابق أو المواد الفائضة منه |
| --- |
| 1. **Does the study involve any residual specimens even if obtained for routine patient care that would have been discarded if not used for research?** 🞏 **Yes** 🞏 **No**   في حالة الإجابة بنعم فيجب الحصول موافقة مستنيرة من المبحوث أو ممثله القانوني وموافقة لجنة الأخلاقيات على استخدام هذه المواد التشخيصية الفائضة في البحث |
| 1. **Will human biospecimens be sent outside Egypt?**   🞏 **Yes** 🞏 **No** If yes, specify country/ destination institute …………………..  في حالة موافقة لجنة الأخلاقيات يتم العرض على المجلس الأعلى وعلى الأمن القومي |

| 1. **Will human biospecimens be imported to Egypt?** 🞏 **Yes** 🞏 **No**   If yes, specify country/ destination institute ……………………  في حالة موافقة لجنة الأخلاقيات يتم العرض على المجلس الأعلى وعلى الأمن القومي | | | |  |
| --- | --- | --- | --- | --- |
| 1. **Describe the method of disposal of residual human biospecimens:** ………………   وأن يتم ذلك وفقا للمعايير الدولية وبحضور أحد مفتشي هيئة الدواء المصرية وتقديم شهادة بذلك للجنة الأخلاقيات بعد انتهاء البحث | | | |  |
| 1. **Will research participants receive results of genetic testing or other research results?** | | | |  |
| 🞏 **Yes** 🞏 **No** | | | |  |
| 1. **Requesting waiver of consent or altering the elements of consent in your research,** please choose the applicable items to your research:  - The research poses minimal risk to human subjects - Consenting is not feasible in this research - The waiver or alteration will negatively affect the rights and welfare of subjects. - The subject or legal representative may be provided later by some related information whenever appropriate. - The research involves using identifiable biospecimens which could not practicably be carried out without using such information or biospecimens in an identifiable format. - The research involves an emergency care - Retrospective medical care records review - Difficulty with surrogate consent/legally authorized representative - other limited circumstances. please, specify: … - Others please, specify: … | | | |  |
| *MREC Reviewer Comment* | *Approve* | *Needs Explanation* | *Disapprove* | |
| Completely fulfilled and informative data | 🞏 | 🞏 | 🞏 | |
| Comments to the applicant |  |  |  | |
| Comment to the Committee |  |  |  | |

**INVESTIGATORS DECLARATION**

**Commitment**

**Project title: Evaluation of the ameliorative Effect of *Casuarina equisetifolia* Bark Extract after Incorporating Gold Nanoparticles against Toxicity Induced by Pesticides in Rats**

🞏 I the undersigned have read the and accept responsibility for the conduct of the procedures detailed in this proposal in accordance with the guidelines contained in the Helsinki declaration and all the related laws and guidelines in Egypt and Locally at the National Research Centre.

🞏 I understand that I must notify the MREC of the NRC through the amendment process of any changes in the research, including the changes of personnel, the number of those involved, or procedures performed, and understand that no additional procedures can be started without prior explicit approval from the MREC.

🞏 I shall make all the necessary procedures to ensure that the research team will comply with any other condition laid down by the MREC.

🞏 I the undersigned have read and understood this commitment and being the principal investigator accept all ethical and legal responsibility for not abiding by it. I shall secure the rights of all those who participated during the conduct of this research upon submitting by any member of the research team or me any paper for publication or presenting the results of this study before a conference or meeting or seminar or workshop etc. whether, it includes the whole or part of the results.

🞏 I will conserve a voucher specimen in the herbarium of NRC in studies involving medicinal plants.

**Name of Principal Investigator Signature**

**Ass. Prof. Wael Mahmoud Aboulthana …..……………………….**

Date: 06 / 11/ 2022

**تــعـــــهـــد**

🞏 أتعهد أنا الموقع أدناه قد أنني قد قرأت وفهمت هذا الالتزام وأوافق على المسؤولية الأخلاقية والقانونية لعدم الالتزام به و ألتزم بأن يتم تنفيذ الإجراءات الواردة في هذا المقترح البحثي وفقًا للإرشادات الواردة في إعلان هلسنكي وجميع القوانين واللوائح و القرارات ذات الصلة في جمهورية مصر العربية أو الصادرة من المركز القومي للبحوث.

🞏 أتفهم أنه يجب عليً إخطار لجنة أخلاقيات البحوث الطبية في المركز القومي للبحوث بأي تعديل أو تغييرات في البحث، بما في ذلك التغييرات في المشاركين، أو عدد الأشخاص المشاركين، أو الإجراءات التي سيتم إجراؤها، وأدرك أنه لا يمكن البدء في أي إجراءات إضافية بدون صريح مسبق و موافقة من لجنة أخلاقيات البحوث الطبية0

🞏 أتعهد بأن أتخذ جميع الإجراءات اللازمة لضمان امتثال فريق البحث لأي شرط آخر تضعه لجنة أخلاقيات البحوث الطبية.

🞏 أتعهد بتأمين حقوق كل من شارك في إجراء هذا البحث حتي و لو جزئيا عند تقديم أي من أعضاء الفريق البحثي أو مني أي ورقة للنشر أو تقديم نتائج هذه الدراسة قبل مؤتمر أو اجتماع أو ندوة أو ورشة عمل إلخ. ، حتي لو كان يتضمن جزء من النتائج.

🞏 في حالة الدراسات التي تستخدم نباتات طبية فأتعهد بحفظ عينة من النبات المستخدم في الدراسة في معشبة المركز القومي للبحوث

| **إسم الباحث الرئيس:** | **التوقيع:** |
| --- | --- |
| أ.م.د/ وائل محمود كامل أبو الثنا | .......................................................... |

التاريخ : 06/ 11/ 2022

| **التوصية** | | | **Recommendation** | |
| --- | --- | --- | --- | --- |
|  |  | | | |
|  | **الموافقة على الدراسة المُقدمة** | ❑ | | - ***Approval*** |
|  |  | List of non-binding suggestions, if relevant | | |
|  |  | | | |
|  | **الموافقة ستتم بعد تعديل النقاط التالية** | ❑ | | - ***approval after corrections*** |
|  | **نقاط التعديل هي** | - List of modifications | | |
|  |  |  | | |
|  |  |  | | |
|  |  |  | | |
|  |  |  | | |
|  |  | | | |
|  | **التأجيل للعرض كاملا لإعادة تقييمه** | ❑ | | ***deferment*** ***for full review*** |
|  | **للأسباب التالية** | List of issue | | |
|  |  |  | | |
|  |  |  | | |
|  |  |  | | |
|  |  |  | | |
|  |  | | | |
|  | **الرفض** | ❑ | | ***Disapproval*** |
|  | **للأسباب التالية** | List issues: | | |
|  |  |  | | |
|  |  |  | | |
|  |  |  | | |
|  |  |  | | |
|  |  | | | |
|  | **hs**  **اسم القائم بالتحكيم**  **التوقيع**  **التاريخ** | **Printed Reviewer Name**  ***Signature***  ***Date*** | | |
|  | | |  | |
